# ISUOG/ESGO Consensus Statement on ultrasound‐guided biopsy in gynecological oncology

**DOI:** 10.1002/uog.29183

**Published:** 2025-03-21

**Authors:** D. Fischerova, F. Planchamp, J. L. Alcázar, P. Dundr, E. Epstein, A. Felix, F. Frühauf, G. Garganese, I. Salvesen Haldorsen, D. Jurkovic, R. Kocian, D. Lengyel, F. Mascilini, A. Stepanyan, M. Stukan, S. Timmerman, T. Vanassche, Z. Yuan Ng, U. Scovazzi

**Affiliations:** ^1^ Department of Gynecology, Obstetrics and Neonatology, First Faculty of Medicine Charles University and General University Hospital in Prague Prague Czech Republic; ^2^ Institut Bergonie Bordeaux France; ^3^ University of Navarra Pamplona Spain; ^4^ Hospital QuirónSalud Málaga Spain; ^5^ Department of Pathology, First Faculty of Medicine Charles University and General University Hospital in Prague Prague Czech Republic; ^6^ Department of Clinical Science and Education Karolinska Institutet, Södersjukhuset Stockholm Sweden; ^7^ iNOVA4Health, NOVA Medical School, Faculdade de Ciências Médicas, NMS, FCM Universidade NOVA de Lisboa Lisbon Portugal; ^8^ Instituto Portugues de Oncologia de Lisboa Francisco Gentil Lisbon Portugal; ^9^ Unità Operativa di Chirurgia dei Organi Genitali Esterni Femminili, Divisione di Ginecologia Oncologica, Dipartimento Scienze della Salute della Donna, del Bambino e di Sanità Pubblica Fondazione Policlinico Universitario A. Gemelli IRCCS Rome Italy; ^10^ Gemelli Women Health Center for Digital and Personalized Medicine, Dipartimento Scienze della Vita e Sanità Pubblica Università Cattolica del Sacro Cuore Rome Italy; ^11^ Mohn Medical Imaging and Visualization Centre (MMIV), Department of Radiology Haukeland University Hospital Bergen Norway; ^12^ Section for Radiology, Department of Clinical Medicine University of Bergen Bergen Norway; ^13^ EGA Institute for Women's Health University College London London UK; ^14^ Department of Gynaecology National Institute of Oncology Budapest Hungary; ^15^ Doctoral School of Clinical Medicine University of Szeged Szeged Hungary; ^16^ Gynecologic Oncology Service, Nairi Medical Center National Institute of Health Yerevan Armenia; ^17^ Department of Gynecological Oncology Pomeranian Hospitals (Szpitale Pomorskie) Gdynia Poland; ^18^ Clinic of Surgical Oncology, Faculty of Health Sciences with the Institute of Maritime and Tropical Medicine Medical University of Gdansk Gdansk Poland; ^19^ Department of Development and Regeneration KU Leuven Leuven Belgium; ^20^ Department of Cardiovascular Diseases University Hospitals Leuven Leuven Belgium; ^21^ Department of Gynaecological Oncology KK Women's and Children's Hospital Singapore; ^22^ Academic Unit of Obstetrics and Gynecology Hospital Polyclinic San Martino and University of Genoa Genoa Italy

## Abstract

The International Society of Ultrasound in Obstetrics and Gynecology (ISUOG) with the European Society of Gynaecological Oncology (ESGO) jointly developed clinically relevant and evidence‐based statements on performing ultrasound‐guided biopsies in gynecological oncology.

The objective of this Consensus Statement is to assist clinicians, including gynecological sonographers, gynecological oncologists and radiologists, to achieve the best standards of practice in ultrasound‐guided biopsy procedures. ISUOG/ESGO nominated a multidisciplinary international group of 16 experts who have demonstrated leadership in the use of ultrasound‐guided biopsy in the clinical management of patients with gynecological cancer. In addition, two early‐career gynecological fellows were nominated to participate from the European Network of Young Gynae Oncologists (ENYGO) within ESGO and from ISUOG. The group also included a patient representative from the European Network of Gynaecological Cancer Advocacy Groups. The document is divided into six sections: (1) general recommendations; (2) image‐guided biopsy (imaging guidance, sampling methods); (3) indications and contraindications; (4) technique; (5) reporting; and (6) training and quality assurance. To ensure that the statements are evidence‐based, the current literature was reviewed and critically appraised. Preliminary statements were drafted based on this review of the literature. During a conference call, the whole group discussed each preliminary statement, and a first round of voting was carried out. The group achieved consensus on all 46 preliminary statements without the need for revision.

These ISUOG/ESGO statements on ultrasound‐guided biopsy in gynecological oncology, together with a summary of the evidence supporting each statement, are presented herein. This Consensus Statement is supplemented by detailed narrated videoclips presenting different approaches and indications for ultrasound‐guided biopsy, a patient leaflet, and an extended version which includes a detailed review of the evidence. © 2025 The Authors. Published by John Wiley & Sons Ltd on behalf of The International Society of Ultrasound in Obstetrics and Gynecology (ISUOG) and by Elsevier Inc. on behalf of the European Society of Gynaecological Oncology and the International Gynecologic Cancer Society.

## INTRODUCTION

There has been increasing use of minimally invasive diagnostic procedures in gynecological oncology in recent years, which confers the advantage of accurate diagnosis while minimizing procedure‐associated morbidity^1^, ^2^, ^3^. These diagnostic procedures are often indispensable to inform patient management in situations such as unresectable advanced gynecological cancer or suspected disease recurrence. There are no specific guidelines available to assist gynecologists in performing ultrasound‐guided biopsies. Real‐time biopsy guidance using a transvaginal or transrectal ultrasound approach allows close proximity to gynecological tumors, ensuring high diagnostic adequacy and accuracy, while the low risk of procedure‐related complications enables the biopsy to be performed in the outpatient setting. In addition, avoiding patient referral to other physicians such as interventional radiologists can reduce healthcare costs, limit patient distress and shorten the time to initiation of appropriate treatment.

In parallel with the introduction and improvement of ultrasound diagnosis in gynecological oncology, a broad spectrum of minimally invasive ultrasound‐guided diagnostic and therapeutic procedures has been developed (Table 1). Ultrasound‐guided diagnostic procedures include core‐needle (tru‐cut) biopsy and fine‐needle aspiration. Any ultrasound‐guided intervention changes the ultrasound modality from a risk‐free method to an intervention that carries risk and therefore needs its own clear standard operating procedure. Given an appropriate indication and careful execution, these procedures are well‐tolerated by patients and less risky and costly than surgical procedures^4^, ^5^, ^6^, ^7^, ^8^, ^9^, ^10^, ^11^. Until recently, common practice has been for ultrasound‐guided procedures to be performed mainly by interventional radiologists. There have been many useful guidelines published on the topic of interventional ultrasound using the percutaneous ultrasound‐guided approach. However, these guidelines focusing on intra‐abdominal interventions (including biopsies of, for example, liver, kidney, pancreas, spleen) do not address the pelvic organs fully^12^, ^13^, ^14^, ^15^, ^16^. Moreover, the transvaginal approach is rarely included in the armamentarium of radiologists. Scientific data from radiologic departments suggest underestimation of the applicability of transvaginal ultrasound‐guided biopsy in a gynecological oncology setting, with limited case numbers over a long period of time^17^.

**Table 1:** Image‐guided procedures in gynecological oncology

Procedure	Type of intervention
Diagnostic	Fine‐needle aspiration Core‐needle (tru‐cut) biopsy
Diagnostic/therapeutic	Drainage of fluid collections* Paracentesis Thoracocentesis
Palliative	Insertion of permanent peritoneal or thoracic catheter

* Fluid collections can occur in different clinical scenarios, including abscess, lymphocele, peritoneal pseudocyst, seroma and hematometra following trachelectomy or brachytherapy.

The lack of information on best practice in ultrasound‐guided biopsy in gynecology and the growing need for this service in every gynecological oncology center led to the initiation of the process that produced this Consensus Statement. The objective of this work is to assist clinicians, including gynecological sonographers, gynecological oncologists and radiologists, to achieve the best standards of practice in diagnostic (biopsy) procedures. It includes the following sections:

1. General recommendations
2. Image‐guided biopsy (imaging guidance modalities, adequacy, accuracy, diagnostic yield and complications of sampling methods)
3. Indications and contraindications
4. Technique
5. Reporting
6. Training and quality assurance

A comprehensive summary of published data for all six sections is provided in the extended version of this Consensus Statement (Appendix S1). The technique is demonstrated in Videoclips [Link], [Link], [Link] and the indications for biopsy in Videoclip S4. A sample patient leaflet is also provided to aid counseling and communication with patients (Appendix S2).

## RESPONSIBILITIES

The present series of statements represent a consensus of the authors regarding their currently accepted approaches for ultrasound‐guided biopsy, based on the available literature and evidence. Any clinician applying or consulting these statements is expected to use independent medical judgment in the context of individual clinical circumstances to determine all patients' care and treatment. These statements are presented without any warranty regarding their content, use or application and the authors disclaim any responsibility for their application or use in any way.

## METHODS

These consensus statements on ultrasound‐guided biopsy in gynecological oncology were developed using an eight‐step process, chaired by Professor Daniela Fischerova (Figure S1 in Appendix S1).

Aiming to assemble a multidisciplinary international group, the International Society of Ultrasound in Obstetrics and Gynecology (ISUOG) and the European Society of Gynaecological Oncology (ESGO) nominated 16 experts who have demonstrated leadership in the use of ultrasound‐guided biopsy in the clinical management of patients through research, administrative responsibilities and/or committee membership. Altogether, six gynecologists with special interest in ultrasonography, one radiologist, two pathologists, one cardiologist and six gynecological oncologists were included. In addition, two early‐career gynecological fellows with special interest in ultrasound‐guided biopsy were nominated to participate from the European Network of Young Gynae Oncologists (ENYGO) within ESGO and from ISUOG, to form the final working group of 18 participants. The participants did not represent the societies from which they were elected but were asked to base their decisions on their own experience and expertise. A patient representative from the European Network of Gynaecological Cancer Advocacy Groups (ENGAGE Co‐Chair) was also included in the group.

An initial conference call including the whole group was held to facilitate introductions, as well as to review the purpose and scope of the Consensus Statement. The proposed document was divided into six sections, each with a lead author and a working group according to previously expressed interests and expertise. To ensure that the statements were evidence‐based, the current literature was reviewed and critically appraised. A systematic literature review of relevant studies published between technique inception and 2023 was carried out using the MEDLINE database (Appendix S3). The literature search was limited to publications in the English language. Priority was given to high‐quality systematic reviews, meta‐analyses and validating cohort studies, although studies with lower levels of evidence were also evaluated. The search strategy excluded editorials, letters and case reports. The reference list of each identified article was reviewed for other potentially relevant articles. The results of the literature search were distributed to the whole group, including electronic full‐text versions of each article. One of the authors (F.P.) provided methodology support throughout the process, but did not participate in the voting on the consensus statements. Thus, there were 18 voting participants.

Each lead author, following discussion with their working group, was responsible for drafting preliminary statements after a review of the relevant literature. These were then circulated to the entire group prior to a second conference call. During the second conference call, the whole group discussed each preliminary statement, and a first round of binary voting (agree/disagree) was carried out for each potential statement. All 18 participants took part in each vote, but they were permitted to abstain from voting if they felt they had insufficient expertise to agree/disagree with the statement or if they had a conflict of interest that could influence their vote. Statements would be removed if a consensus among group members was not reached. The voters had the opportunity to provide comments or suggestions together with their votes, which would require revision of the statement and a second round of voting. The group achieved consensus on all 46 preliminary statements without the need for revision and another round of voting. Thus, based on the results of the first round of voting, the statements were finalized. In the main text of this Consensus Statement, we present a summary of the supporting evidence, the finalized series of statements, and their levels of evidence and grades as described in Appendix S4. The extended version of this Consensus Statement, including a detailed evidence review, is provided in Appendix S1.

## RESULTS

### General recommendations

The purpose of performing minimally invasive biopsy procedures in gynecology is to obtain a representative tissue sample from suspected pathological processes to enable a morphological examination^11^, ^17^, ^18^, ^19^, ^20^. These biopsy procedures are particularly useful for patients not eligible for more invasive interventions or to accelerate the process from diagnosis to therapy without the need for a period of recovery^7^, ^18^. In addition, in the era of personalized medicine, obtaining biopsy samples from gynecological cancers, either primary or recurrent disease, has become increasingly important to obtain material for predictive testing and to plan subsequent targeted therapy^5^, ^11^, ^21^, ^22^. Evidence indicates that repeated biopsies at different times are well tolerated by patients^4^, ^5^, ^23^. The indication for biopsy, performance and reporting of biopsy, and final interpretation of results according to the clinical and imaging findings require multidisciplinary team expertise. This was recently highlighted by a National Cancer Institute panel, which reported that improving the communication between radiologists, oncologists and pathologists increases the probability of obtaining fit‐for‐purpose samples for pathological diagnosis and/or genomic analysis^24^. Multidisciplinary team discussion is also required if procedure‐related risk is deemed to be high or in the event of inconclusive biopsy results, to discuss possible alternative diagnostic options^25^.

**Statement 1**: Image‐guided tumor biopsy forms an integral component of the individualized treatment of gynecological cancers, especially in the context of disseminated disease, recurrence or the presence of surgical contraindications.

- Level of evidence: 3b
- Grade of statement: C
- Consensus: yes, 100% (*n* = 18); no, 0% (*n* = 0); abstain, 0% (*n* = 0)

**Statement 2**: Expertise and effective communication within a multidisciplinary team are essential to determine a proper indication and technical execution of an ultrasound‐guided biopsy, to maximize the safety of the procedure, the adequacy of the specimen and the accuracy of pathology reporting, and to optimize the integration of biopsy results with clinical and imaging findings.

- Level of evidence: 3b
- Grade of statement: C
- Consensus: yes, 100% (*n* = 18); no, 0% (*n* = 0); abstain, 0% (*n* = 0)

### Image‐guided biopsy (image‐guidance modalities, adequacy, accuracy, diagnostic yield and complications of sampling methods)

Image‐guided biopsy aims to sample a target tissue using a guided approach, by ultrasound or other imaging technology. Real‐time biopsy guidance is essential to optimize tissue sample acquisition. Obtaining biopsy specimens which are suitable for histopathological and molecular analysis should be quick and minimally invasive, and should pose a low risk of procedure‐related complications. Ideally, the procedure should be performed in the outpatient setting, to avoid delays in initiating appropriate treatment, while also ensuring diagnostic accuracy and safety^18^, ^19^, ^26^.

#### Image‐guidance modalities

**Figure 1:**
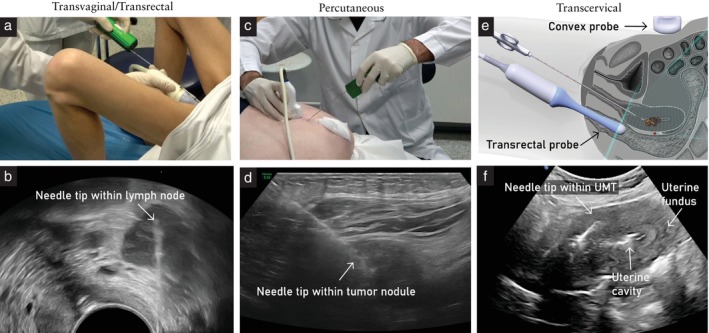
Illustration of different approaches for performing core‐needle biopsy. (a,b) Transvaginal or transrectal approach: the biopsy device with biopsy needle is inserted into a metal needle guide attached to the endocavitary probe (a); the biopsy is taken from an infiltrated pelvic parietal (iliac) lymph node (b). (c,d) Percutaneous approach, using the free‐hand technique: the biopsy needle is inserted along the longitudinal axis of the probe, guided by the ultrasound beam (c); the biopsy is taken from the infiltrated abdominal wall (Sister Mary Joseph's nodule) (d). (e,f) Transcervical approach: using an ultrasound probe placed in the rectum or on the abdomen for guidance (e), the biopsy needle is visible approaching the lesion transcervically and transcavitarily (in‐organ biopsy) (f). UMT, uterine mesenchymal tumor. See also Videoclips [Link], [Link], [Link].

Ultrasound offers many benefits, including a low rate of false‐negative biopsies and low rate of complications, wide availability, short procedure time, lack of ionizing radiation, portability and relatively low cost (Table S1 in Appendix S1)^27^. Crucially, it allows real‐time intraprocedural visualization of the biopsy needle and target lesion, dynamic multiplanar vision (i.e. the ability to guide the procedure in almost any anatomical plane), high soft‐tissue resolution (especially in the pelvis in the case of endovaginal/endoanal probe insertion) and the use of power or color Doppler, which are essential to achieve safe access to target lesions^18^, ^26^, ^27^, ^28^, ^29^, ^30^. Doppler examination helps to visualize blood vessels and define the most suitable part of the tumor for biopsy. Alternatively, contrast‐enhanced ultrasound can be used to evaluate the presence of vascularity as a sign of tumor tissue viability, especially in large intra‐abdominal tumors with areas of necrosis^21^, ^31^, ^32^. Ultrasound‐guided procedures can be done utilizing a variety of transducers (endocavitary, convex array, linear array and sector, among others), allowing different approaches for biopsy‐needle insertion (percutaneous, transvaginal, transcervical, transrectal) (Figure 1, Videoclips [Link], [Link], [Link])^18^, ^26^, ^29^. The safest path to the target lesion should be selected by avoiding non‐target organs and blood vessels. For biopsy of pelvic lesions, the transvaginal approach is preferred for its good diagnostic yield and safety profile, and should be considered the first choice, even when other approaches are feasible^17^, ^18^. Within the pelvis, the transrectal biopsy approach offers a short distance to the target and visualization similar to that of the transvaginal approach, but is less comfortable for patients and carries the potential risk of bacterial contamination^33^, ^34^, ^35^. It is crucial to explain carefully the reasons for recommending a transrectal biopsy approach and to obtain the patient's explicit consent before this approach is used. In the transcervical biopsy approach, for ultrasound guidance, the probe is placed in the rectum or on the abdomen, while the biopsy needle is inserted transcervically through the endocervical canal and advanced through the uterine cavity (transcavitary) to reach suspected uterine tumors without passing through the uterine serosa. This provides the advantage of an ‘in‐organ’ biopsy, which is important to minimize the risk of spread of malignant tumor cells along the needle‐biopsy tract^36^. The adequacy of biopsy using a percutaneous approach, with or without a needle guide, is highly dependent on adequate acoustic conditions and the location of the target lesion. The main limitations of percutaneous ultrasound guidance are related to the possible difficulty in visualizing the tumor target or needle tip. This may occur when the distance between the target and the ultrasound probe is large, such as: in obese subjects or in the presence of high‐volume ascites; when there is acoustic shadowing due to intestinal air or solid tissue, such as bone or calcified areas; or when the lesion is inaccessibile due to critical or vulnerable anatomical structures^7^, ^37^.

When visualization at ultrasound examination is problematic, contrast‐enhanced ultrasound, or coregistration (fusion imaging) of real‐time ultrasound with acquired images from computed tomography (CT), magnetic resonance imaging (MRI) or positron emission tomography (PET), may be considered, although these require specialized software and equipment and are less widely used^21^, ^38^, ^39^, ^40^.

The next most commonly used imaging method after ultrasound is CT^41^, ^42^, ^43^. It is a safe procedure with good diagnostic performance (accuracy estimates: 82–100%), but with major limitations related to the low tissue contrast, the need for patient fasting, the exposure to radiation and the risk of contrast‐agent‐related toxicity^43^. MRI guidance, despite providing good soft‐tissue resolution and having no risks associated with radiation exposure, is still used only rarely, as it requires special non‐magnetic equipment and experienced operators^44^. PET in combination with CT or MRI has also been proposed as guidance for biopsy; however, this is rarely used in gynecological practice^45^, ^46^.

#### Biopsy methods

**Figure 2:**
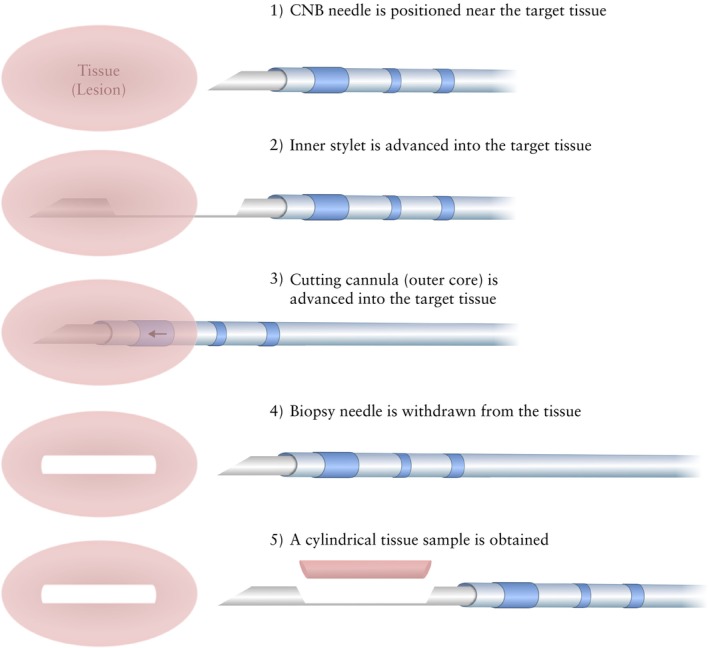
Illustration of core‐needle biopsy (CNB) mechanism, with a side‐cutting needle used to obtain biopsy sample. The tip of the biopsy needle should be positioned at the edge of or inside the lesion before firing, depending on the size of the lesion and the location of viable area(s) of tumor identified.

**Figure 3:**
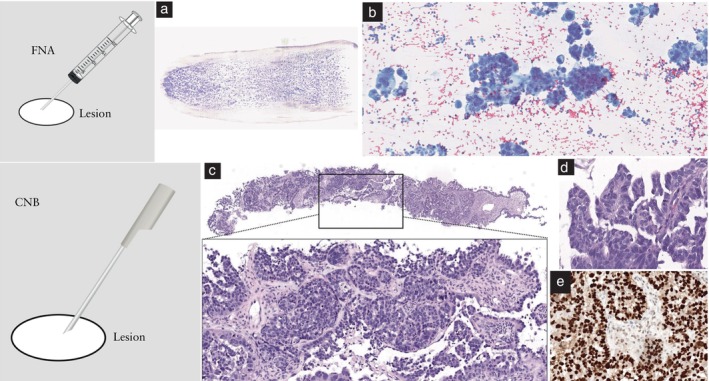
Comparison of fine‐needle aspiration (FNA) (a,b) and core‐needle biopsy (CNB) (c–e) specimens. (a) Macroscopic appearance of the cytological smear obtained from ascitic fluid (May–Grünwald–Giemsa stain) from a woman with findings suggestive of ovarian cancer and (b) high‐power view of carcinoma cells from the ascitic fluid (Papanicolaou stain). (c) Low‐power view of CNB specimen of the omentum (hematoxylin and eosin (H&E) staining) infiltrated by high‐grade serous carcinoma. (d) High‐power view of carcinoma cells with hyperchromatic and pleomorphic nuclei, and mitosis (H&E staining). (e) Immunohistochemical staining with p53 positivity in all cells, with strong and diffuse nuclear expression compatible with high‐grade serous carcinoma.

Two main sampling techniques are commonly used in gynecological oncology practice, applied to different cases according to the location of the target lesion, the type of lesion and its solid/fluid components, the clinical condition of the patient and other factors^47^, ^48^. The first, core‐needle biopsy (also known as tru‐cut biopsy), uses a side‐cutting or end‐cutting needle to provide tissue samples suitable for histological analysis and immunochemistry (Figure 2, and Figures S2–S4 in Appendix S1). The second, fine‐needle aspiration, also commonly referred to as fine‐needle aspiration cytology or biopsy, yields cells or, rarely, small tissue fragments, which are sufficient for cytological examination and may sometimes also be used for complementary studies if there is enough material (Figure 3, and Figure S5 in Appendix S1).

The use of biopsy specimens can also be extended to other clinical and research applications, such as molecular testing (e.g. complex genomic profiling by next‐generation sequencing) and other analyses (e.g. assessment of stromal microenvironment) (Table 2).

**Table 2:** Comparison between fine‐needle aspiration and core‐needle biopsy

	Fine‐needle aspiration	Core‐needle biopsy
Needle size	20–25 G (outer diameter from 0.9 mm to 0.5 mm)*	14–18 G (outer diameter from 2.1 mm to 1.3 mm)
Sample collection	Aspiration needle, often connected to a syringe whose plunger can be used to apply negative pressure to aspirate the specimen	Hollow needle with a spring‐loaded cutting action, integrated in an automated or semi‐automated gun
Sample type	Aspiration of cellular material or fluid from a lesion or effusion	Cylindrical/semicylindrical core of tissue from a solid mass
Material preservation/processing	The collected material is usually expelled onto multiple glass slides, creating smears, or into a container with a fixative, such as formalin; alternatively, medium for liquid‐based cytology can be used	The cylindrical/semicylindrical core of tissue from a solid mass is typically preserved in a fixative solution, most commonly formalin, and after fixation, the tissue core is processed to embed it in paraffin
Staining	The glass slides are typically stained with various specialized dyes (May–Grünwald–Giemsa stain; Papanicolaou stain) or H&E	The glass slides are typically stained with H&E; immunohistochemistry is commonly used
Microscopic examination	On microscopy, the pathologist evaluates cell morphology, structure and other characteristics, to reach a diagnosis	On microscopy, the pathologist assesses tissue architecture, cell morphology and other characteristics; this detailed examination aids in making a diagnosis
Advantages	Possibility of multiple passes in various directions for each sample Flexibility in specimen preparation Collection of fresh and intact cells Lower complication rate Low level of pain and rare need for local anesthesia Less expensive	Larger intact tissue sample with preserved architecture Tissue for immunohistochemistry and ancillary studies (e.g. NGS) Higher yield for fibrotic tissue lesions For most lesions, higher sensitivity, specificity and accuracy than fine‐needle aspiration to make a definitive diagnosis
Limitations	Limited tissue architecture Lower yield for fibrotic tissue lesions Difficult on cytological smears to distinguish e.g. borderline tumor from carcinoma Cytological specimen processing may be challenging; expertise required For most lesions, lower sensitivity, specificity and accuracy	More expensive Slightly higher level of pain and potential need for local anesthesia Higher complication rate Slightly longer tissue fixation and processing time

* Differences between fine‐needle aspiration and core‐needle biopsy are mainly due to the technique itself, rather than needle gauge.

H&E, hematoxylin and eosin staining; NGS, next‐generation sequencing.

Diagnostic performance of image‐guided biopsy is evaluated using the following parameters: adequacy (sufficient material for diagnosis), accuracy (concordance with final pathology), safety (low risk of complications (Table S2 in Appendix S1)) and grading of diagnostic yield (detection rate and impact on management). The literature shows no substantial differences between core‐needle biopsy and fine‐needle aspiration with respect to the adequacy of specimens obtainable (tissue block from core‐needle biopsy *vs* cytological smear from fine‐needle aspiration, 84–100% *vs* 74–100% of procedures provide adequate material) and their accuracy for distinguishing between benign and malignant tumors (73–100% *vs* 73–99% accuracy) (Tables S3 and S4 in Appendix S1)^7^, ^18^, ^19^, ^20^, ^49^, ^50^, ^51^, ^52^, ^53^, ^54^, ^55^, ^56^, ^57^, ^58^, ^59^, ^60^.

Core‐needle biopsy is preferred, because it allows a tissue sample to be obtained and thus more accurate assessment of the histological type of the tumor (Figure 3, and Figures S3 and S4 in Appendix S1). This is due in particular to the fact that core‐needle biopsy allows assessment of tumor architecture and the relationship between tumor and stroma, which is essential for correct diagnosis in some tumors^61^. Knowledge of the relationship between tumor and stroma is necessary in the evaluation of the biological nature of tumors such as low‐grade serous carcinoma and serous borderline tumor^62^. Diagnosis of some mesenchymal or fibrotic lesions may be difficult or impossible from fine‐needle aspiration samples, due to the low yield of tumor cells^63^. Moreover, a sufficient amount of material is needed for subsequent investigations, especially molecular tests.

Both techniques appear to be acceptable with regard to safety, with an overall rate of major complications of < 1.5% (Tables S2–S4 in Appendix S1)^7^, ^18^, ^19^, ^48^, ^64^, ^65^. The most frequently encountered complications are minor, and include procedure‐related pain or discomfort and self‐limiting biopsy‐related bleeding^50^, ^52^, ^61^, ^66^. Biopsy sample adequacy, accuracy and detection rate, the minimum amount of tissue required for sufficient analysis and possible complications (e.g. bleeding, infections, organ injury, vasovagal reaction and tumor seeding) are discussed in detail in the extended version of this document (Appendix S1).

It has been reported that increasing the biopsy core length (greater penetration depth), needle size (wider needles) and number of cores obtained (higher number of passes) directly influences the cancer detection rate, but may also lead to a greater risk of trauma and bleeding^67^, ^68^, ^69^. Data suggest that, for the molecular testing of epithelial tumors, at least two 10‐mm‐long cylinders using a needle that is 18‐G or wider should be obtained (Figure 3)^70^, ^71^. This amount of tissue is also likely to be sufficient for any other diagnostic purpose. For uterine mesenchymal tumors, the diagnostic yield reaches a plateau at three samples and does not appear to be improved by increasing the needle width greater than 18 G (Figure S3 in Appendix S1)^72^, ^73^, ^74^. Effort should be made to target the most heterogeneous area within the mesenchymal tumor tissue to increase diagnostic yield. When lymphoma is suspected, core‐needle biopsy may be considered as an alternative to the reference standard which is complete excision of the lymph node^26^, ^75^, ^76^. In these cases, a similar strategy, using an 18‐G needle with at least three passes through different regions of the lymph node or different lymph nodes, is recommended (Figure S4 in Appendix S1)^77^.

Given the advantages of core‐needle biopsy in gynecological oncology, the main emphasis in the following sections will be on this technique, although the indications, contraindications, biopsy technique, reporting and training are very similar to those for fine‐needle aspiration^4^, ^11^, ^18^, ^19^, ^20^, ^21^.

**Statement 3**: Image‐guided biopsy is a minimally invasive technique, which provides a safer alternative to surgery. It is effective in obtaining an adequate tissue sample for an actionable pathological result which can be used to guide treatment.

- Level of evidence: 3a
- Grade of statement: B
- Consensus: yes, 94% (*n* = 17); no, 0% (*n* = 0); abstain, 6% (*n* = 1)

**Statement 4**: Among all imaging methods, ultrasound should be considered the first choice for guidance of biopsy, because it provides real‐time imaging, is versatile and allows a multiplanar view.

- Level of evidence: 3b
- Grade of statement: B
- Consensus: yes, 94% (*n* = 17); no, 0% (*n* = 0); abstain, 6% (*n* = 1)

**Statement 5**: Ultrasound‐guided biopsy can be performed with different approaches (transvaginal, transcervical, transrectal and percutaneous), using different transducers (endocavitary, convex array and linear array) according to the safest path to the target and its best visualization.

- Level of evidence: 4
- Grade of statement: B
- Consensus: yes, 100% (*n* = 18); no, 0% (*n* = 0); abstain, 0% (*n* = 0)

**Statement 6**: Doppler examination may help to define the most suitable viable part of the tumor for biopsy. Alternatively, contrast‐enhanced ultrasound can be used.

- Level of evidence: 4
- Grade of statement: C
- Consensus: yes, 94% (*n* = 17); no, 0% (*n* = 0); abstain, 6% (*n* = 1)

**Statement 7**: In poorly visualized targets or when there are inconclusive findings with B‐mode ultrasound, alternative imaging techniques to guide the biopsy, such as contrast‐enhanced ultrasound or novel image fusion methods, can be employed.

- Level of evidence: 3a
- Grade of statement: C
- Consensus: yes, 78% (*n* = 14); no, 0% (*n* = 0); abstain, 22% (*n* = 4)

**Statement 8**: Other imaging guidance (CT, MRI or PET/CT) may be chosen according to lesion accessibility and/or to overcome suboptimal acoustic conditions.

- Level of evidence: 4
- Grade of statement: C
- Consensus: yes, 100% (*n* = 18); no, 0% (*n* = 0); abstain, 0% (*n* = 0)

**Statement 9**: Two sampling techniques can be used for image‐guided biopsy: core‐needle biopsy and fine‐needle aspiration.

- Level of evidence: 3a
- Grade of statement: B
- Consensus: yes, 94% (*n* = 17); no, 0% (*n* = 0); abstain, 6% (*n* = 1)

**Statement 10**: The choice between core‐needle biopsy and fine‐needle aspiration depends on the specific clinical situation. However, core‐needle biopsy is preferable to fine‐needle aspiration, as it allows tumor tissue to be obtained for biopsy examination, including ancillary methods, and it provides a larger volume of tumor tissue for multiple analyses, including molecular methods.

- Level of evidence: 4
- Grade of statement: C
- Consensus: yes, 94% (*n* = 17); no, 0% (*n* = 0); abstain, 6% (*n* = 1)

**Statement 11**: In order to ensure adequacy, high accuracy, diagnostic yield and safety of core‐needle biopsy, it is recommended to obtain at least two 10‐mm‐long cylinders using a needle that is 18 G or wider. This provides enough tissue for diagnostic, molecular and genetic purposes for epithelial tumors and their metastases (peritoneal, lymphatic, parenchymal). At least three such cores are recommended for uterine mesenchymal tumors and lymphomas.

- Level of evidence: 3b
- Grade of statement: C
- Consensus: yes, 89% (*n* = 16); no, 0% (*n* = 0); abstain, 11% (*n* = 2)

### Indications and contraindications

It is essential to ensure proper patient selection and valid clinical indication prior to image‐guided biopsy. The main indications and contraindications of core‐needle biopsy are shown in Table 3 and Figure S6 in Appendix S1, ^13^, ^15^, ^78^, ^79^. The indications for core‐needle biopsy are demonstrated in different clinical cases in Videoclip S4.

**Table 3:** Indications and contraindications for core‐needle biopsy in gynecology/gynecological oncology

*Indications for core‐needle biopsy* *
Primary inoperable/non‐resectable genital tumor (mostly ovarian/tubal cancer)
Cancer of unknown primary origin including metastases to genital organs (mostly secondary ovarian tumors)
Uterine mesenchymal tumor with atypical appearance on ultrasound or magnetic resonance imaging
Suspicious cervical or vaginal lesion (or, rarely, endometrial lesion if other methods of biopsy inapplicable)
Suspicion of recurrence of genital tumor
Research biopsy and molecular profiling (including *de novo* biopsy in case of disease progression or recurrence)
Staging purposes – inconclusive imaging findings
*Contraindications for core‐needle biopsy* †
Thrombocytopenia
Biopsy with low procedure‐related risk of bleeding when patient has elevated risk (PLT ≤ 30 × 10^9^/L)
Biopsy with high procedure‐related risk of bleeding when patient has elevated risk (PLT ≤ 50 × 10^9^/L)
Antiplatelet therapy (ongoing)‡
Coagulative disorder§
History of procedural bleeding or known bleeding tendency
Hemophilia
Abnormal coagulation screening tests (prothrombin time or activated partial thromboplastin time)
Anticoagulation therapy (ongoing)
Vitamin K antagonists¶
Biopsy with low procedure‐related risk of bleeding when patient has elevated risk (INR ≥ 2.0)
Biopsy with high procedure‐related risk of bleeding when patient has elevated risk (INR ≥ 1.5)
Direct oral anticoagulants (dabigatran, rivaroxaban, apixaban, edoxaban)¶
Difficult access to the lesion
Risk of tumor spillage (upstaging of well‐encapsulated mass due to iatrogenic intervention)
Absence of qualified operator, inadequate technical equipment, patient refusal or uncooperative patient**

* In descending order based on frequency; core‐needle biopsies for these indications are usually low‐risk procedures, while percutaneous biopsies of abdominal visceral organs (e.g. liver, kidneys) and biopsies of hypervascular lesions (color score, 4) are considered high‐risk procedures.

† Predominantly relative contraindications that can be controlled by periprocedural management of thrombotic and bleeding risks, or considered on a case‐by‐case basis; there are no absolute contraindications specific to core‐needle biopsy in gynecology/gynecological oncology.

‡ Risks and benefits of interruption of antiplatelet therapy should be considered, and periprocedural advice of treating specialist (hematologist/cardiologist/coagulation specialist) should be sought.

In general, the following algorithm is recommended: if antiplatelet therapy can be interrupted safely, stop antiplatelet therapy 5 days prior to biopsy; if continued antiplatelet therapy is indicated, and patient is undergoing single antiplatelet therapy, low‐risk procedures can be performed; if continued antiplatelet therapy is indicated, and patient is undergoing dual antiplatelet therapy, the risk of interrupting therapy is often high, and periprocedural management should be discussed with the treating specialist.

See Table 5 for further details.

§ Periprocedural advice from hematologist should be sought.

¶ See Table 5 for further details.

** General absolute contraindications of any interventional procedure; in the event of patient refusal, always check the patient's reasons carefully and explore whether there are concerns that can be addressed.

INR, international normalized ratio; PLT, platelet count.

The risk of the diagnostic procedure should not outweigh the potential benefits^13^, ^15^. Studies investigating core‐needle biopsy in gynecology demonstrated a low rate of major bleeding complications (< 1.5%) regardless of the approach^18^, ^19^, ^20^. Obtaining biopsies from richly vascularized intraperitoneal tumors and/or abdominal visceral organs is associated with an increased risk of complications^78^, ^80^. Patients with an elevated risk of bleeding include those with known bleeding disorders or prior bleeding complications, or those on anticoagulation or antiplatelet treatment^78^. For procedures with high risk of bleeding and/or patients at elevated general risk of bleeding, a routine screening coagulation panel is recommended, including hemoglobin, platelet count, prothrombin time (PT) and/or international normalized ratio (INR), and activated partial thromboplastin time (aPTT) (Table 4)^78^. In some patients, additional tests may be required, such as anti‐Xa testing in patients receiving heparin and fibrinogen level in patients with cirrhosis. In patients with bleeding tendencies or with a history of severe bleeding, normal PT and aPTT cannot rule out all coagulation disorders. If indicated, the preprocedural preparation should include appropriate counseling by a specialist to address the risks of bleeding and thromboembolism. More detailed information, for when there is comorbidity, can be found in the consensus guidelines of the Society of Interventional Radiology^78^.

**Table 4:** Current recommendations for screening coagulation panel and thresholds to perform biopsy procedure, in order to minimize risk of major bleeding complications, according to procedure‐related and patient‐related risks of bleeding

	Patient with low risk of bleeding	Patient with elevated risk of bleeding
Low procedure‐related risk of bleeding*	PT/INR, aPTT, Hb, PLT not routinely recommended	PT/INR, aPTT, Hb, PLT should be considered† Thresholds (correct to)‡: INR < 2.0 PLT > 30 × 10^9^/L
High procedure‐related risk of bleeding*	PT/INR, aPTT, Hb, PLT recommended routinely Thresholds (correct to)‡: INR < 1.5 PLT > 50 × 10^9^/L	PT/INR, aPTT, Hb, PLT recommended routinely† Thresholds (correct to)‡: INR < 1.5 PLT > 50 × 10^9^/L

* Biopsies for indications specified in Table 3 are usually low‐risk procedures, while percutaneous biopsies of abdominal visceral organs (e.g. liver, kidneys) and biopsies of hypervascular lesions (color score, 4) are considered high‐risk procedures.

† In addition, anti‐Xa testing in patients receiving heparin and assessment of fibrinogen level in patients with cirrhosis.

‡ Patient INR/PLT level should be corrected until threshold is met.

aPTT, activated partial thromboplastin time; Hb, hemoglobin; INR, international normalized ratio; PLT, platelet count; PT, prothrombin time.

For patients on anticoagulation and/or antiplatelet therapy, the decision whether to withhold the therapeutic agents prior to biopsy and, if so, for what length of time depend on the patient's overall clinical status and thromboembolic and bleeding risks and on the procedure‐associated bleeding risk^78^, ^81^. If the procedural bleeding risk is low, most anticoagulant/antiplatelet drugs can be continued. In such a situation, the patient's thromboembolic risk does not influence the clinical decision^82^. Conversely, for patients at elevated risk of bleeding and for procedures with high risk of bleeding, additional factors need to be considered, including the type of anticoagulant and antiplatelet agents used (Table 5)^80^, ^83^, ^84^, ^85^, ^86^. The final decision regarding the periprocedural management of anticoagulation, including the use of bridging therapy with low‐molecular‐weight heparin, should take into account and balance all the above risks^87^. High procedural risk, elevated bleeding risk, difficulty of access to the target lesion and other situations which increase biopsy‐related risk should be noted prior to the procedure. Such procedures should be performed by the most experienced operators, and the use of a thin core needle (18 G) should be considered to minimize tissue trauma.

**Table 5:** Recommendations for adjustment to specific anticoagulant and antiplatelet treatments in patients undergoing biopsy procedure

Anticoagulant or antiplatelet agent	Biopsy procedure with low risk of bleeding	Biopsy procedure with high risk of bleeding
Vitamin K antagonists (warfarin, phenprocoumon, acenocoumarol)	Consider continuation, check INR to exclude supratherapeutic levels (target INR < 2.0); if withheld, to reinitiate on same day as procedure	Withhold for 5 days until target INR < 1.5; consider bridging only in selected cases with very high risk of thrombosis; resume on day after procedure in the absence of bleeding complications*
LMWH (enoxaparin, nadroparin, tinzaparin, dalteparin)	Do not withhold, avoid peak plasma levels (perform biopsy ≥ 6 h after last dose of LMWH)	Withhold for 12 h for prophylactic doses of LMWH, and 24 h for therapeutic doses of LMWH Consider checking anti‐Xa if renal function impaired
Direct oral anticoagulants (dabigatran, rivaroxaban, apixaban, edoxaban)†	Do not withhold, avoid peak plasma levels (perform biopsy ≥ 6 h after last dose of direct oral anticoagulant) Skipping a single dose before and after the biopsy can be considered	Withhold 1–3 days before procedure (depending on agent and renal function‡) Resume 1–2 days after procedure in the absence of bleeding complications
Aspirin	Do not withhold	Withhold 3–5 days before biopsy, then resume on day after procedure†
Ticagrelor	Do not withhold	Withhold 5 days before biopsy; resume on day after procedure In patients with dual antiplatelet therapy (aspirin + ticagrelor), discuss with treating cardiologist/physician
Prasugrel	Do not withhold	Withhold 5 days before biopsy; resume on day after procedure In patients with dual antiplatelet therapy (aspirin + prasugrel), discuss with treating cardiologist/physician

* Warfarin will take 5–10 days to attain a full anticoagulant effect, as measured by an international normalized ratio (INR) > 2.0; therefore, consider use of a heparin ‘bridging therapy’ in patients at high risk of thromboembolism.

† For details on specific anticoagulant agents refer to current guidelines^78^, ^81^.

‡ Patients with impaired renal function may require longer; duration can be individualized.

LMWH, low‐molecular‐weight heparin.

**Statement 12**: Core‐needle biopsy should be performed if it is clinically meaningful for the patient's management. Indications include: (1) to determine primary origin in patients with inoperable/non‐resectable disease or unknown primary cancer; (2) to stage disease; (3) to identify residual or suspicious recurrent disease; (4) to establish the nature and histological diagnosis of suspicious uterine mesenchymal tumors; (5) to investigate suspicious cervical, vaginal or endometrial lesions and others; and (6) for targeted treatment including research purposes.

- Level of evidence: 2b
- Grade of statement: B
- Consensus: yes, 94% (*n* = 17); no, 0% (*n* = 0); abstain, 6% (*n* = 1)

**Statement 13**: There are no specific absolute contraindications to core‐needle biopsy in gynecology/gynecological oncology. However, risks and benefits should be balanced, considering the patient's comorbidities and medications, difficulty of access to the target lesion and risk of tumor spillage.

- Level of evidence: 4
- Grade of statement: C
- Consensus: yes, 100% (*n* = 18); no, 0% (*n* = 0); abstain, 0% (*n* = 0)

**Statement 14**: Any decision about periprocedural management should be based on a thorough assessment of the patient's overall clinical status, including thromboembolic and bleeding risks and the procedure‐associated risks.

- Level of evidence: 3b
- Grade of statement: C
- Consensus: yes, 100% (*n* = 18); no, 0% (*n* = 0); abstain, 0% (*n* = 0)

**Statement 15**: Clinicians performing a biopsy should be aware of potential complications and routinely implement strategies to avoid or minimize them.

- Level of evidence: 2b
- Grade of statement: B
- Consensus: yes, 100% (*n* = 18); no, 0% (*n* = 0); abstain, 0% (*n* = 0)

**Statement 16**: Regarding procedure‐related risk of major bleeding, core‐needle biopsies in gynecology/gynecological oncology are considered to be low risk (risk of major bleeding complication < 1.5%). Percutaneous biopsies of abdominal visceral organs (e.g. liver, kidneys) as well as any biopsy of hypervascular lesions (color score, 4) are considered high‐risk procedures.

- Level of evidence: 4
- Grade of statement: C
- Consensus: yes, 100% (*n* = 18); no, 0% (*n* = 0); abstain, 0% (*n* = 0)

**Statement 17**: Regarding patient‐related bleeding risk, women with coagulative disorders or on anticoagulative therapy and those with thrombocytopenia or on antiplatelet therapy are considered at elevated risk of major bleeding.

- Level of evidence: 4
- Grade of statement: C
- Consensus: yes, 100% (*n* = 18); no, 0% (*n* = 0); abstain, 0% (*n* = 0)

**Statement 18**: Withholding and restarting anticoagulant and/or antiplatelet drugs should be carried out according to recommendations of relevant specialists. The patient's individual thromboembolic risk should also be taken into consideration.

- Level of evidence: 4
- Grade of statement: C
- Consensus: yes, 100% (*n* = 18); no, 0% (*n* = 0); abstain, 0% (*n* = 0)

**Statement 19**: For procedures with low risk of bleeding planned in patients with no or minimal bleeding risk factors, a screening coagulation panel is not required. These procedures can be performed by adequately trained sonographers (level II minimum).

- Level of evidence: 3a
- Grade of statement: C
- Consensus: yes, 94% (*n* = 17); no, 0% (*n* = 0); abstain, 6% (*n* = 1)

**Statement 20**: For procedures with high risk of bleeding or in patients at elevated risk of bleeding, a screening coagulation panel (platelet count, hemoglobin, PT/INR and aPTT) is routinely recommended. These procedures should be performed by expert sonographers (level III).

- Level of evidence: 4
- Grade of statement: C
- Consensus: yes, 100% (*n* = 18); no, 0% (*n* = 0); abstain, 0% (*n* = 0)

**Statement 21**: For all procedures with high risk of bleeding, recommended laboratory thresholds to minimize the risk of major bleeding complications are INR < 1.5 and PLT > 50 × 10^9^/L.

- Level of evidence: 4
- Grade of statement: C
- Consensus: yes, 94% (*n* = 17); no, 0% (*n* = 0); abstain, 6% (*n* = 1)

**Statement 22**: For patients at elevated risk of bleeding undergoing procedures with low risk of major bleeding complications, laboratory thresholds are INR < 2.0 and PLT > 30 × 10^9^/L. Antiplatelet therapy can continue. Anticoagulant therapy can continue in most cases.

- Level of evidence: 4
- Grade of statement: C
- Consensus: yes, 89% (*n* = 16); no, 0% (*n* = 0); abstain, 11% (*n* = 2)

### Technique

**Figure 4:**
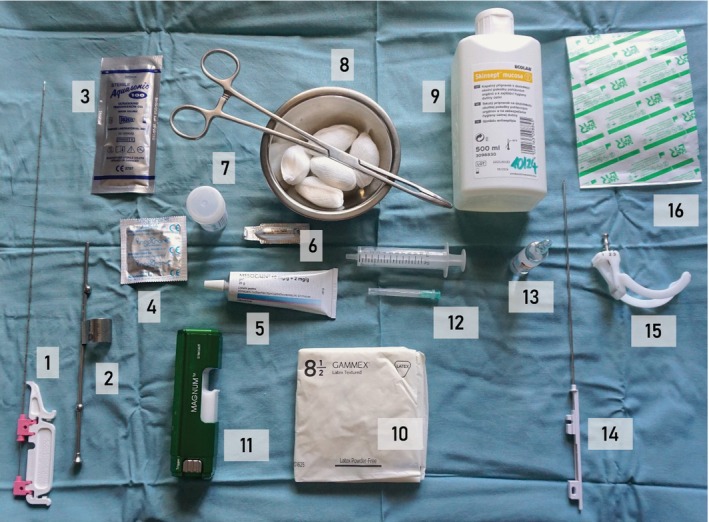
Instruments needed for core‐needle biopsy: (1) biopsy needle (30 cm/18 G); (2) needle guide for endocavitary probe; (3) sterile gel; (4) protective probe cover; (5) anesthetic gel; (6) analgesia suppository; (7) labeled specimen container; (8) basin, tongs and swabs for disinfection; (9) antiseptic cleaning agent; (10) sterile gloves; (11) automated core‐needle device; (12) needle and syringe for application of local anesthetic; (13) local anesthetic; (14) biopsy needle (20 cm/16 G); (15) needle guide for convex probe (optional); (16) wound covering. For transvaginal/transrectal biopsy procedure, instruments 1–11 are needed; for percutaneous biopsy procedure, instruments 3 and 7–16 are needed.

Ultrasound‐guided biopsy should be performed only by a physician familiar with the indications, contraindications, limitations, typical findings and possible side effects of the procedure. The physician should be trained in gynecological oncology sonography as well as ultrasound‐guided core‐needle biopsy and related safety issues, and should undertake quality assurance and control measures routinely. The choice of approach to guide the needle's path, caliber of the needle and penetration depth are dependent on the specific purpose and the safety of the procedure. The necessary instruments for core‐needle biopsy are shown in Figure 4. The steps for performing ultrasound‐guided biopsy are described in Table 6 and Videoclips [Link], [Link], [Link]. The characteristics and an illustration of different approaches are presented in Table S5 and Figures S7 and S8 in Appendix S1.

**Table 6:** Roadmap for performing ultrasound‐guided core‐needle biopsy (using automated device)

*Step 1: Patient preparation*
Ensure calm, comfortable environment and patient dignity.
Review indications for the procedure, multidisciplinary team recommendations and location for planned biopsy.
Obtain medical history, check for allergies and identify risk factors for potential complications.
Explain procedure, risks and benefits to the patient and obtain informed consent. Ask for and address any fears or worries.
Assess risk of bleeding (see Table 4).
Seek specialist advice on withholding anticoagulant/antiplatelet treatment as necessary (see Table 5).
Offer use of oral analgesics such as 1 g paracetamol or oral non‐steroidal anti‐inflammatory drug, or analgesia suppository (TV/TR/TC procedure) if desired by the patient.
Administer prophylactic antibiotics if indicated (e.g. in case of passage of the needle through the rectal wall into the peritoneal cavity, immunocompromised patient, risk of infective endocarditis).
*Step 2: Selection of biopsy site and approach*
Place patient in lithotomy (TV/TR/TC procedure) or supine (percutaneous) position and cover with a drape.
Perform an ultrasound examination to assess the feasibility of biopsy, identify target lesion, plan the access route to the target lesion and identify organs at risk of injury.
Use Doppler to identify viable tumor tissue, assess tumor vascularization and identify adjacent vascular structures.
Set up needle guidance line for TV and TR biopsies. Use of needle guide is optional for percutaneous biopsies.
Measure the penetration depth (distance from closest to farthest edge of the tumor to cover the tumor's full thickness in the planned direction of biopsy) and set biopsy device accordingly.
*Step 3: Infection prevention and instrument preparation*
Prior to use, clean and disinfect the ultrasound probes and machine.
Wash hands and perform antisepsis.
Prepare necessary instruments on instrument trolley (Figure 4).
Don sterile gloves.
Apply sterile gel to the transducer and enclose with a disposable transducer cover.
Apply local anesthetic gel to the tip of the covered ultrasound probe (for TV/TR procedure).
Affix needle guide to ultrasound probe (optional for percutaneous approach)
Attach biopsy needle to biopsy device and remove the spacer.
Charge device by pulling the lever and adjust penetration depth accordingly.
Test the firing of the device when in ‘FIRE’ mode then switch to ‘SAFE’ mode.
*Step 4: Performing the procedure (Videoclips* [Link], [Link], [Link] *)*
Monitor patient comfort throughout the procedure.
Disinfect the vagina or skin (TV and percutaneous procedures, respectively).
A rectal cleansing enema is optional (TR procedure).
Apply local anesthetic as indicated.
Percutaneous procedure: Under ultrasound guidance, administer local anesthetic injection to skin and abdominal wall in direction of the intended biopsy path up to the target tissue. The skin incision may be extended with a larger bore needle or scalpel for easier entry of the core needle if required.
TV/TR procedure: Insert the probe using a finger to guard the mucosal surface from the needle guide. Administration of local anesthetic along the needle trajectory using a long needle placed within the needle guide for anesthetic injection is optional.
TC procedure: A paracervical block can be used.
Hold the probe with one hand and introduce the needle with the needle guide with the other hand. For freehand percutaneous technique, insert needle along the longitudinal axis of the probe below the ultrasound beam.
Visualize needle tip continuously with ultrasound.
Align needle guidance line (if set up) with the lesion and insert needle to the nearest edge of the viable part of the lesion.
Switch the device to ‘FIRE’ mode and activate the trigger.
Place the specimen in formalin.
Repeat the procedure to obtain three biopsy cores. Between biopsy passes, the ultrasound probe should ideally be left in place, especially if the probe is inserted in the vagina or rectum.
After each sampling, an assistant helps to move the specimen to a formalin‐filled container using either a needle stylet or a normal saline flush.
Ultrasound should be used to detect any signs of internal bleeding.
Percutaneous procedure: Apply pressure at the biopsy site for a few minutes and then cover with a sterile dressing.
TV procedure: Apply puncture site pressure using a gauze swab in the vagina for 1–5 min after the puncture to stop vaginal bleeding if necessary.
*Step 5: Postprocedural requirements*
Give the patient time to sit up and dress, and assess her condition.
Inform the patient how and when the results of biopsy will be communicated, and provide written information about signs of potential complications and emergency contacts. Inform the patient of the use of postprocedural oral analgesia, if required.
In uncomplicated low‐risk procedures, no postprocedural monitoring is required.
Label specimen container with patient identifiers.
Fill pathology request form with relevant information:
∙ Patient identifiers
∙ Clinical data, radiological data and patient history
∙ Details of requesting doctor
∙ Biopsy site, type of specimen and fixative used
∙ Clinical impression and differential diagnosis
∙ Previous histopathological findings (if any)
∙ Specific requests to the pathologist according to clinical need (immunohistochemistry, molecular analysis or other processing)
Attach pathology request form to specimen container.

This table was formulated using both data from the literature and by consensus of the study authors^108^, ^109^, ^110^, ^111^, ^112^, ^113^.

TC, transcervical; TR, transrectal; TV, transvaginal.

**Statement 23**: Before the procedure, the indication should be verified and the biopsy deemed to be clinically relevant.

- Level of evidence: 3b
- Grade of statement: C
- Consensus: yes, 100% (*n* = 18); no, 0% (*n* = 0); abstain, 0% (*n* = 0)

**Statement 24**: An ultrasound examination should be performed to select the safest path to the target lesion and subsequent approach.

- Level of evidence: 4
- Grade of statement: C
- Consensus: yes, 100% (*n* = 18); no, 0% (*n* = 0); abstain, 0% (*n* = 0)

**Statement 25**: Core‐needle biopsy in gynecology does not require specific preparation such as fasting, or use of laxatives or antiflatulent medication.

- Level of evidence: 4
- Grade of statement: C
- Consensus: yes, 89% (*n* = 16); no, 0% (*n* = 0); abstain, 11% (*n* = 2)

**Statement 26**: The preparation should include providing the patient with procedure‐related information, obtaining informed consent and identifying relevant medical history.

- Level of evidence: 3b
- Grade of statement: C
- Consensus: yes, 100% (*n* = 18); no, 0% (*n* = 0); abstain, 0% (*n* = 0)

**Statement 27**: Bleeding risk assessment should be performed according to the procedure‐related and patient‐related risks of bleeding.

- Level of evidence: 4
- Grade of statement: C
- Consensus: yes, 100% (*n* = 18); no, 0% (*n* = 0); abstain, 0% (*n* = 0)

**Statement 28**: Antibiotic prophylaxis is not recommended routinely, as the risk of infectious complications is low (< 1%). However, it should be considered on an individual basis, such as for the transrectal approach, if the needle passes through the rectal wall into the peritoneal cavity.

- Level of evidence: 4
- Grade of statement: C
- Consensus: yes, 94% (*n* = 17); no, 0% (*n* = 0); abstain, 6% (*n* = 1)

**Statement 29**: Maximum attention should be paid to minimize patient discomfort, pain and anxiety throughout the procedure.

- Level of evidence: 2b
- Grade of statement: B
- Consensus: yes, 100% (*n* = 18); no, 0% (*n* = 0); abstain, 0% (*n* = 0)

**Statement 30**: Basic disinfection procedures are sufficient. It is important to perform hand antisepsis. Clean handling of sterile instruments (needles) is recommended. All instruments should be laid out on a sterile trolley. The disinfected ultrasound probe should be covered with a sterile, disposable protective cover.

- Level of evidence: 4
- Grade of statement: C
- Consensus: yes, 89% (*n* = 16); no, 0% (*n* = 0); abstain, 11% (*n* = 2)

**Statement 31**: The biopsy device, type of single‐use disposable needles, needle gauge, needle length and penetration depth should be based on the planned biopsy route.

- Level of evidence: 3b
- Grade of statement: B
- Consensus: yes, 94% (*n* = 17); no, 0% (*n* = 0); abstain, 6% (*n* = 1)

**Statement 32**: The puncture site should be cleansed with an antiseptic solution for transvaginal and percutaneous approaches.

- Level of evidence: 4
- Grade of statement: C
- Consensus: yes, 94% (*n* = 17); no, 0% (*n* = 0); abstain, 6% (*n* = 1)

**Statement 33**: Local anesthetic reduces discomfort caused by larger needle size and should be administered for all percutaneous biopsies. It can be administered optionally for transvaginal, transcervical and transrectal approaches.

- Level of evidence: 2b
- Grade of statement: B
- Consensus: yes, 94% (*n* = 17); no, 0% (*n* = 0); abstain, 6% (*n* = 1)

**Statement 34**: While performing the biopsy, the tip of the needle must be visible under continuous ultrasound control. If not, the procedure should be abandoned.

- Level of evidence: 5
- Grade of statement: D
- Consensus: yes, 89% (*n* = 16); no, 0% (*n* = 0); abstain, 11% (*n* = 2)

**Statement 35**: Specimen fixation is critical for proper assessment of the tissue. Core‐needle biopsy specimen should be placed immediately in a formalin fixative solution and sent to the pathology laboratory. Optimal fixation is essential for immunohistochemical and molecular analysis. A minimum fixation time of 6 h and a maximum of 72 h is recommended.

- Level of evidence: 3b
- Grade of statement: B
- Consensus: yes, 67% (*n* = 12); no, 0% (*n* = 0); abstain, 33% (*n* = 6)

**Statement 36**: Ultrasound should be used at the end of the procedure to detect any signs of bleeding. Mild internal bleeding usually resolves spontaneously.

- Level of evidence: 5
- Grade of statement: D
- Consensus: yes, 94% (*n* = 17); no, 0% (*n* = 0); abstain, 6% (*n* = 1)

**Statement 37**: Following uncomplicated procedures, there is no need for prolonged monitoring. The patient should be informed about the symptoms of potential complications and provided with a written information sheet.

- Level of evidence: 4
- Grade of statement: C
- Consensus: yes, 94% (*n* = 17); no, 0% (*n* = 0); abstain, 6% (*n* = 1)

### Reporting

#### Biopsy documentation

A detailed report regarding the procedure must be given to the patient and to her healthcare provider. The following data should be included: indication(s) for biopsy, preprocedural ultrasound findings, procedure description including biopsy device used, collection guidance (ultrasound) and approach (transvaginal/transrectal/transcervical/percutaneous), biopsy site, number of samples, adequacy of the sample, difficulty of the procedure, patient tolerance, any complications and when the results are expected to be communicated. Biopsy results may be available between 48 h and 10 days after delivery of the sample to the laboratory, depending on the complexity of tests required. Copies of the report and images or videoclips recording the position of the needle within the lesion should be stored for future reference.

#### Specimen and biopsy data sheet

The request form for pathological examination should contain the patient's history and clinical and radiological information in detail, including clinical diagnosis and differential diagnosis. In some situations, the biopsy is taken from multiple sites, thus all specimens must be sent separately, clearly labeled, and documented in the request form^88^. The patient details on the specimen container and request form must be correct and match. Data on the request form and specimen containers are checked in the pathology laboratory before handling the specimen and when reporting. A detailed pathological report will then be discussed by the multidisciplinary team, taking into consideration all relevant patient data (Table S6 in Appendix S1)^89^, ^90^, ^91^, ^92^, ^93^, ^94^.

**Statement 38**: The request form for pathological examinations should contain:

– Patient identifiers, including age, gender and unique ID of the patient, which can differ among countries.
– All relevant clinical, radiological and patient history data.
– Details of the requesting doctor and contact details in case of emergency.
– The biopsy site(s), clinical impression and differential diagnosis.
– Previous histopathological findings (if any).
– The type of specimen and type of fixative used.
– The patient details on the request form and specimen container must be correct and match.
  - Level of evidence: 4
  - Grade of statement: C
  - Consensus: yes, 89% (*n* = 16); no, 0% (*n* = 0); abstain, 11% (*n* = 2)

**Statement 39**: The pathology report form should contain:

– Identification of the patient.
– Type of specimen and sample description.
– Type of processing (for fine‐needle aspiration).
– Evaluation of the sample adequacy.
– Limitations.
– Diagnosis.
– Optional: microscopic description, immunohistochemical/immunocytochemical findings, molecular testing findings, differential diagnosis, recommendation.
  - Level of evidence: 3a
  - Grade of statement: B
  - Consensus: yes, 94% (*n* = 17); no, 0% (*n* = 0); abstain, 6% (*n* = 1)

**Statement 40**: Analytic turnaround time of 2 days (business days counted only) after receipt of the sample at the pathology laboratory is required. If ancillary techniques such as immunohistochemistry are needed, the turnaround time is longer.

- Level of evidence: 3b
- Grade of statement: B
- Consensus: yes, 72% (*n* = 13); no, 0% (*n* = 0); abstain, 28% (*n* = 5)

### Training and quality assurance

To maintain high quality and safety of image‐guided biopsies, competent operators who are skilled in invasive diagnostic techniques and anatomy are required. This is crucial when accessing deep lesions in the female pelvis, which can be challenging due to the proximity of the major vessels, urinary bladder, ureters and bowel. The operator should possess a high‐level understanding of both the theoretical and practical aspects of the imaging modality used for guidance and the interventional procedures. Developing the necessary skills and techniques involves a steep learning curve, and sufficient volume is required to maintain operator confidence^95^. Similarly, competent pathologists and cytopathologists are essential for accurate reading of the biopsy samples.

#### For gynecologists/radiologists

Learning interventional ultrasound should always be built upon the knowledge of diagnostic (non‐interventional) ultrasound imaging of the area of interest^96^. It is advisable that core‐needle biopsy is performed by examiners who already have an intermediate (European Federation of Societies for Ultrasound in Medicine and Biology level II) or advanced (level III) level of competence in gynecological ultrasound imaging^97^. For radiologists, training in interventional radiology is recommended^15^. Studies indicate that the learning curve can be shortened by adding simulator‐based or phantom‐based training to clinical practice^98^. Web‐based teaching resources are also available^99^. Clinical training should begin under supervision by an experienced operator and with ‘simple’ core‐needle biopsy or fine‐needle aspiration procedures. When performing percutaneous biopsy, commercially available sonographic guides attached to the transabdominal probe may provide more confidence regarding the needle position inside the body and are recommended for less experienced operators^100^, ^101^. There is a lack of large studies assessing the impact of examiner experience and training on complication rate when performing pelvic core‐needle biopsy. We recommend carrying out at least 20 core‐needle biopsy procedures, supervised by an experienced operator, before embarking on the procedure unsupervised.

After achieving competence, operators are recommended to continue to perform these procedures on a regular basis. It is therefore reasonable to adopt the same recommended minimal number, i.e. 20 core‐needle biopsies annually per operator.

There should be regular audits of sampling accuracy for malignant tissue and the rate of inadequate specimens submitted within each practice providing biopsy services^95^, ^102^. Similarly, reviews of complications and patient experience should be conducted, to identify and address the need for improvement and additional training of staff. Preprocedural provision of patient information, periprocedural psychosocial support, provision of a comfortable environment and postprocedural monitoring of patient‐reported complications may improve patients' experience.

#### For pathologists

Pathologists reporting biopsy samples should have completed their postgraduate training in pathology, according to the national authority rules^103^, ^104^.

Training competencies that pathologists should demonstrate include: (1) the ability to produce clear, concise, comprehensive and timely written reports for surgical pathology and cytopathology; (2) the ability to incorporate the diagnostic, prognostic or predictive implications of molecular pathology tests into an integrated pathology report; (3) promotion of health informatics to improve the quality of patient care and optimize patient safety; (4) participation in quality control, quality assurance and quality improvement initiatives; (5) utilization of genetic testing resources effectively to balance costs with potential utility of result; (6) alerting of the treating physician when inheritable conditions are identified (e.g. genetic disease that may affect the patient's family members).

To maintain competence in pathological reporting, pathologists or cytopathologists should be part of a gynecological oncology multidisciplinary team in a high‐volume center. As part of quality assurance, participation in accredited programs for all aspects of tissue diagnostics, for both clinical and non‐clinical laboratories and organizations, is recommended^105^, ^106^, ^107^.

**Statement 41**: Training for operators:

– Training to at least intermediate (level II) or advanced (level III) level in gynecological ultrasound imaging is essential before commencing training in interventional ultrasound.
– Training in ultrasound‐guided biopsy using phantoms and/or computer simulation improves skills and is useful prior to clinical training.
– Targeted training using directly supervised procedures is essential to reduce the risk of complications and increase sample adequacy.
– The needle‐guiding system should be used by trainees for all approaches.
– At least 20 directly supervised core‐needle biopsies using a needle guide should be performed before starting unsupervised work.
  - Level of evidence: 4
  - Grade of statement: B
  - Consensus: yes, 89% (*n* = 16); no, 0% (*n* = 0); abstain, 11% (*n* = 2)

**Statement 42**: Maintaining competence for operators:

– Maintain competency by completing or supervising a minimum of 20 core‐needle biopsy procedures annually.
– Seek support from a more experienced operator when difficulties are anticipated or encountered.
  - Level of evidence: 3b
  - Grade of statement: C
  - Consensus: yes, 83% (*n* = 15); no, 0% (*n* = 0); abstain, 17% (*n* = 3)

**Statement 43**: Audit for operators:

– Regular audits should be undertaken within each practice providing biopsy services to ensure sampling adequacy and diagnostic yield, and to record complications and patient experience.
  - Level of evidence: 3b
  - Grade of statement: C
  - Consensus: yes, 89% (*n* = 16); no, 0% (*n* = 0); abstain, 11% (*n* = 2)

**Statement 44**: Training for pathologists/cytopathologists:

– Biopsies should be read by a pathologist or cytopathologist who has completed his/her postgraduate training in pathology/cytopathology. The rules are defined by national authorities and can differ among countries.
  - Level of evidence: 5
  - Grade of statement: C
  - Consensus: yes, 89% (*n* = 16); no, 0% (*n* = 0); abstain, 11% (*n* = 2)

**Statement 45**: Maintaining competence for pathologists/cytopathologists:

– Ultrasound‐guided biopsies should be performed in a specialized center with access to a pathologist or cytopathologist with experience in gynecological oncology as part of a multidisciplinary team.
  - Level of evidence: 5
  - Grade of statement: C
  - Consensus: yes, 94% (*n* = 17); no, 0% (*n* = 0); abstain, 6% (*n* = 1)

**Statement 46**: Audit for pathologists/cytopathologists:

– Accreditation of laboratories should be in accordance with national or international standards (such as ISO15189). The rules of accreditation are defined by national authorities in each country and can differ.
  - Level of evidence: 5
  - Grade of statement: C
  - Consensus: yes, 89% (*n* = 16); no, 0% (*n* = 0); abstain, 11% (*n* = 2)

## CONCLUSION

Core‐needle biopsy under ultrasound guidance is an emerging, minimally invasive outpatient procedure. It allows collection of high‐quality specimens for histopathological diagnosis, immunohistochemical analysis and molecular testing, enabling the timely commencement of appropriate treatment within a specialized cancer center. Performing core‐needle biopsy and interpreting its results requires appropriate expertise and should be conducted within a multidisciplinary team. Under these circumstances, it is simple, quick, effective and safe. To ensure patient‐centered care, standard operational procedures, including measures to minimize patient anxiety, pain and risk, are essential. This Consensus Statement aims to facilitate the implementation of this technique in gynecological oncology practice and improve patient outcomes.

The decision to develop this Consensus Statement was made jointly by ISUOG and ESGO. ISUOG and ESGO are non‐profit societies. The development group (including all authors) is collectively responsible for the decision to submit for publication. D. Fischerova (chair), U. Scovazzi and F. Planchamp (methodologist) have written the first draft of the manuscript. All other contributors have actively given personal input, reviewed the manuscript, and have given final approval before submission.

## CITATION

This Consensus Statement should be cited as: ‘Fischerova D, Planchamp F, Alcázar JL, Dundr P, Epstein E, Felix A, Frühauf F, Garganese G, Salvesen Haldorsen I, Jurkovic D, Kocian R, Lengyel D, Mascilini F, Stepanyan A, Stukan M, Timmerman S, Vanassche T, Yuan Ng Z, Scovazzi U. ISUOG/ESGO Consensus Statement on ultrasound‐guided biopsy in gynecological oncology. *Ultrasound Obstet Gynecol* 2025; **65**: 517–535.’

## Supporting information


**Appendix S1** ISUOG/ESGO Consensus Statement on ultrasound‐guided biopsy in gynecological oncology (extended version, including supporting information figures and tables).
**Appendix S2** Sample patient leaflet.
**Appendix S3** Identification of scientific evidence.
**Appendix S4** Levels of evidence and grades of statement used in this Consensus Statement.

**Videoclip S1** Core‐needle biopsy (transvaginal procedure): live demonstration of the preprocedural preparation (including selection of the biopsy site and approach), hygiene and instrument setup, procedure execution and postoperative management of women undergoing transvaginal core‐needle biopsy.

**Videoclip S2** Core‐needle biopsy (transrectal procedure): live demonstration of the preprocedural preparation, hygiene and instrument setup, procedure execution and postoperative management of women undergoing transrectal core‐needle biopsy (cases in which the transvaginal approach is not feasible).

**Videoclip S3** Core‐needle biopsy (percutaneous): live demonstration of the preprocedural preparation, hygiene and instrument setup, procedure execution (including management of analgesia) and postoperative management of women undergoing percutaneous core‐needle biopsy.

**Videoclip S4** Overview of indications for core‐needle biopsy encountered in the gynecological oncology unit with presentation of clinical cases.

## Data Availability

The data that supports the findings of this study are available in the supplementary material of this article.
