# Supplementary material for: ISUOG/ESGO Consensus Statement on ultrasound‐guided biopsy in gynecological oncology
Source: Ultrasound Obstet Gynecol. 2025 Mar 21;65(4):517–35. doi: 10.1002/uog.29183 (PMC11961111; doi:10.1002/uog.29183)
Supplement: Supplementary file 1 — Appendix S1 ISUOG/ESGO Consensus Statement on ultrasound‐guided biopsy in gynecological oncology (extended version, including supporting information figures and tables). Appendix S2 Sample patient leaflet. Appendix S3 Identification of scientific evidence. Appendix S4 Levels of evidence and grades of statement used in this Consensus Statement. [file UOG-65-517-s001.docx]

**Appendices**

**Appendix S1.** ISUOG/ESGO Consensus Statement on ultrasound-guided biopsy in gynecological oncology (extended version, including supporting information figures and tables)

Authors: Daniela Fischerova^1^, François Planchamp^2^, Juan Luis Alcázar^3,4^, Pavel Dundr^5^, Elisabeth Epstein^6^, Ana Felix^7,8^, Filip Frühauf^1^, Giorgia Garganese^9,10^, Ingfrid Salvesen Haldorsen^11,12^, Davor Jurkovic^13^, Roman Kocian^1^, Daniel Lengyel^14,15^, Floriana Mascilini^9^, Artem Stepanyan^16^, Maciej Stukan^17,18^, Stefan Timmerman^19^, Thomas Vanassche^20^, Zheng Yuan Ng^21^, Umberto Scovazzi^22^

|  |
| --- |
| **ABSTRACT**  The International Society of Ultrasound in Obstetrics and Gynecology (ISUOG) with the European Society of Gynaecological Oncology (ESGO) jointly developed clinically relevant and evidence-based statements on performing ultrasound-guided biopsies in gynecological oncology.  The objective of this Consensus Statement is to assist clinicians, including gynecological sonographers, gynecological oncologists and radiologists, to achieve the best standards of practice in ultrasound-guided biopsy procedures. ISUOG/ESGO nominated a multidisciplinary international group of 16 experts who have demonstrated leadership in the use of ultrasound-guided biopsy in the clinical management of patients with gynecological cancer. In addition, two early-career gynecological fellows were nominated to participate from the European Network of Young Gynae Oncologists (ENYGO) within ESGO and from ISUOG. The group also included a patient representative from the European Network of Gynaecological Cancer Advocacy Groups. The document is divided into six sections: (1) general recommendations; (2) image-guided biopsy (imaging guidance, sampling methods); (3) indications and contraindications; (4) technique; (5) reporting; and (6) training and quality assurance. To ensure that the statements are evidence-based, the current literature was reviewed and critically appraised. Preliminary statements were drafted based on this review of the literature. During a conference call, the whole group discussed each preliminary statement, and a first round of voting was carried out. The group achieved consensus on all 46 preliminary statements without the need for revision.  These ISUOG/ESGO statements on ultrasound-guided biopsy in gynecological oncology, together with a summary of the evidence supporting each statement, are presented. This Consensus Statement is supplemented by detailed narrated videoclips presenting different approaches and indications for ultrasound-guided biopsy, a patient leaflet, and an extended version which is presented herein and includes a detailed review of the evidence. |
| **Key Words**: Ultrasound-guided biopsy, Gynecologic oncology, Consensus Statement |

Affiliations: ^1^Department of Gynecology, Obstetrics and Neonatology, First Faculty of Medicine, Charles University and General University Hospital in Prague, Prague (Czech Republic); ^2^Institut Bergonie, Bordeaux (France); ^3^University of Navarra, Pamplona (Spain); ^4^Hospital QuirónSalud, Málaga (Spain); ^5^Department of Pathology, First Faculty of Medicine, Charles University and General University Hospital in Prague, Prague (Czech Republic); ^6^Department of Clinical Science and Education, Karolinska Institutet, Södersjukhuset, Stockholm (Sweden); ^7^iNOVA4Health, NOVA Medical School, Faculdade de Ciências Médicas, NMS, FCM, Universidade NOVA de Lisboa; Lisbon (Portugal); ^8^Instituto Portugues de Oncologia de Lisboa Francisco Gentil, Lisboa (Portugal); ^9^Unità Operativa di Chirurgia dei Organi Genitali Esterni Femminili, Divisione di Ginecologia Oncologica, Dipartimento Scienze della Salute della Donna, del Bambino e di Sanità Pubblica, Fondazione Policlinico Universitario A. Gemelli IRCCS, Rome (Italy); ^10^Gemelli Women Health Center for Digital and Personalized Medicine, Dipartimento Scienze della Vita e Sanità Pubblica, Università Cattolica del Sacro Cuore, Rome (Italy); ^11^Mohn Medical Imaging and Visualization Centre (MMIV), Department of Radiology, Haukeland University Hospital, Bergen (Norway); ^12^Section for Radiology, Department of Clinical Medicine, University of Bergen, Bergen (Norway); ^13^EGA Institute for Women's Health, University College London, London (UK); ^14^Department of Gynaecology, National Institute of Oncology, Budapest (Hungary); ^15^Doctoral School of Clinical Medicine, University of Szeged, Szeged (Hungary); ^16^Gynecologic Oncology Service, Nairi Medical Center, National Institute of Health, Yerevan, Armenia; ^17^Department of Gynecological Oncology, Pomeranian Hospitals (Szpitale Pomorskie), Gdynia (Poland); ^18^Clinic of Surgical Oncology, Faculty of Health Sciences with the Institute of Maritime and Tropical Medicine, Medical University of Gdansk, Gdansk (Poland); ^19^Department of Development and Regeneration, KU Leuven, Leuven (Belgium); ^20^Department of Cardiovascular Diseases, University Hospitals Leuven, Leuven (Belgium); ^21^Department of Gynaecological Oncology, KK Women’s and Children’s Hospital, Singapore (Singapore); ^22^Academic Unit of Obstetrics and Gynecology, Hospital Polyclinic San Martino and University of Genoa, Genoa (Italy).

Address correspondence and reprint requests to Daniela Fischerova, MD, PhD. Department of Gynecology, Obstetrics and Neonatology, Kateřinská 32, 121 08 Prague, Czech Republic.

Email: [Daniela.Fischerova@vfn.cz](mailto:Daniela.Fischerova@vfn.cz)

The decision to develop this Consensus Statement was made jointly by ISUOG and ESGO. ISUOG and ESGO are non-profit knowledgeable societies.

The development group (including all authors) is collectively responsible for the decision to submit for publication. D. Fischerova (chair), U. Scovazzi and F. Planchamp (methodologist) have written the first draft of the manuscript. All other contributors have actively given personal input, reviewed the manuscript, and have given final approval before submission.

1. **INTRODUCTION**

There has been increasing use of minimally invasive diagnostic procedures in gynecologic oncology in recent years, which confers the advantage of accurate diagnosis while minimizing procedure-associated morbidity[^1-3^](#_ENREF_1). These diagnostic procedures are often indispensable to inform patient management in situations such as unresectable advanced gynecological cancer or suspected disease recurrence. There are no specific guidelines available to assist gynecologists in performing ultrasound-guided biopsies. Real-time biopsy guidance using a transvaginal or transrectal ultrasound approach allows close proximity to gynecological tumors, ensuring high diagnostic adequacy and accuracy, while the low risk of procedure-related complications enables the biopsy to be performed in the outpatient setting. In addition, avoiding patient referral to other physicians such as interventional radiologists can reduce healthcare costs, limit patient distress and shorten the time to initiation of appropriate treatment.

In parallel with the introduction and improvement of ultrasound diagnosis in gynecological oncology, a broad spectrum of minimally invasive ultrasound-guided diagnostic and therapeutic procedures has been developed (Table 1).

**Table 1** Image-guided procedures in gynecological oncology

| Procedure | Type of intervention |
| --- | --- |
| Diagnostic | Fine-needle aspiration  Core-needle (tru-cut) biopsy |
| Diagnostic/Therapeutic | Drainage of fluid collections*  Paracentesis  Thoracocentesis |
| Palliative | Insertion of permanent peritoneal or thoracic catheter |

*Fluid collections can occur in different clinical scenarios, including abscess, lymphocele, peritoneal pseudocyst, seroma and hematometra following trachelectomy or brachytherapy.

Ultrasound-guided diagnostic procedures include core-needle (tru-cut) biopsy and fine-needle aspiration. Any ultrasound-guided intervention changes the ultrasound modality from a risk-free method to an intervention that carries risk and therefore needs its own clear standard operating procedure. Given an appropriate indication and careful execution, these procedures are well-tolerated by patients and less risky and costly than surgical procedures[^4-11^](#_ENREF_4). Regarding surgical risk, the largest analysis to date showed the 10-year experience with laparoscopy in Memorial Sloan-Kettering Cancer Centre[^6^](#_ENREF_6). Diagnostic laparoscopies performed to rule out malignancy in the peritoneal cavity showed an overall complication rate of 15%, ranging from grade 1 (mild) to grade 5 (death). There was a 5% major (grade 3 to 5) complication rate, including 3 deaths, which occurred due (1) enterotomy following the initial peritoneal access with peritonitis and sepsis, (2) pulmonary edema and progressive respiratory failure, (3) pulmonary embolus[^6^](#_ENREF_6). In contrast, both core-needle biopsy and fine-needle aspiration are associated with an overall major complication rate of <1.5% in gynecologic oncology[^7^](#_ENREF_7)^,^[^12-15^](#_ENREF_12). A meta-analysis of comparative studies reporting complications demonstrated a significantly lower risk of complications associated with core-needle biopsy than incisional biopsy for soft tissue sarcoma (RR, 0.14)[^16^](#_ENREF_16). Regarding costs, Goranova *et al.* reported an average overall cost to perform an image-guided biopsy of £800 ($1000, €950), compared to a supposedly higher cost of a laparoscopic surgery for diagnostic or staging purposes (variable from around $1500 to $9400 depending on indication and country)[^8-11^](#_ENREF_8)^,^[^17^](#_ENREF_17)^,^[^18^](#_ENREF_18).

Until recently, common practice has been for ultrasound-guided procedures to be performed mainly by interventional radiologists. There have been many useful guidelines published on the topic of interventional ultrasound using the percutaneous ultrasound-guided approach. However, these guidelines focusing on intraabdominal interventions (including biopsies of, for example, liver, kidney, pancreas, spleen) do not address the pelvic organs fully[^19-23^](#_ENREF_19). Moreover, the transvaginal approach is rarely included in the armamentarium of radiologists. Scientific data from radiologic departments suggest underestimation of the applicability of transvaginal ultrasound-guided biopsy in a gynecological oncology setting, with limited case numbers over a long period of time[^24^](#_ENREF_24).

The lack of information on best practice in ultrasound-guided biopsy in gynecology and the growing need for this service in every gynecological oncology center led to the initiation of the process that produced this Consensus Statement. The objective of this work is to assist clinicians, including gynecological sonographers, gynecological oncologists and radiologists, to achieve the best standards of practice in diagnostic (biopsy) procedures. It includes the following sections:

1. General recommendations
2. Image-guided biopsy (imaging guidance modalities, adequacy, accuracy, diagnostic yield and complications of sampling methods)
3. Indications and contraindications

4. Technique

5. Reporting

6. Training and quality assurance

A comprehensive summary of published data for all six sections is provided in this extended version of the Consensus Statement (Appendix S1). The technique is demonstrated in Videoclips S1–S3 and the indications for biopsy in Videoclip S4. A sample patient leaflet is also provided to aid counseling and communication with patients (Appendix S2). The literature search in Medline is presented in Appendix S3. Levels of evidence and grades of statements used in this Consensus Statement are presented in Appendix S4.

1. **RESPONSIBILITIES**

The present series of statements represent a consensus of the authors regarding their currently accepted approaches for ultrasound-guided biopsy, based on the available literature and evidence. Any clinician applying or consulting these statements is expected to use independent medical judgment in the context of individual clinical circumstances to determine all patients’ care and treatment. These statements are presented without any warranty regarding their content, use or application and the authors disclaim any responsibility for their application or use in any way.

1. **METHODS**

These consensus statements on ultrasound-guided biopsy in gynecological oncology were developed using an eight-step process, chaired by Professor Daniela Fischerova (Figure S1).

**Nomination of a multidisciplinary international group**

**Identification of scientific evidence**

**Formulation of preliminary consensus statements**

**Discussion of each preliminary consensus statement**

**1^st^ round of voting**

**Revision of the consensus statements** *(where necessary)*

**2^nd^ round of voting**

**Finalization of the consensus statements**

**Figure S1.** Development process

Aiming to assemble a multidisciplinary international group, the International Society of Ultrasound in Obstetrics and Gynecology (ISUOG) and the European Society of Gynaecological Oncology (ESGO) nominated 16 experts who have demonstrated leadership in the use of ultrasound-guided biopsy in the clinical management of patients through research, administrative responsibilities and/or committee membership. Altogether, six gynecologists with special interest in ultrasonography, one radiologist, two pathologists, one cardiologist and six gynecological oncologists were included. In addition, two early-career gynecological fellows with special interest in ultrasound-guided biopsy were nominated to participate from the European Network of Young Gynae Oncologists (ENYGO) within ESGO and from ISUOG, to form the final working group of 18 participants. The participants did not represent the societies from which they were elected but were asked to base their decisions on their own experience and expertise. A patient representative from the European Network of Gynaecological Cancer Advocacy Groups (ENGAGE Co-Chair) was also included in the group.

An initial conference call including the whole group was held to facilitate introductions, as well as to review the purpose and scope of the Consensus Statement. The proposed document was divided into six sections, each with a lead author and a working group according to previously expressed interests and expertise. To ensure that the statements were evidence-based, the current literature was reviewed and critically appraised. A systematic literature review of relevant studies published between technique inception and 2023 was carried out using the MEDLINE database (Appendix S3). The literature search was limited to publications in the English language. Priority was given to high-quality systematic reviews, meta-analyses and validating cohort studies, although studies with lower levels of evidence were also evaluated. The search strategy excluded editorials, letters and case reports. The reference list of each identified article was reviewed for other potentially relevant articles. The results of the literature search were distributed to the whole group, including electronic full-text versions of each article. One of the authors (F.P.) provided methodology support throughout the process, but did not participate in the voting on the consensus statements. Thus, there were 18 voting participants.

Each lead author, following discussion with his/her working group, was responsible for drafting preliminary statements after a review of the relevant literature. These were then circulated to the entire group prior to a second conference call. During the second conference call, the whole group discussed each preliminary statement, and a first round of binary voting (agree/disagree) was carried out for each potential statement. All 18 participants took part in each vote, but they were permitted to abstain from voting if they felt they had insufficient expertise to agree/disagree with the statement or if they had a conflict of interest that could influence their vote. Statements would be removed if a consensus among group members was not reached. The voters had the opportunity to provide comments or suggestions together with their votes, which would require revision of the statement and a second round of voting. The group achieved consensus on all 46 preliminary statements without the need for revision and another round of voting. Thus, based on the results of the first round of voting, the statements were finalized. In the main text of this Consensus Statement, we present a summary of the supporting evidence, the finalized series of statements, and their levels of evidence and grades as described in Appendix S4. The extended version of this Consensus Statement, including a detailed evidence review, is presented herein (Appendix S1). The short version of this Consensus Statement, together with a summary of the evidence supporting each statement, is presented in the print version of this article.

1. **RESULTS**

## General recommendations

The purpose of performing minimally invasive biopsy procedures in gynecology is to obtain a representative tissue sample from suspected pathological processes to enable a morphological examination[^11-13^](#_ENREF_11)^,^[^24^](#_ENREF_24)^,^[^25^](#_ENREF_25). These biopsy procedures are particularly useful for patients not eligible for more invasive interventions or to accelerate the process from diagnosis to therapy without the need for a period of recovery[^7^](#_ENREF_7)^,^[^12^](#_ENREF_12). In addition, in the era of personalized medicine, obtaining biopsy samples from gynecological cancers, either primary or recurrent disease, has become increasingly important to obtain material for predictive testing and to plan subsequent targeted therapy[^5^](#_ENREF_5)^,^[^11^](#_ENREF_11)^,^[^26^](#_ENREF_26)^,^[^27^](#_ENREF_27). Evidence indicates that repeated biopsies at different times are well tolerated by patients[^4^](#_ENREF_4)^,^[^5^](#_ENREF_5)^,^[^28^](#_ENREF_28). The Cancer Genome Atlas (TCGA) project has led to the identification of the molecular aberrations of some of the most frequent gynecological malignancies[^1-3^](#_ENREF_1). The aim of such molecular studies is to improve patient outcomes by aiding in the development of more targeted therapies[^1-3^](#_ENREF_1). Using the molecular profile of gynecological cancers, potentially at multiple time points in the same patient, has enabled the predicting of the treatment response throughout the entire course of the disease[^29^](#_ENREF_29)^,^[^30^](#_ENREF_30). This has been highlighted in recent clinical trials, including on ovarian cancer, in which the treatment of recurrent tumor was based on consecutive samples to enable a “molecular-match therapy”[^26^](#_ENREF_26)^,^[^31-34^](#_ENREF_31).

The indication for biopsy, performance and reporting of biopsy, and final interpretation of results according to the clinical and imaging findings require multidisciplinary team expertise. This was recently highlighted by a National Cancer Institute panel, which reported that improving the communication between radiologists, oncologists and pathologists increases the probability of obtaining fit-for-purpose samples for pathological diagnosis and/or genomic analysis[^35^](#_ENREF_35). Multidisciplinary team discussion is also required if procedure-related risk is deemed to be high or in the event of inconclusive biopsy results to discuss possible alternative diagnostic options[^36^](#_ENREF_36).

**Statement 1**: Image-guided tumor biopsy forms an integral component of the individualized treatment of gynecological cancers, especially in the context of disseminated disease, recurrence or the presence of surgical contraindications.

- Level of evidence: 3b
- Grade of statement: C
- Consensus: yes, 100% (*n* = 18); no, 0% (*n* = 0); abstain, 0% (*n* = 0)

**Statement 2**: Expertise and effective communication within a multidisciplinary team are essential to determine a proper indication and technical execution of an ultrasound-guided biopsy, to maximize the safety of the procedure, the adequacy of the specimen and the accuracy of pathology reporting, and to optimize the integration of biopsy results with clinical and imaging findings.

- Level of evidence: 3b
- Grade of statement: C
- Consensus: yes, 100% (*n* = 18); no, 0% (*n* = 0); abstain, 0% (*n* = 0)

## Image-guided biopsy (image-guidance modalities, adequacy, accuracy, diagnostic yield and complications of sampling methods)

Image-guided biopsy aims to sample a target tissue using a guided approach, by ultrasound or other imaging technology. Real-time biopsy guidance is essential to optimize tissue sample acquisition. Obtaining biopsy specimens which are suitable for histopathological and molecular analysis should be quick and minimally invasive, and should pose a low risk of procedure-related complications. Ideally the procedures should be performed in the outpatient setting, to avoid delays in initiating appropriate treatment, while also ensuring diagnostic accuracy and safety[^12^](#_ENREF_12)^,^[^13^](#_ENREF_13)^,^[^37^](#_ENREF_37).

In gynecology, there are two biopsy techniques, either using a cutting needle providing a core of tissue (core-needle biopsy, also called tru-cut biopsy) or tumor cells aspiration (fine-needle aspiration). Core-needle biopsy provides tissue samples suitable for histologic, immunohistochemical, genetic, multi-omic or immunologic examination, while fine-needle aspiration yields cells or small tissue fragments that are sufficient for cytologic examination and may sometimes also be used for some complementary studies if the material is quantitatively sufficient. In recent years, other techniques, and technologies, such as "liquid biopsy", have been introduced into clinical practice to tailor treatment strategies for certain tumors, such as lung cancer but these are outside the scope of this consensus statement on ultrasound-guided biopsy[^35^](#_ENREF_35)^,^[^38^](#_ENREF_38).

- - 1. Image-guidance modalities

To guarantee a high adequacy, accuracy, safety and a high diagnostic yield of the procedure, the biopsy is guided by imaging such as ultrasound (US), computed tomography (CT) and magnetic resonance imaging (MRI) or fusion imaging (Table S1).

**Table S1.** Comparison of image-guidance modalities in current practice in interventional procedures in gynecology

| *Variables* | *Image-guidance modality* | | |
| --- | --- | --- | --- |
|  | **Ultrasound** | **MRI** | **CT** |
| Costs | 1x | 4x | 2x |
| Availability | Specialized centers | Specialized centers | Specialized centers |
| Range of examination | Abdomen and pelvis, peripheral lymph nodes, superficial soft tissues | Whole body | Whole body |
| Duration of imaging-guided procedure (minutes) | 5-15 | 20-70 | 20-45 |
| Dynamic evaluation* | Real time control of the needle with multiplanar evaluation | Lack of real time feedback | Lack of real time feedback |
| Preparation before imaging | None  Consider fasting (4 hours) prior to high bleeding risk procedure | Anti-peristaltic agents  Consider fasting (4 hours) prior to high bleeding risk procedure | None  Consider fasting (4 hours) prior to high bleeding risk procedure |
| Contrast agent | None, optionally microbubble bolus (SonoVue) | Optional, gadolinium-based | Optional, iodine-based |
| Radiation exposure | None | None | 10-20 mSv |
| Procedural limitation | Limited acoustic window, obesity | Claustrophobia, MR incompatible implants (including cochlear implants, some implantable cardioverter-defibrillators or pacemakers, electrodes), metal foreign bodies (intracranial or intraorbital) | Contraindication for iodine-based contrast agent if:  - severe renal insufficiency  - untreated hyperthyroidism  - severe allergy to iodine contrast agent |
| Dependence on expertise | Yes | Yes | Yes |

*Ultrasound imaging can provide information about the site-specific perfusion to detect the most vital part of the tumor to be sampled; moreover, it is able to assess the absence of immediate complications such as bleeding (“fountain sign”). ^&^In patients with renal insufficiency gadolinium contrast media must be used with caution. CT, computed tomography; MRI, magnetic resonance imaging; mSv, millisievert. The radiation dose of 10-20 mSv during CECT (thorax, abdomen, pelvis) is equivalent to about 3-7 years of background radiation.

Ultrasound-guided biopsy requires ultrasound expertise and good anatomical knowledge of the pelvis, abdomen, groins, axilla, and other locations[^12^](#_ENREF_12)^,^[^37^](#_ENREF_37)^,^[^39-44^](#_ENREF_39). Ultrasound offers many benefits, including a low rate of false-negative biopsies and low rate of complications, wide availability, short procedure time, lack of ionizing radiation, portability and relatively low cost (Table S1)[^45^](#_ENREF_45). Crucially, it allows the real-time intra-procedural visualization of the biopsy needle and target lesion, dynamic multiplanar vision (i.e. the ability to guide the procedure in almost any anatomical plane), high soft tissue resolution (especially in the pelvis in the case of endovaginal/endoanal probe insertion) and the use of power or color Doppler, which are essential to achieve safe access to target lesions[^12^](#_ENREF_12)^,^[^37^](#_ENREF_37)^,^[^39^](#_ENREF_39)^,^[^40^](#_ENREF_40)^,^[^42^](#_ENREF_42)^,^[^45^](#_ENREF_45). Doppler examination helps to visualize blood vessels and define the most suitable part of the tumor for biopsy. Ultrasound-guided procedures can be done by utilizing a variety of transducers (endocavitary, convex array, linear array and sector, among others) allowing different approaches for biopsy-needle insertion (percutaneous, transvaginal, transcervical, transrectal) (Figure 1, Videoclip S1-3)[^12^](#_ENREF_12)^,^[^37^](#_ENREF_37)^,^[^40^](#_ENREF_40).


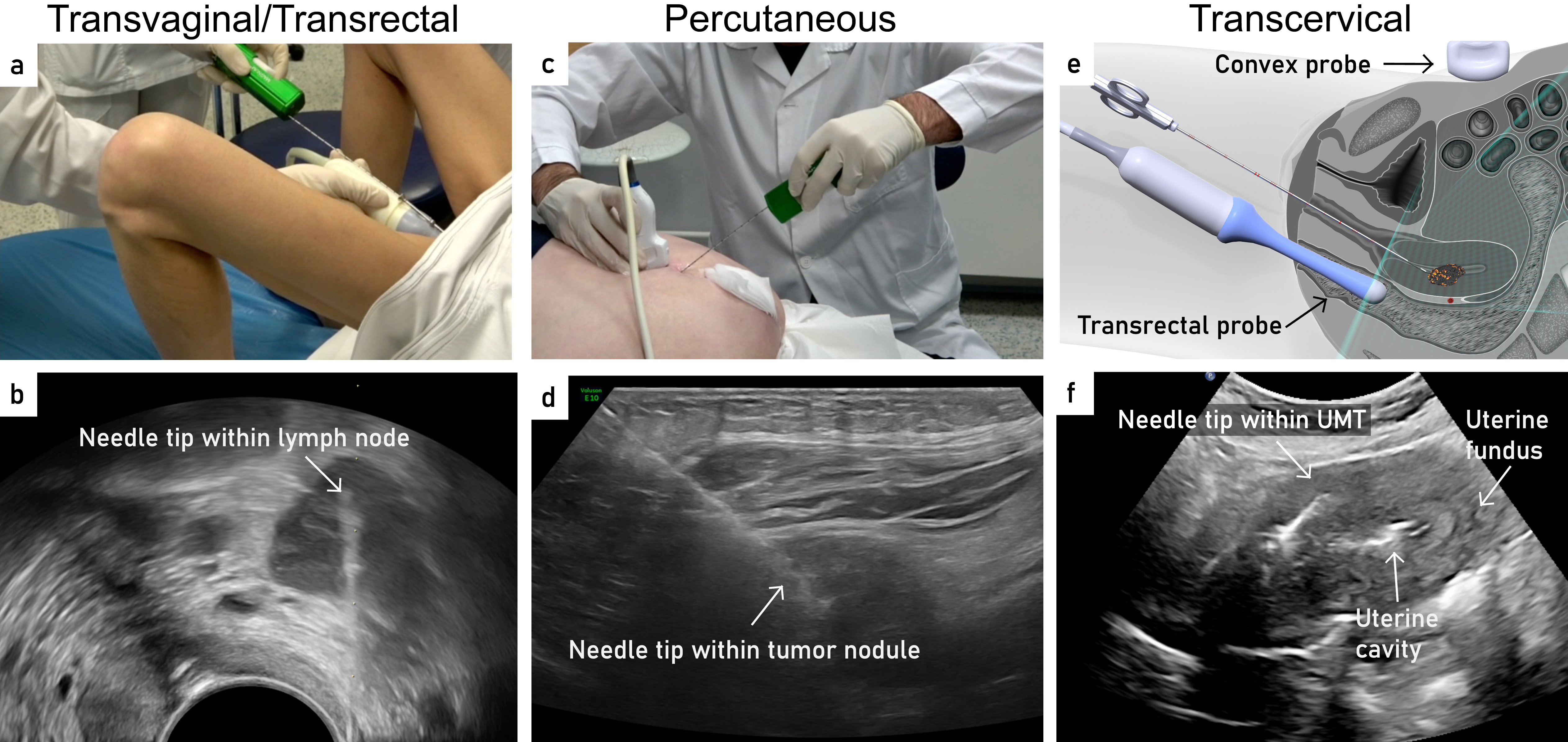


**Figure 1** Illustration of different approaches for performing core-needle biopsy. (a,b) Transvaginal or transrectal approach: the biopsy device with biopsy needle is inserted into a metal needle guide attached to the endocavitary probe (a); the biopsy is taken from an infiltrated pelvic parietal (iliac) lymph node (b). (c,d) Percutaneous approach, using the free-hand technique: the biopsy needle is inserted along the longitudinal axis of the probe, guided by the ultrasound beam (c); the biopsy is taken from the infiltrated abdominal wall (Sister Mary Joseph’s nodule) (d). (e,f) Transcervical approach: using an ultrasound probe placed in the rectum or on the abdomen for guidance (e), the biopsy needle is visible approaching the lesion transcervically and transcavitarily (in-organ biopsy) (f). UMT, uterine mesenchymal tumor. See also Videoclips S1–S3.

The safest path to the target lesion should be selected avoiding puncturing of non-target organs and blood vessels. **The transvaginal or transrectal approach,** using a disposable needle guide attached to the endocavitary probe, usually has no major limitations in obtaining biopsies from a pelvic lesion. An exception could be if the needle path, which corresponds to the path of the ultrasound beam, cannot be angled towards the target. In such instances, greater flexibility can be obtained by changing the probe pressure and angling within the vagina/rectum. Longer needles are also needed when using the endoluminal probe. Specific advantages of the transvaginal and transrectal approaches include a significantly shorter distance to target when compared to the percutaneous approach[^24^](#_ENREF_24). All these enable performing the procedure in women with obesity or various anatomical differences with high precision and an excellent safety profile. For biopsy of pelvic lesions, the transvaginal approach is preferred for its good diagnostic yield and safety profile and should be considered the first choice, even when other approaches are feasible[^24^](#_ENREF_24)^,^[^46^](#_ENREF_46). Within the pelvis, the transrectal biopsy approach offers a short distance to the target and visualization similar to that of the transvaginal approach, but is less comfortable for patients and carries the potential risk of bacterial contamination[^47-50^](#_ENREF_47). It is crucial to explain carefully the reasons for recommending a transrectal biopsy approach and to obtain the patient’s explicit consent before this approach is used.

The **transcervical approach** (ultrasound-guided transuterine cavity core-needle biopsy) can be applied to patients with suspected uterine (e.g. mesenchymal) tumors[^51^](#_ENREF_51). In the transcervical biopsy approach, for ultrasound guidance, the probe is placed in the rectum or on the abdomen, while the biopsy needle is inserted transcervically through the endocervical canal and advanced through the uterine cavity (transcavitary). This approach is allowing the use of a longer transcervical needle to reach suspected (more distant) uterine tumors without passing the needle through the uterine serosa[^51-53^](#_ENREF_51). It is considered an “in-organ” biopsy, which is important if sarcoma is suspected to minimize the risk of spread of malignant tumor cells or tumor seeding along the needle biopsy tract caused by the performance of the biopsy[^54^](#_ENREF_54). Transcervical core-needle biopsy has specific indications and is a procedure which requires more periprocedural preparation than transvaginal, transrectal or percutaneous approaches.

The adequacy of biopsy using a **percutaneous approach**, with or without a needle guide, is highly dependent on adequate acoustic conditions and the location of the target lesion. The percutaneous approach includes all situations where the needle puncture is through the skin. It includes the transabdominal approach for biopsy of abdominal organs and the transcutaneous approach for biopsy of soft tissues (such or perineum or breast) or for biopsy of peripheral lymph nodes (groins, axilla, etc.)[^37^](#_ENREF_37)^,^[^55^](#_ENREF_55). The main limitations of percutaneous ultrasound guidance are related to the possible difficulty in visualizing the tumor target or needle tip. This may occur when the distance between the target and the ultrasound probe is large, such as: in obese subjects or in the presence of high-volume ascites; in case of acoustic shadowing due to intestinal air or solid tissue such as bone or calcified areas; or when the lesion is inaccessibile due to critical or vulnerable anatomical structures[^7^](#_ENREF_7)^,^[^24^](#_ENREF_24). Regarding difficult-to-access tumor sites, freehand biopsy (i.e. without needle guide) allows more flexibility in guiding the needle to the target but needs experience and is more time consuming[^56^](#_ENREF_56). If the large distance of the target from the point of entry of the biopsy needle is related to high volume ascites, puncture and drainage of the ascites is recommended before the biopsy. Compression of the ultrasound transducer on the abdominal wall can also significantly decrease skin-to-target distance by up to 40% in abdomino-pelvic targets[^57^](#_ENREF_57).

When visualization at ultrasound examination is problematic, contrast-enhanced ultrasound, or coregistration (fusion imaging) of real-time ultrasound with acquired images from CT, MRI, positron emission tomography (PET), may be considered, although these require specialized software and equipment and are less widely used[^26^](#_ENREF_26)^,^[^58-60^](#_ENREF_58). **Contrast enhanced ultrasound** relies on the injection of contrast agent which improves signal to noise ratio created by gas in the form of microbubbles/nanobubbles[^61^](#_ENREF_61). To enhance diagnostic yield of biopsy, it has emerged as a reliable technique to evaluate the presence of vascularity as a sign of tumor tissue viability, especially in large intra-abdominal tumors with areas of necrosis[^26^](#_ENREF_26)^,^[^62^](#_ENREF_62)^,^[^63^](#_ENREF_63). The use of **fusion imaging** can overcome some limitations in target localization and biopsy guidance. The use of image fusion software and virtual navigation systems involves the fusion of real-time ultrasound with a previously recorded data set from CT, MRI or PET in combination with CT (PET/CT)[^58-60^](#_ENREF_58). After alignment of landmarks between ultrasound and CT, MRI, or PET/CT images, joint navigation of cross-sectional images is achieved, with the advantage of the real-time nature of ultrasound combined with additional information from the functional imaging. Fusion imaging is already widely used in urology to guide prostatic biopsies, it has been applied in biopsy of liver target lesion/-s with suspicious uptake on MRI and PET/CT, but also in gynecological malignancies in cases such as the precision biopsy of superficial lymph nodes[^58-60^](#_ENREF_58)^,^[^64^](#_ENREF_64).

Regarding **other image-guidance modalities**, the next most commonly used imaging method after ultrasound is CT, which is an efficient and safe technique for guiding biopsies from retroperitoneal and abdominal masses, especially in locations with limited acoustic windows for sonographic assessment[^40^](#_ENREF_40)^,^[^65-69^](#_ENREF_65). It is a safe procedure with good diagnostic performance (accuracy estimates 82-100%), but with major limitations related to the low tissue contrast, the need for patient fasting, the exposure to radiation and the risk of contrast-agent related toxicity[^70^](#_ENREF_70)^,^[^71^](#_ENREF_71). Another limitation may be for deep pelvic lesions since the CT-guided percutaneous approach requires a longer biopsy route when compared to the transvaginal/transrectal route (mean 8.6 cm vs 1.1 cm), leading to more non-diagnostic results and a risk of complications[^24^](#_ENREF_24). MRI guidance, despite providing good soft tissue resolution and having no risks associated with radiation exposure, is still used only rarely, as it requires special non-magnetic equipment and experienced operators[^72^](#_ENREF_72). PET in combination with CT or MRI has also been proposed as guidance for biopsy; however, this is rarely used in gynecological practice[^73^](#_ENREF_73)^,^[^74^](#_ENREF_74). Compared to ultrasound, CT, and MRI are more expensive and less readily available techniques (Table S1)[^45^](#_ENREF_45).

Ultrasound-guided core-needle biopsy is quicker and easier to use and thus accelerates the diagnostic process. It is possible to perform ultrasound-guided biopsy in real time, simultaneously with the diagnosis. Data on the comparison of ultrasound versus other imaging modalities as guidance for core-needle biopsy are limited and no randomized controlled trials are available. However, Hewitt et al. reported ultrasound to be as effective as CT in guiding biopsies for peritoneal carcinomatosis[^40^](#_ENREF_40). Another study by Griffin et al. reported a similar accuracy for core-needle biopsy performed under either ultrasound or CT as guidance (89% vs 84% respectively)[^65^](#_ENREF_65). In clinical practice, there is enough evidence demonstrating that the transvaginal or transrectal ultrasound approach is preferable to percutaneous CT navigation in obtaining a biopsy of deep pelvic lesions[^24^](#_ENREF_24)^,^[^75-77^](#_ENREF_75). Regarding percutaneous biopsy of intra-abdominal organs, ultrasound guidance is more frequently used than CT guidance for navigation during biopsy with a low rate of complications[^39^](#_ENREF_39). In a study by Atwell et al., liver biopsy under CT navigation was performed in only 157 of 3636 liver lesions (4.3%) with two bleeding complications (1.3%), compared to 3479 liver biopsies under ultrasound navigation with 15 bleeding complications (0.4%)[^78^](#_ENREF_78). Of 5832 renal biopsies, 110 (1.9%) were performed under CT guidance with a 0.9% rate of bleeding complications, while the remaining 5722 renal biopsies were performed under ultrasound guidance with 0.7% having bleeding complications[^78^](#_ENREF_78). Regarding patient satisfaction with each method, a prospective multicentric diagnostic accuracy study demonstrated that ultrasound was the imaging method preferred by most patients despite being more painful (mainly due to endovaginal probe insertion) compared to CT and whole-body diffusion weighted MRI[^79^](#_ENREF_79). Whole-body diffusion weighted MRI was preferred by the fewest number of patients[^79^](#_ENREF_79).

**Statement 3**: Image-guided biopsy is a minimally invasive technique, which provides a safer alternative to surgery. It is effective in obtaining an adequate tissue sample for an actionable pathological result which can be used to guide treatment.

- Level of evidence: 3a
- Grade of statement: B
- Consensus: yes, 94% (*n* = 17); no, 0% (*n* = 0); abstain, 6% (*n* = 1)

**Statement 4**: Among all imaging methods, ultrasound should be considered the first choice for guidance of biopsy, because it provides real-time imaging, is versatile and allows a multiplanar view.

- Level of evidence: 3b
- Grade of statement: B
- Consensus: yes, 94% (*n* = 17); no, 0% (*n* = 0); abstain, 6% (*n* = 1)

**Statement 5**: Ultrasound-guided biopsy can be performed with different approaches (transvaginal, transcervical, transrectal and percutaneous), using different transducers (endocavitary, convex array and linear array) according to the safest path to the target and its best visualization.

- Level of evidence: 4
- Grade of statement: B
- Consensus: yes, 100% (*n* = 18);  no, 0% (*n* = 0); abstain, 0% (*n* = 0)

**Statement 6**: Doppler examination may help to define the most suitable viable part of the tumor for biopsy. Alternatively, contrast-enhanced ultrasound can be used.

- Level of evidence: 4
- Grade of statement: C
- Consensus: yes, 94% (*n* = 17); no, 0% (*n* = 0); abstain, 6% (*n* = 1)

**Statement 7**: In poorly visualized targets or when there are inconclusive findings with B-mode ultrasound, alternative imaging techniques to guide the biopsy, such as contrast-enhanced ultrasound or novel image fusion methods, can be employed.

- Level of evidence: 3a
- Grade of statement: C
- Consensus: yes, 78% (*n* = 14); no, 0% (*n* = 0); abstain, 22% (*n*= 4)

**Statement 8**: Other imaging guidance (CT, MRI or PET/CT) may be chosen according to lesion accessibility and/or to overcome suboptimal acoustic conditions.

- Level of evidence: 4
- Grade of statement: C
- Consensus: yes, 100% (*n* = 18); no, 0% (*n* = 0); abstain, 0% (*n* = 0)
  - 1. Biopsy methods

Two main sampling techniques are commonly used in gynecological oncology practice, applied to different cases according to the location of the target lesion, the type of lesion and its solid/fluid components, the clinical condition of the patient and other factors[^55^](#_ENREF_55)^,^[^80^](#_ENREF_80). The first, core-needle biopsy (also known as tru-cut biopsy), uses a side-cutting or end-cutting needle to provide tissue samples suitable for histological analysis and immunochemistry (Figure 2).


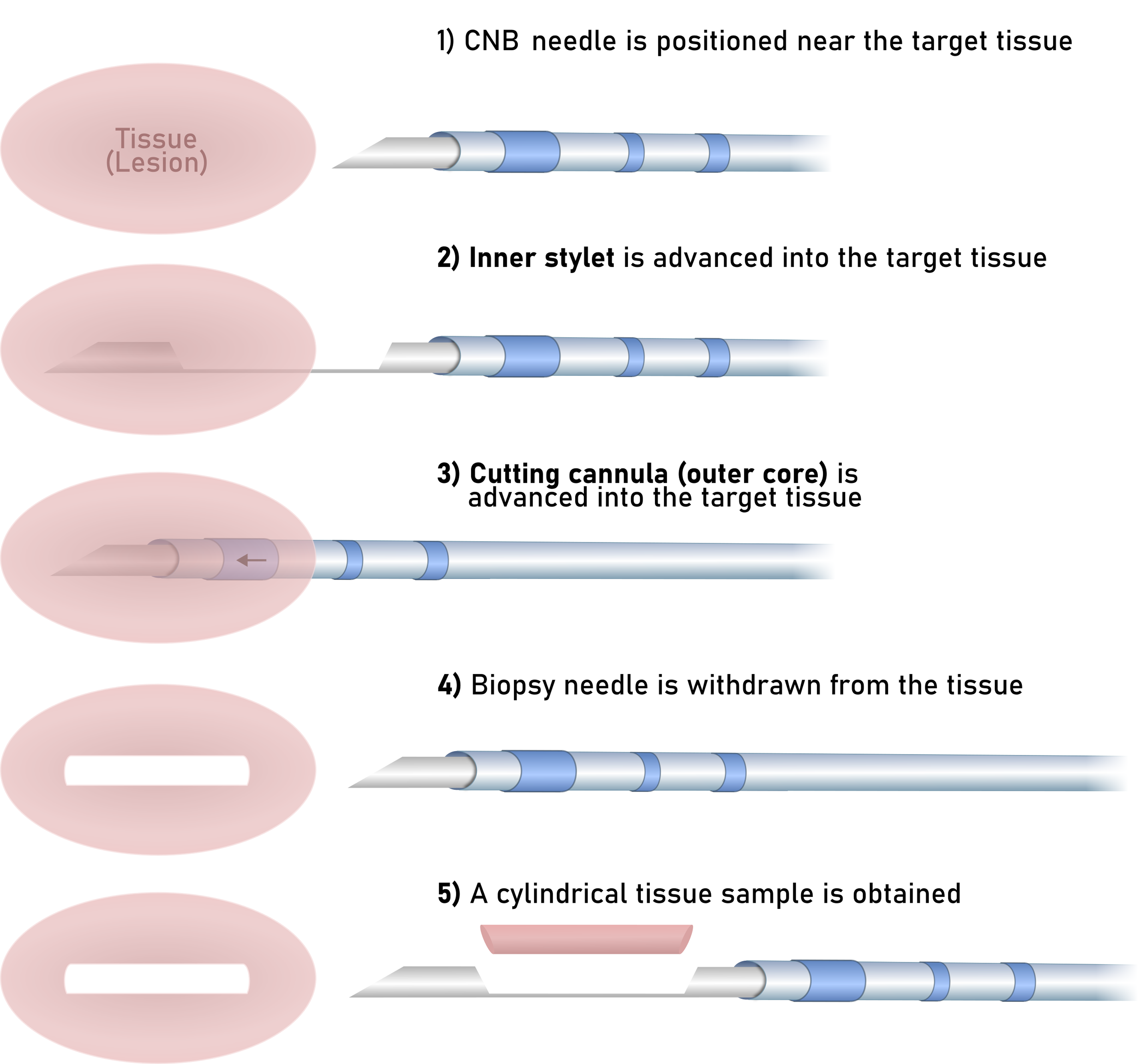


**Figure 2** Illustration of core-needle biopsy (CNB) mechanism, with a side-cutting needle used to obtain biopsy sample. The tip of the biopsy needle should be positioned at the edge of or inside the lesion before firing, depending on the size of the lesion and the location of viable area(s) of tumor identified.

The second, fine-needle aspiration, also commonly referred to as fine-needle aspiration cytology or biopsy, yields cells or, rarely, small tissue fragments, which are sufficient for cytological examination and may sometimes also be used for complementary studies if there is enough material.

The use of biopsy specimens can also be extended to other clinical and research applications, such as molecular testing (e.g. complex genomic profiling by next-generation sequencing) and other analyses (e.g. assessment of stromal microenvironment) (Table 2).

**Table 2** Comparison between fine-needle aspiration and core-needle biopsy

|  | *Fine-needle aspiration* | *Core-needle biopsy* |
| --- | --- | --- |
| Needle size | 20–25 G (outer diameter from 0.9 mm to 0.5 mm)* | 14–18 G (outer diameter from 2.1 mm to 1.3 mm) |
| Sample collection | Aspiration needle, often connected to a syringe whose plunger can be used to apply negative pressure to aspirate the specimen | Hollow needle with a spring-loaded cutting action, integrated in an automated or semi-automated gun |
| Sample type | Aspiration of cellular material or fluid from a lesion or effusion | Cylindrical/semicylindrical core of tissue from a solid mass |
| Material preservation/pro-cessing | The collected material is usually expelled onto multiple glass slides, creating smears, or into a container with a fixative, such as formalin; alternatively, medium for liquid-based cytology can be used | The cylindrical/semicylindrical core of tissue from a solid mass is typically preserved in a fixative solution, most commonly formalin, and after fixation, the tissue core is processed to embed it in paraffin |
| Staining | The glass slides are typically stained with various specialized dyes (May-Grünwald Giemsa stain; Papanicolaou stain) or H&E | The glass slides are typically stained with H&E; immunohistochemistry is commonly used |
| Microscopic examination | On microscopy, the pathologist evaluates cell morphology, structure and other characteristics, to reach a diagnosis | On microscopy, the pathologist assesses tissue architecture, cell morphology and other characteristics; this detailed examination aids in making a diagnosis |
| Advantages | Possibility of multiple passes in various directions for each sample  Flexibility in specimen preparation  Collection of fresh and intact cells  Lower complication rate  Low level of pain and rare need for local anesthesia  Less expensive | Larger intact tissue sample with preserved architecture  Tissue for immunohistochemistry and ancillary studies (e.g. NGS)  Higher yield for fibrotic tissue lesions  For most lesions, higher sensitivity, specificity and accuracy than fine-needle aspiration to make a definitive diagnosis |
| Limitations | Limited tissue architecture  Lower yield for fibrotic tissue lesions  Difficult on cytological smears to distinguish e.g. borderline tumor from carcinoma  Cytological specimen processing may be challenging; expertise required  For most lesions, lower sensitivity, specificity and accuracy | More expensive  Slightly higher level of pain and potential need for local anesthesia  Higher complication rate  Slightly longer tissue fixation and processing time |

*Differences between fine-needle aspiration and core-needle biopsy are mainly due to the technique itself, rather than needle gauge. H&E, hematoxylin and eosin staining; NGS, next-generation sequencing.

Diagnostic performance of image-guided biopsy is evaluated using the following parameters: adequacy, accuracy, safety and grading of diagnostic yield. **Biopsy adequacy** is defined as the property of the tissue sample to be sufficient and appropriate for histopathological and/or molecular diagnosis for a given purpose, such as clinical care or research; it is mainly related to the quality of biopsy and volume of the tissue sample[^81^](#_ENREF_81). The quality of a biopsy is improved by targeting the most solid component and dedifferentiated aspect of the target, the avoidance of necrotic areas. The biopsy is ideally obtained from the center of small lesions or on the edge of larger lesions. The volume of the tissue sample in core-needle biopsy is determined by the needle gauge, number of cores/needle passes, and length of each core. **Biopsy accuracy** is defined as the concordance between the pathologic findings on the tissue sample and the final histology on the surgical resection specimen[^82^](#_ENREF_82). **The safety of the procedure** and the risk of complications are related to the surgeon's experience, center volume (low vs high volume center), proper patient selection, careful selection of the biopsy path and close adherence to contraindications (see 4.3. Indications and contraindications for image-guided biopsy). **Diagnostic yield**, also referred to as the detection rate, is defined as the number of disease-positive samples detected by the procedure among all the samples[^83^](#_ENREF_83). Regarding the effect of test results on clinical decisions, the diagnostic yield is graded as grade 0 = insufficient sample for diagnosis, grade 1 = equivocal, grade 2 = consistent with histological diagnosis of pathology but a further diagnostic technique recommended for grading and typing purposes, grade 3 = fully diagnostic for tumor and sufficient information obtained for grading and typing purposes to allow initiation of treatment. Grades enabling treatment and considered actionable are grade 2 and 3, while all other results are considered non-actionable (grades 0-1).

- - - 1. *Core-needle (tru-cut) biopsy*

Core-needle biopsy can be performed with “side-cutting” needles (such as in tru-cut biopsy, with a side notch for tissue collection) or “end-cutting” needles (cylindrical tissue collection) (Figure S2)[^84^](#_ENREF_84).


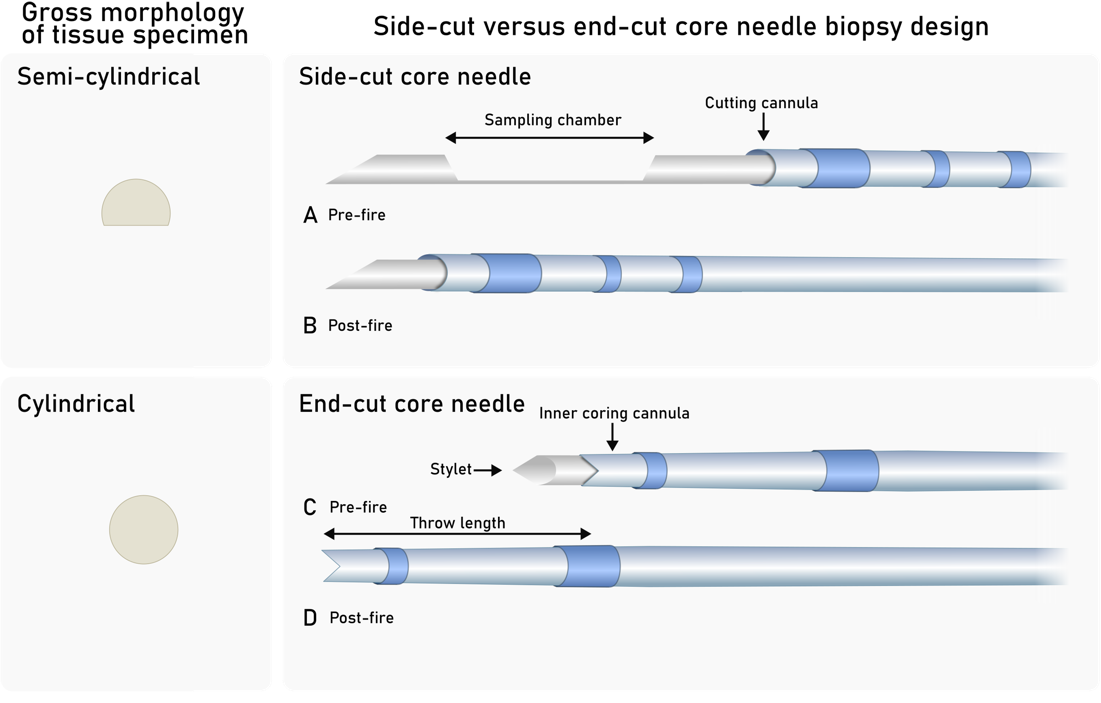


**Figure S2.** Image demonstrating different types of needles for core-needle biopsy (adapted from Carberry *et al.*[^66^](#_ENREF_66))

For needle caliber, Gauge (G) refers to an old, non-linear system for wire measurement, adopted for the measurement of the outer diameter of needles in medicine[^85^](#_ENREF_85). Currently, each Gauge is encoded by a different color by the International Society for Standardization[^86^](#_ENREF_86). Needle calibers for core-needle biopsy range generally from 14G to 18G (outer diameter from 2.1 mm to 1.3 mm). Core-needle biopsy uses a hollow needle with a spring-loaded cutting action, integrated in an automated, semi-automated or manual gun, to provide samples with preserved tissue architecture, allowing reliable histological evaluation (Figure S2). Moreover, the volume of the sample obtained is usually satisfactory to allow complex immunohistochemical examination or complex molecular testing (e.g., for sequencing nucleic acids or protein compounds) and to tailor individualized treatment.

Core-needle biopsy is recommended in the most recent guidelines for the management of various gynecologic cancers for histological diagnosis in non-resectable or inoperable cancers, to confirm the presence of metastases if imaging detects equivocal distant lesions (e.g., hepatic lesions, suspicious lymph nodes draining the primary tumor, and others), thus avoiding excision or open surgical biopsy of tumor tissue[^87-92^](#_ENREF_87). It is also recommended for suspicious uterine mesenchymal tumors (Figure S3) or suspected lymphoma subtyping when excisional biopsy is not feasible (Figure S4), while fine-needle aspiration is not indicated for initial diagnosis of these tumors[^93^](#_ENREF_93)^,^[^94^](#_ENREF_94). If there is a clear indication and informed consent, core-needle biopsy can be performed in the same setting, immediately after completion of an ultrasound examination.

- - - - 1. Adequacy and accuracy of core-needle biopsy

**The rate of adequate samples of core-needle biopsy of pelvic masses** is very high, ranging from 84 to 100%[^7^](#_ENREF_7)^,^[^12^](#_ENREF_12)^,^[^13^](#_ENREF_13)^,^[^25^](#_ENREF_25)^,^[^49^](#_ENREF_49)^,^[^75^](#_ENREF_75)^,^[^95-102^](#_ENREF_95). Adequacy of the biopsy may be influenced by performing center case volume, the sonographer’s experience and ability to choose the best area for biopsy and access to target (the shorter the distance between target and probe the better), the size of the lesion, the tumor’s characteristics (vital tumor included within the length of needle), the needle caliber (in Gauge), the length of the cylinder and number of acquired samples (cores/cylinders)[^7^](#_ENREF_7)^,^[^26^](#_ENREF_26)^,^[^57^](#_ENREF_57)^,^[^95^](#_ENREF_95)^,^[^97^](#_ENREF_97)^,^[^102-109^](#_ENREF_102). The use of a high-resolution endovaginal probe and a needle guide attached to the probe contribute significantly to the higher diagnostic outcome[^7^](#_ENREF_7). It has been reported that increasing the biopsy core length (greater penetration depth) and number of cores obtained (higher number of passes) ensure better sample adequacy[^109^](#_ENREF_109)^,^[^110^](#_ENREF_110). Verschuere *et al.* reported an increase in adequacy from 75% to 94.4% when one or two cylinders were obtained, respectively[^97^](#_ENREF_97). Fischerova *et al.* reported a success rate of 97.7% with two samples[^12^](#_ENREF_12). The possibility of rapid on-site evaluation (ROSE) by pathologists has been proposed as a method to improve the adequacy of biopsy[^111^](#_ENREF_111). This cytopathologic evaluation is applied especially to fine-needle aspiration samples but can also be applied to core-needle biopsies by “touch imprint”[^112^](#_ENREF_112)^,^[^113^](#_ENREF_113). Specific features of certain tumors can also influence the adequacy rate, being related to higher or lower amount of tissue withdrawal. Zikan et al. in a group of patients (n=190) with primary non-resectable or recurrent gynecologic cancers and suspected non-genital cancers showed that serous epithelial ovarian histotype, primary non-resectable tumor, the presence of ascites and/or carcinomatosis, elevated CA-125, and the vaginal approach were positive predictors of achieving adequate samples[^7^](#_ENREF_7). Conversely, non-ovarian masses, non-serous histotypes (which are commonly cystic and necrotic), recurrent cancer and the use of the transabdominal approach were negative predictors of adequate sample yield. Age, BMI, and prior hysterectomy did not influence the adequacy of the biopsy sample[^7^](#_ENREF_7).

**Both accuracy and diagnostic yield** are very high for core-needle biopsy in pelvic tumors, ranging from 73-100% depending on the site and origin of the lesion[^7^](#_ENREF_7)^,^[^12^](#_ENREF_12)^,^[^13^](#_ENREF_13)^,^[^25^](#_ENREF_25)^,^[^49^](#_ENREF_49)^,^[^76^](#_ENREF_76)^,^[^80^](#_ENREF_80)^,^[^97-100^](#_ENREF_97)^,^[^114^](#_ENREF_114)^,^[^115^](#_ENREF_115). The transvaginal approach is recommended due to the closer proximity from the probe to the target lesion, which allows a better depiction of the tumor morphology and its vital parts[^7^](#_ENREF_7). Nevertheless, a high accuracy rate was also reported for percutaneous ultrasound-guided biopsy (i.e. 93% to 99%)[^116-118^](#_ENREF_116). Of note, if the histological results from the core-needle biopsy are negative but there is a high suspicion of malignancy based on imaging findings, further investigation should be performed. Critical evaluation of the first attempt is mandatory before considering an optimized repeated procedure. The addition of cytopathology in combination with core-needle biopsy during the repeated procedure can also be considered.

In clinical practice, there are different automatic biopsy devices that allow different penetration depths (e.g. Bard® 15 or 22 mm) and therefore different needle core lengths. The minimum amount of tissue required for any specific analysis differs according to the exam purpose, the type of tissue and percentage of parenchyma in the core and the processing required by the pathologist[^119^](#_ENREF_119). Data suggest that for the molecular testing of epithelial tumors, a volume rate of 8 mm^3^ would ensure a test failure rate of 1%[^120^](#_ENREF_120). This is obtainable more than 99% of the time with two 10-mm-long cores of an 18G needle biopsy[^120^](#_ENREF_120). Accordingly, authors of this consensus statement agree that such an amount of tissue is expected to be satisfactory for epithelial tumors in terms of adequacy and accuracy for any purpose. Biopsies may serve to assess the molecular profile of the tumor, for which very few cells are usually needed and to plan a subsequent targeted therapy, such as for the identification of homologous recombination deficiency in the management of ovarian cancer and its treatment planning with poly ADP ribose polymerase inhibitors[^121-123^](#_ENREF_121). Currently even a minimal amount of tissue may be acceptable for next-generation sequencing (for more details, see 4.4.3. Processing of the sample)[^119^](#_ENREF_119)^,^[^124^](#_ENREF_124)^,^[^125^](#_ENREF_125). Successful sequencing has been reported with available DNA as little as 10 ng[^126^](#_ENREF_126).

For soft tissue tumors, core-needle biopsy is well established for histological diagnosis. In contrast, fine-needle aspiration should not be performed for the initial diagnosis of sarcomas but can be considered for confirmation of local recurrence of metastases of previously histologically confirmed soft tissue sarcomas[^94^](#_ENREF_94). In contrast to core-needle biopsy of epithelial tumors, core-needle biopsy of soft tissue tumors showed more non-diagnostic samples and lower accuracy due to technical sampling difficulties, especially related to the possible heterogeneity of the tumors (e.g. dedifferentiated liposarcomas), and absence of important criteria for diagnosis such as tumor growth margins due to sampling method[^7^](#_ENREF_7)^,^[^12^](#_ENREF_12)^,^[^127-129^](#_ENREF_127). Recent advances in imaging techniques helps to reduce sampling error by targeted biopsy of the least differentiated non-necrotic part of the soft-tissue tumor using MRI with intravenous contrast and diffusion weighted images and/or contrast-enhanced CT or PET-CT. Specialized ultrasound examination performed in a specialized cancer center by an expert sonographer has also demonstrated a similar ability to detect the mass, identify features associated with a greater likelihood of sarcoma and select an appropriate biopsy site(s)[^130^](#_ENREF_130). The combination of ultrasound-guided core-needle biopsy and subsequent genomic analysis of the sample using microscopic and array-Comparative Genomic Hybridization was described to achieve high accuracy (sensitivity, specificity, positive predictive value and negative predictive value of 100%) in detecting suspicious malignant or benign soft tissue tumors on MRI, compared to pathological reports after surgery[^131^](#_ENREF_131). Regarding the effect of needle size and number of passes on accuracy, available data are heterogeneous, as shown by a recently published meta-analysis[^16^](#_ENREF_16)^,^[^127^](#_ENREF_127). A meta-analysis by Kubo et al did not show any significant differences in complication rates for different needle gauges (</≥15G), number of samples (<5 or ≥5 cores or passes) or the use of ultrasound guidance[^127^](#_ENREF_127). The only significant factor was the operator type (surgeon or radiologist), suggesting that in soft tissue and bone sarcomas the radiologists were more skillful in performing core-needle biopsy that surgeons. However, another study by Weigl *et al.* showed that in the setting of a specialized cancer center, designated expert radiologists seemed not to be required and ultrasound-guided core-needle biopsy performed by the attending surgeon, preferably at first presentation of the patient in the outpatient clinic, shortened the time between first presentation and diagnosis and resulted in high safety and diagnostic accuracy[^132^](#_ENREF_132). Thus, the results are depending more on the expertise on the individual operator and less on the specialty the person belong to. In general, operator expertise rather than specialty (radiologist, gynecologist, surgeon) appears to more significantly affect the diagnostic yield. It is therefore appropriate that the biopsy is performed during the initial visit of a patient suspected to have cancer if a trained operator is available, rather than to have to schedule a subsequent biopsy by a radiologist. Alatzides *et al.* demonstrated that targeting specific areas in the tumor mass and the number of passes (a sample count ≥3), rather than the size of the needle (16G or 18G) used, improved the diagnostic yield (success rate) and accuracy[^129^](#_ENREF_129). Wu *et al.* showed no difference in diagnostic yield according to needle gauge for bone and soft tissue tumors, with diagnostic yield increasing with the number of samples obtained and with longer sample length; it reached a plateau at three samples for bone lesions and four samples for soft-tissue lesions[^133^](#_ENREF_133). The ESMO-EUROCAN-GENTURIS soft tissue and visceral sarcomas guidelines recommend 14G or thinner needles in correlation with imaging for biopsy of a suspected soft tissue tumor[^54^](#_ENREF_54). The 2024 ESGO-GCIG-EUROCAN uterine sarcomas guidelines recommend pre-operative ultrasound-guided trans-uterine cavity (in-organ) core-needle biopsy (≥14-16G) of the most suspicious lesion to be performed with expert pathologic review using microscopic and molecular analysis as needed (Figure S3)[^134^](#_ENREF_134). Percutaneous biopsy using coaxial needle (for less accessible masses) may be an option but should be used with caution in a specialized center[^131^](#_ENREF_131). To conclude, the caliber of needle for core-needle biopsy of uterine mesenchymal tumors is not different for other tumor entities and reflects the planned approach. In general for percutaneous and transcervical approaches wider (14-16G) needles are used, while for transvaginal/transrectal approach in gynecology thinner needles (18 G) with needle guide are used. In both approaches 3 passes (cores) are usually sufficient if containing adequate proportion of tumor.


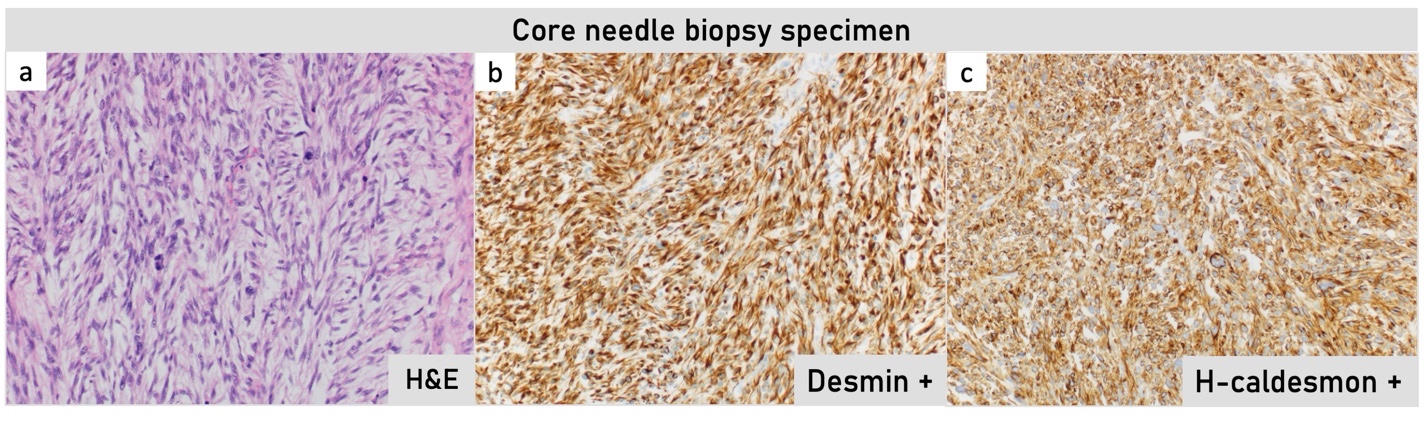
**Figure S3.** Demonstration of core-needle biopsy from a patient with leiomyosarcoma. (a) Spindle cell leiomyosarcoma (hematoxylin and eosin stain). (b) Positive immunohistochemical staining for desmin and (c) H-caldesmon+ showing cytoplasmatic positivity typical of leiomyosarcoma.

Genetic testing and immunophenotyping have become essential for the accurate classification of lymphoproliferative disorders. An incisional or excisional biopsy is the reference standard for subclassifying lymphomas as core-needle biopsy may be inadequate in some lymphoma subtypes and lead to cause treatment delay[^93^](#_ENREF_93). However, the advent of novel diagnostic techniques has led to an increasing use of core-needle biopsies for suspected lymphomas. Many centers worldwide now advocate core-needle biopsies as the first step in the diagnosis of patients with suspected lymphoma[^135^](#_ENREF_135). Contrary to core-needle biopsy, fine-needle aspiration specimens lose all architectural tissue information, and rely solely on individual cellular characteristics for diagnosis[^136^](#_ENREF_136)^,^[^137^](#_ENREF_137). Histologic features are still considered essential in the diagnosis of lymphoma, and therefore fine-needle aspiration in the context of tissue diagnosis for suspected lymphoma is not recommended[^93^](#_ENREF_93). The rationale for core-needle biopsy for initial tissue sampling is that it is quick, easy to obtain, avoids general anesthesia, and is inexpensive. An open biopsy requires surgical referral and operating room scheduling which may delay diagnosis by weeks and bears a higher risk of wound infection, bleeding, and nerve damage[^138^](#_ENREF_138). Moreover, some patients are too frail to undergo general anesthesia. Finally, the costs of excisional biopsies are estimated to be at least four times higher than ultrasound-guided core-needle biopsies[^139^](#_ENREF_139). On the other hand, a meta-analysis on core-needle biopsies for diagnosing lymphoma in patients with cervical lymphadenopathy showed that core-needle biopsy is inadequate in a small but significant percentage of cases (17.5% in this meta-analysis)[^37^](#_ENREF_37). There are lymphoma subtypes that are less accurately diagnosed with core-needle biopsy, such as T cell, Hodgkin’s lymphoma and indolent disease[^140^](#_ENREF_140). It is expected that the accuracy of core-needle biopsy would continue to improve with the advent of novel diagnostic techniques. In contrast to superficially located lymphoma, deeply seated lymph nodes adjacent to large vessels which are suspected of lymphomatous infiltration may not be accessible to core-needle biopsy. In the case of a non-diagnostic sample or where there is a difficult-to-access lymphoma, an open biopsy should be considered[^93^](#_ENREF_93). As lymphomas typically result in diffuse infiltration of lymph nodes, no differences to the needle caliber or number of passes as with uterine mesenchymal tumors are required (Figure S4).


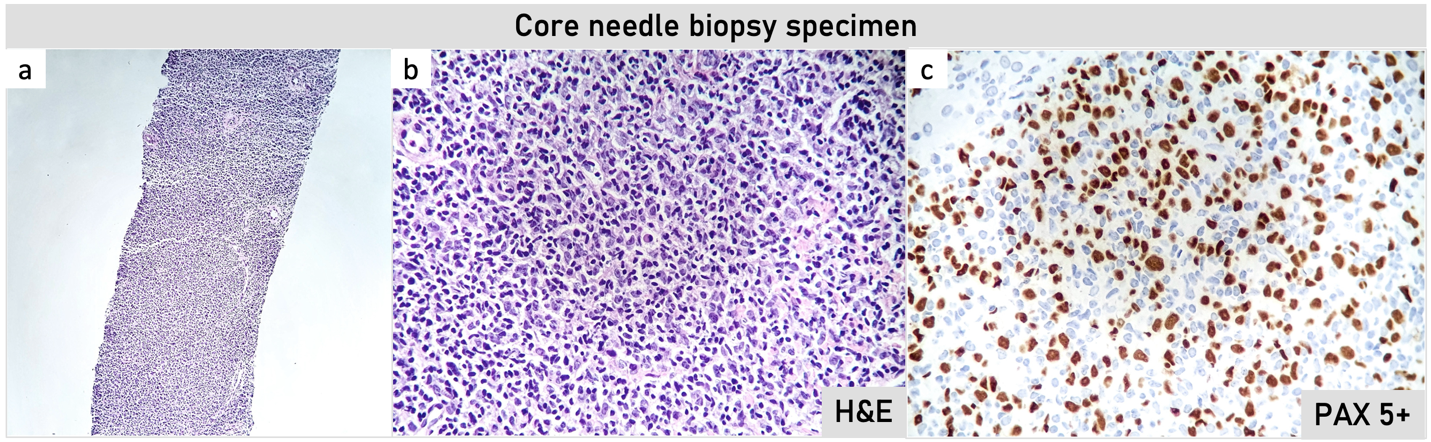
**Figure S4.** Demonstration of core-needle biopsy from a patient with diffuse large B cell lymphoma showing core biopsy specimen. (a) Low-power view of core-needle biopsy specimen of the lymph node. (b) Hematoxylin and eosin stain showing pathologic lymphoproliferation. (c) PAX5+ immunostaining showing nuclear positivity typical of B-cell lymphomas.

- - - - 1. Complications (safety) of core-needle biopsy

Surgical complications are often classified using the Revised Clavien Dindo Classification 2004, while the Society of Interventional Radiology adapted their Classification System for Complications based on clinical outcome (Table S2)[^141^](#_ENREF_141)^,^[^142^](#_ENREF_142). Major complications require therapy and result in admission to a hospital (for outpatient procedures), an unplanned increase in the level of care, prolonged hospitalization, permanent adverse sequelae, or death. Minor complications result in no sequelae and may require at most nominal therapy or a short hospital stay for observation (generally overnight). Early complications include bleeding, pain, infection, or organ injury, while late complications are caused by tumor seeding along the needle tract, leading to recurrences at the site of biopsy.

**Table S2.** Classification system for complications by outcome defined by the Society of Interventional Radiology[^142^](#_ENREF_142)

| *SIR classification system* |
| --- |
| Minor complications |
| a) No therapy, no consequence b) Nominal therapy, no consequence; includes overnight admission for observation only. |
| Major complications |
| c) Require therapy, minor hospitalization (<48 hours)  d) Require major therapy, unplanned increase in level of care, prolonged hospitalization (>48 hours)  e) Permanent adverse sequelae  f) Death |

SIR, Society of Interventional Radiology

The complications of biopsies can be further divided into generic and organ specific. Generic refers to complications that are common to all biopsies. The major generic complications include bleeding, infection, perforation, and unintended organ injury. Organ-specific complications are those that are associated solely or most commonly with biopsy of a specific organ, for instance hematuria after renal or prostate biopsy or hemoptysis after lung biopsy[^19^](#_ENREF_19)^,^[^20^](#_ENREF_20). In gynecology, the major complication rate of core-needle biopsy, using mainly the transvaginal approach, is low (between 0 to 1.4%) (Table S3)[^7^](#_ENREF_7)^,^[^12-14^](#_ENREF_12)^,^[^40^](#_ENREF_40)^,^[^100^](#_ENREF_100). The most frequent minor complications of the transvaginal approach are self-limited internal bleeding from the puncture site of the tumor and mild to moderate external vaginal bleeding, and mild pain/discomfort at the puncture site. There is mixed evidence regarding the association between needle gauge and risk of complications[^143-147^](#_ENREF_143). Smaller (thinner) needle sizes are regarded as less traumatic and may be associated with less complications[^107^](#_ENREF_107)^,^[^108^](#_ENREF_108). More importantly, high volume centers and experienced operators result in better patient outcomes, faster procedures, lower complication risk and less tissue damage of retrieved specimens. No higher complication rate was found comparing obese and non-obese women and patients under antiangiogenic therapy for gynecological cancers[^7^](#_ENREF_7)^,^[^28^](#_ENREF_28). Regarding the percutaneous approach, ultrasound-guided percutaneous peritoneal biopsy has a lower complication rate compared to organ biopsy[^148-150^](#_ENREF_148). Retrospective and prospective single-center studies of liver and abdominal organ biopsies with large numbers using a needle diameter >1.0 mm (<18G) have shown complication rates up to 2.5%[^78^](#_ENREF_78)^,^[^145^](#_ENREF_145)^,^[^151-153^](#_ENREF_151). For comparison, a meta-analysis of soft-tissue sarcomas also showed a low risk of complications with core-needle biopsy using the percutaneous approach, with a pooled complication rate of 1%[^16^](#_ENREF_16). Similarly, bleeding requiring surgery was seen in 1% after core-needle biopsy of abdominal lymph nodes[^7^](#_ENREF_7).

**Table S3.** Adequacy, accuracy, and diagnostic yield of core-needle biopsy in gynecological cancers (studies with ≥30 cases)

| Author, year | Study design | Patients (n) | Approach | Needle caliber  (Gauge) | Adequacy (n, %) | Accuracy (a) or Diagnostic Yield (y)^‡^ | Patients included for accuracy and diagnostic Yield assessment | Rate of major complications (n, %) |
| --- | --- | --- | --- | --- | --- | --- | --- | --- |
| Asp et al., 2023[^102^](#_ENREF_102) | R | 300 | TV, TA | 16-18 | 259 (86.3)† | 97.5 (a) | 159 | 4 (1.3) |
| Buonomo et al. 2022[^99^](#_ENREF_99) | R | 42 | TV, TA | 16, 18 | 42 (100.0) | 88.2^d^ (a) | 16 | 0 (0.0) |
| Epstein et al., 2016[^13^](#_ENREF_13) | P | 143 | TV, TR, TA | 16, 18 | 126 (88.0) | 100.0 (NS) | 30 | 2 (1.4) |
| Fischerova et al., 2008[^12^](#_ENREF_12) | P | 86 | TV, TA | 14, 16 | 80 (93.0) | 97.7 (a) | 86 | 1 (1.1) |
| Gao et al., 2019[^49^](#_ENREF_49) | R | 40 | TV, TR | 18 | 40 (100.0) | NS | NS | 0 (0.0) |
| Griffin et al, 2009[^65^](#_ENREF_65) | R | 60 (35 US-guided) | TA | 14, 16, 18 | 52 (86.6) | 87.0 (y) | 52 | 0 (0.0) |
| Kawamura et al., 2002[^52^](#_ENREF_52) | Clinical trial | 453 | TV (transcervical) | 16, 17 | 462 (96.3)* | NS | NS | 0 (0.0) |
| Kong et al., 2016[^154^](#_ENREF_154) | R | 52 | TV | 18 | 49 (94.2) | 94.2 (NS) | 52 | 0 (0.0) |
| Lengyel et al. 2021[^25^](#_ENREF_25) | P | 303 | TV, TR | 18 | 299 (98.7) | 87.2 (a) | 94 | 3 (1.0) |
| Lia et al., 2022[^155^](#_ENREF_155) | R | 318 | NS | 14 | NS | 94.7 (a) | 318 | NS |
| Lin et al.,2017[^95^](#_ENREF_95)** | R | 200 | TV | 18 | 192 (96.0) | 95.0 (a) | 192 | 0 (0.0) |
| Malmström et al., 1997[^80^](#_ENREF_80) | NS | 85 | TV, TR, TA | 18 | 75 (88.0) | 73 (NS) | 85 | 0 (0.0) |
| Mascilini et al., 2019[^96^](#_ENREF_96) | R | 62 | TV | 18 | 62 (100.0) | 80.0 (a) | 10 | 0 (0.0) |
| Mascilini et al., 2023[^101^](#_ENREF_101) | P | 128 | TV | 18 | 128 (100.0) | 94.0 (a) | 102 | 0 (0.0) |
| Oge et al., 2013[^156^](#_ENREF_156) | R | 55 | TV, TA | 18 | 53 (96.4) | NS | NS | 0 (0.0) |
| Park et al., 2016[^98^](#_ENREF_98) | R | 55 | TV | 18 | 55 (100.0) | 93.0 (y) | 51 | 12 (22.0)^a^ |
| Pelayo-Delgado et al., 2023[^157^](#_ENREF_157) | R | 34 | TV | 18 | 34 (100.0) | 100.0 (a) | 10 | 0 (0.0) |
| Verschuere et al., 2021[^97^](#_ENREF_97) | R | 108 | TV | 18 | 91(84.3)^b^ | 97.2 (a) | 36 | 0 (0.0)^c^ |
| Vlasak et al., 2020[^100^](#_ENREF_100) | R (and P) | 79 | TR, TA | 14, 16, 18 | 76 (96.2) | 90.2 (NS) | 51 | 1 (1.3) |
| Won et al., 2019[^48^](#_ENREF_48) | R | 30 | TV, TR | 18 | 28 (93.3) | 89.3 (a) | 28 | 0 (0.0) |
| Zikan et al., 2010[^7^](#_ENREF_7) | R | 190 | TV, TA | 18 | 178 (93.7) | 98.3 (a) | 118 | 2 (1.0) |
| Hewitt et al., 2007[^40^](#_ENREF_40) | R | 149  (60 US-guided) | NS | 18 | 139 (93.0) | 90 (US) (y)  91 (CT) (y) | 60 (US)  89 (CT) | 1 (<1.0) |

^a^ Vaginal bleeding in 10 patients (18%); Gross hematuria in two patients (4%). ^b^ Single tissue cylinder 75%; Two cylinders 94.4%; More than two cylinders 100%. ^c^ Minor complications 4.5%. ^d^ Cases with at least two diagnostic samples: 94.1%. *Based on number of samples (i.e. 462/480). **Detection rate was calculated on 192 patients of whom 96 had surgical confirmation. † Based on 159 out of 177 patients. ‡ (a) Accuracy = ability of a test to detect a condition when it is present and to detect the absence of a condition when it is absent (i.e. accuracy = (TN + TP)/(TN+TP+FN+FP)). (y) Diagnostic yield (also referred to as detection rate) = the number of disease-positive patients detected by a diagnostic test divided by the total cohort size (i.e. DY = TP/(TN+TP+FN+FP)). CT, computed tomography; NS, not specified; TA, transabdominal or percutaneous; TR, transrectal; TV, transvaginal; US, ultrasound.

Bleeding:

After transvaginal biopsy, minor bleeding was observed in 18% of 55 women by Park et al. and in all 62 patients in a series by Mascilini *et al.*, with rapid spontaneous resolution in all the cases. In reports of consecutive procedures, the risk of major bleeding is low but not absent. In a retrospective analysis of 195 biopsies by Zikan *et al.*, two women (1%) developed major complications that required surgery[^7^](#_ENREF_7)^,^[^98^](#_ENREF_98)^,^[^101^](#_ENREF_101). One patient presented with intermittent but self-limited intra-peritoneal bleeding from the biopsy site (‘fountain sign’/turbulent flow) which resolved spontaneously as confirmed by immediate laparoscopy. The second case had mild thrombocytopenia (80 × 10^9^/L) and developed internal bleeding from the biopsy site of a Krukenberg tumor, resulting in hemoperitoneum and the need for adnexectomy[^7^](#_ENREF_7). Regarding the percutaneous approach, tumor infiltration of the greater omentum is often used in gynecologic oncology as a target for percutaneous core-needle biopsy when there is no suitable target in the pelvis for an adequate biopsy[^116^](#_ENREF_116)^,^[^118^](#_ENREF_118)^,^[^148-150^](#_ENREF_148)^,^[^158^](#_ENREF_158). The infiltrated omentum is usually located between the anterior abdominal wall and the intestinal loops with safe accessibility and in close proximity to the ultrasound probe. Omental biopsy has been shown to be safe and effective, with a low risk of complications, despite the omentum having a vast vascular structure. Perez *et al.* did not observe any complication among 163 patients undergoing core-needle biopsy or fine-needle aspiration of infiltrated omentum, while Wang *et al.* suggested the use of the transducer to exert pressure on the site of the biopsy to prevent subsequent bleeding despite the absence of major bleeding in their series of 153 patients[^116^](#_ENREF_116)^,^[^118^](#_ENREF_118). Souza *et al.* and Que *et al.* reported the occurrence of minor complications in 2.7% and 2% of patients respectively after omental biopsy. Another complication, which is related to the percutaneous transabdominal approach, was abdominal wall hematoma, as reported by Epstein *et al.* and Hewitt *et al.*[^13^](#_ENREF_13)^,^[^40^](#_ENREF_40)^,^[^149^](#_ENREF_149)^,^[^150^](#_ENREF_150). In the study by Hewitt et al., one case out of 149 developed a hematoma in the rectus sheath. Epstein *et al.* reported 1 similar case out of 10 percutaneous biopsies, probably related to the patient’s use of low-molecular weight heparin[^13^](#_ENREF_13). To avoid abdominal wall hematomas, the authors of this consensus statement recommend careful identification and avoidance of the inferior epigastric vessels which lie between the rectus abdominis muscle and the posterior lamella of its sheath before insertion of the biopsy needle.

A prospective study in France of 2082 liver biopsies showed the rate of severe complications was 0.57% and increased with the number of needle passes (one pass vs 2 and more passes (p<0.001))[^159^](#_ENREF_159). Another prospective study in Germany of 8172 intraabdominal interventions did not show a significant increase in major bleeding complications with 2 needle passes versus 1 needle pass and more than 2 needle passes versus 1 needle pass[^160^](#_ENREF_160). Other studies have also reported that the number of needle passes has no effect on the rate of post-biopsy complications[^161^](#_ENREF_161)^,^[^162^](#_ENREF_162). Some authors of this consensus statement have postulated that side-cut needles are associated with a lower risk of bleeding and better tissue quality than edge-cut needles, but there is insufficient evidence to support this.

Infections:

Procedure-related infections and fever are also possible complications after transvaginal biopsy, highlighting the importance of proper vaginal cleansing before the procedure. Epstein *et al.* reported a single case of infection after transvaginal core-needle biopsy out of 131 such procedures, while Lengyel *et al.* described 3 cases of infection out of 149 transvaginal biopsies of cystic lesions that secondarily developed abscesses[^13^](#_ENREF_13)^,^[^25^](#_ENREF_25). Of note in Lengyel’s study, all 3 infectious complications occurred during the first 149 biopsy procedures (49.2%) when vaginal disinfection was not routinely used. In the following 154 patients (50.8%), routine vaginal disinfection was performed, and no more infectious complications occurred. Injury of the intestinal wall, in the case of a small, single disruption with a needle, is generally associated with a low risk of leakage of intestinal contents and peritonitis. Observation and conservative management are preferred to surgery as no related subsequent complications have been reported in the literature[^163^](#_ENREF_163). If there is incidental puncture of the bladder, antibiotic therapy can be considered, taking into account any pre-procedural antibiotic prophylaxis which may already have been given[^164-166^](#_ENREF_164). Regarding the transrectal approach, the needle may not always penetrate the peritoneal cavity, as in the case of pelvic retroperitoneal lymph nodes or cul-de-sac carcinomatosis obliteration. In such cases of biopsies of an extra-peritoneal location, the need for antibiotic administration is unclear. Indeed, studies of bacteremia and complications during perirectal lesion biopsies via the lower gastrointestinal tract report a low risk of infectious complications and prophylaxis is therefore not routinely recommended[^164^](#_ENREF_164)^,^[^167^](#_ENREF_167).

Percutaneous ultrasound-guided biopsy of peritoneal lesions is considered a safe procedure with a low risk of infectious complications[^118^](#_ENREF_118). Most lesions are adjacent to the abdominal wall which means a low risk of iatrogenic injury to the underlying bowel[^158^](#_ENREF_158). Incidental puncture of the small bowel should not be of major concern due to the muscle-sealing wall and lower risk flora in contrast to the large bowel, which should be avoided due to the risk of peritonitis[^167^](#_ENREF_167)^,^[^168^](#_ENREF_168). Several authors reported that biopsy is safe and well-tolerated even when performed through the bowel wall[^163^](#_ENREF_163)^,^[^164^](#_ENREF_164)^,^[^169-171^](#_ENREF_169). In the literature, only one case of abscess formation has been reported after transcolonic mesenteric biopsy[^172^](#_ENREF_172).

In general, the risk of infectious complications during ultrasound-guided biopsy in gynecology is low and antibiotic prophylaxis is not routinely recommended for any approach. The development of a hematoma is a risk factor for abscess formation and should prompt consideration of antibiotic therapy.

Organ injury:

The occurrence of injury to the hollow organs or viscera on the path to the target lesion during the biopsy does not necessitate immediate surgery unless there is concern of hemorrhage. There are reported cases of biopsy using a trans-organ route (through stomach, colon, small bowel, liver, pancreas and spleen), with no occurrence of major complications[^171^](#_ENREF_171)^,^[^173-175^](#_ENREF_173). In a retrospective series of 22 pancreatic biopsies, a “trans-organ” route for the procedure was proposed, with intentional direct pathway traversing the stomach, colon, small bowel, liver and spleen, resulting in three (13.6%) minor hematomas, but no major complications[^175^](#_ENREF_175).

Vasovagal reaction:

Vasovagal symptoms result from a sudden increased tone of parasympathetic receptors and are commonly related to handling of the cervix or uterine cavity[^176^](#_ENREF_176). Despite being a common concern during gynecological procedures, vasovagal reactions and syncope are unusual during core-needle biopsy in gynecology. A single case of self-limiting vasovagal reaction was reported by Franchi *et al.* in 337 patients undergoing transvaginal core-needle biopsy[^114^](#_ENREF_114). Zanetta *et al.* reported 17 vagal symptoms over 1000 gynecological procedures, with only one case requiring hospitalization following the percutaneous drainage of a lymphocele[^177^](#_ENREF_177).

Tumor seeding:

There is a risk of tumor cell dissemination associated with biopsy due to needle tract seeding/implantation or intraperitoneal dissemination with tumor upstaging. Biopsy needle tract seeding is the dissemination and proliferation of neoplastic cells in the tissue along the needle tract and is usually regarded as a late complication. The incidence of needle tract seeding may be underestimated if recurrence is not recognized to be a direct result of this intervention or due to short follow-up duration. According to quality improvement guidelines, the rate of this complication should not exceed 5%[^20^](#_ENREF_20). The risk of needle tract seeding appears to be related to tumor histology, location, and biopsy approach. For advanced ovarian cancer, Griffin *et al.* did not observe any case of needle tract seeding in 60 patients undergoing percutaneous biopsy for ovarian cancer[^65^](#_ENREF_65). Identical findings were reported in a study by Thabet *et al.* on a series of 27 patients with ovarian cancer in whom no occurrence of implants was observed after transvaginal or percutaneous biopsies during the follow-up period of 45 months[^178^](#_ENREF_178). Differing results have been published for non-genital cancers. For suspected hepatocellular carcinoma, a meta-analysis by Silva *et al.* showed the overall risk of needle tract seeding following biopsy to be 2.7%, while Kim *et al.* reported a 3.4% incidence[^179^](#_ENREF_179). Of note, in percutaneous liver biopsy before liver metastasectomy of colorectal cancer, early progression in needle tract sites after percutaneous liver biopsy has been described[^180^](#_ENREF_180)^,^[^181^](#_ENREF_181). Biopsy of a malignant renal mass poses a 0.01% to 6% risk of needle tract seeding risk. Using an 18G needle and a coaxial technique are recommended techniques to minimize this risk, and it is important to ensure adequately long follow-up[^182^](#_ENREF_182)^,^[^183^](#_ENREF_183). For retroperitoneal soft tissue tumors/sarcomas, the overall risk also appears to be low (<1%), except in a study by Van Houdt *et al.*, where 2% of cases developed a biopsy-related recurrence[^184-187^](#_ENREF_184). In Van Houdt’s study, a lower risk was hypothesized when using a retroperitoneal rather than transperitoneal approach when taking biopsies of the retroperitoneal sarcoma[^184^](#_ENREF_184). In cases where the transperitoneal route has been used, the removal of tissue around the route of biopsy is advisable during subsequent surgery[^187^](#_ENREF_187). Although the evidence is scarce, some authors have demonstrated that coaxial systems protect the needle tract from surrounding normal tissue and may reduce the risk of tumor seeding[^184^](#_ENREF_184). Using the same needle for multiple biopsies in the same patient may inadvertently result in the implantation of microscopic tumor particles, therefore some authors suggest cleaning the needle cannula with a dry, sterile cloth to remove any such particles prior to subsequent biopsy passes[^182^](#_ENREF_182). Studies reporting local recurrence in the biopsy route have not shown an impact on the overall survival[^184^](#_ENREF_184)^,^[^187^](#_ENREF_187). Interestingly, for intra-abdominal soft tissue tumors such as gastrointestinal stromal tumors, no increased risk of recurrence was reported for the percutaneous transperitoneal approach in comparison to the endoscopic approach[^188^](#_ENREF_188).

In gynecologic oncology, to reduce the risk of intraperitoneal seeding, core-needle biopsy of a well-encapsulated mass located in the peritoneal cavity should be avoided except after multidisciplinary review of patient data and imaging results, and careful consideration of the risks and benefits of biopsy as part of the individual patient’s management. The risk of peritoneal dissemination and tumor upstaging is one of the relative contraindications to core-needle biopsy in gynecologic oncology because it may affect the patient’s prognosis. Care should also be taken during in-organ biopsy (e.g., for suspected cervical tumor or uterine mesenchymal tumor) to avoid needle passage through the uterine serosa and contamination of the peritoneal cavity. For retroperitoneal or subperitoneal tumors incidentally detected during gynecological scan in the pelvis or referred for biopsy by another specialist, the biopsy site and the needle trajectory should be carefully evaluated by ultrasound before the procedure. This allows assessment of the feasibility of obtaining biopsy via the transvaginal or transrectal approach while avoiding contamination of free spaces such as the abdominal cavity, injury to surrounding critical structures such as iliac vessels, and to minimize disruption of fascial planes.

- - - 1. *Fine-needle aspiration*

Fine-needle aspiration is used to withdraw cellular material or fluid from a lesion. The collected material is usually expelled onto multiple glass slides creating smears or into a container with a fixative such as formalin. The glass slides are stained with various specialized dyes to identify specific cell types or structures. Under the microscope the pathologist evaluates cell morphology, structure, and other characteristics to perform a diagnosis. The difference between fine-needle aspiration and core-needle biopsy lies mainly within the technique itself, rather than the needle gauge.

The process of fine-needle aspiration allows mostly cells to be obtained, whereas core-needle biopsy collects entire cores of tissue. Needles used are up to 300mm in length and usually between 20 and 25 Gauge, although thicker needles (17-18 Gauge) are available as well. The needle is often connected to a syringe, the plunger of which can be used to apply negative pressure to help to aspirate the specimen, thereby obtaining a cytological specimen. Many needle tips are available and may be chosen according to the characteristics of the target and for ease of needle tracking on ultrasound (Figure S5)[^46^](#_ENREF_46)^,^[^66^](#_ENREF_66).


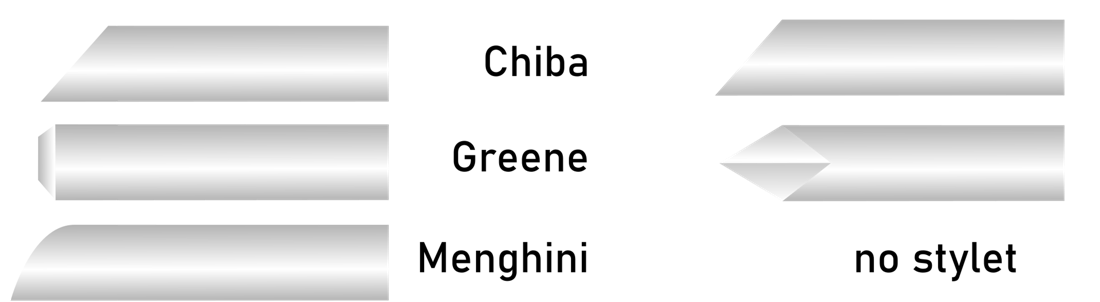


**Figure S5** Different needle types used in fine-needle aspiration. Image showing the different needle types used in fine-needle aspiration with outer cannula (left side) and inner stylet (right side). The Chiba needle is a two-part hollow needle with a beveled tip. The Greene needle has a sharp inner stylet which facilitates the initial puncture and rounded noncutting bevel. The Menghini-tip needle does not have a stylet and has a tapered beveled edge that facilitates the tissue being withdrawn into the lumen.

Generally, a thicker needle may be used when higher control is needed within the tissue, as it tends to deviate less than a thinner needle. Similarly, shorter needles deflect less than longer needles. Fine-needle aspiration is one of the least invasive procedures to obtain a biological material sample, with low morbidity and does not require anesthesia.

Fine-needle aspiration only allows cytological evaluation. It yields sufficient sample for tumoral cell assessment, however tissue architecture is not properly preserved, and samples for immunocytochemistry or genomic assessment may be limited[^189^](#_ENREF_189). Several studies on the pathological accuracy of intra-operative fine-needle aspiration described the technique as a valuable predictor of malignancy, although in most circumstances definite pathological diagnosis cannot be made on aspiration alone[^190-193^](#_ENREF_190). Based on that, fine-needle aspiration may be sufficient when managing recurrent tumors, but it is a limitation when seeking the diagnosis of a primary tumor at initial presentation. It has also been described as a valuable tool in the sampling of infiltrated lymph nodes in patients with a previous history of malignancy, or in patients without such a history. However, in the latter group, carcinoma with unspecified histotypes was more frequently detected[^194^](#_ENREF_194). Fine-needle aspiration represents an alternative diagnostic technique in more challenging or difficult to access locations, such as the extra-pelvic retroperitoneum, or in patients with high bleeding risk[^195^](#_ENREF_195)^,^[^196^](#_ENREF_196). It may also be a reliable alternative to core-needle biopsy for tumor next-generation sequencing as comparative analysis of the SHIVA02 trial observed that both techniques were equally suitable for molecular assays[^197^](#_ENREF_197). Kanagal-Shamanna *et al.* showed that mutation profiling can reliably be obtained from fine-needle aspiration smears, with fine-needle aspiration being non-inferior to core-needle biopsy for tumor cellularity, tumor fraction and sequencing metrics[^198^](#_ENREF_198)^,^[^199^](#_ENREF_199). Moreover, fine-needle aspiration smears could benefit from better preservation of nucleic acids than core-needle biopsy and enriched tumoral cell heterogeneity, due to their inherent tissue collection method[^200^](#_ENREF_200). An advantage of fine-needle aspiration is its therapeutic role, such as to aspirate cysts or where there is ascites present. Adnexal cyst aspiration has a low probability of permanent resolution (33-46%) and high recurrence rate (20-44%), and its use should be reserved for selected cases such as in symptomatic patients not suitable for surgery[^195^](#_ENREF_195)^,^[^201^](#_ENREF_201)^,^[^202^](#_ENREF_202). In order not to puncture encapsulated ovarian cancer, ultrasound features of benignity such as the benign modified descriptors should be used[^203^](#_ENREF_203).

Adequacy and accuracy of fine-needle aspiration:

Overall reported adequacy and accuracy of fine-needle aspiration procedures to establish whether a lesion is benign or malignant ranges between 74-100% and 73-99%, respectively[^80^](#_ENREF_80)^,^[^193^](#_ENREF_193)^,^[^204-207^](#_ENREF_204). Moreover, it is a feasible tool for the collection of samples from suspicious lymph nodes, with a high reported accuracy of 97.7% to discriminate between benign and malignant lymph nodes, although data pertaining to gynecology are sparse[^208^](#_ENREF_208). The possibility to perform a rapid onsite evaluation (ROSE) has shown to significantly improve the adequacy of the sample[^111^](#_ENREF_111).

Safety of fine-needle aspiration:

Possible complications of fine-needle aspiration are similar to those of core-needle biopsy and include bleeding, infections and trauma to the underlying organs, although these occur infrequently[^209^](#_ENREF_209). Table S4 demonstrates data on adequacy, accuracy and safety of fine-needle aspiration.

**Table S4.** Adequacy and detection rate of ultrasound-guided fine-needle aspiration (FNA) in gynecology in studies from 1990-2022

| Author, year | Study design | Population | Approach | Sampling type | Needle caliber (Gauge) | Adequacy (%) | Detection rate (%) | Rate of major complications (%) |
| --- | --- | --- | --- | --- | --- | --- | --- | --- |
| Layfield et al., 1991[^204^](#_ENREF_204) | NS | 58 | NS | FNAC | 23 | NS | 73.0 | NS |
| Imachi et al, 1992[^210^](#_ENREF_210) | NS | 287 | NS | FNAC | 22-23 | 84.2 | 95.2 | 0 (0.0) |
| Zanetta et al, 1994[^205^](#_ENREF_205) | NS | 101 | TV | FNAB | 18-19 | 83.7 | 91.0 | 0 (0.0) |
|  |  |  |  | FNAC | 19-21 | 75.6 | 83.0 |  |
| Malmström et al., 1997[^80^](#_ENREF_80) | NS | 85 | TV, TR, TA | FNAC  (vs CNB) | 23 | 86.0 | 79.0 | 0 (0.0) |
| Stockberger et al., 1999[^211^](#_ENREF_211) | R | 258* | TA | FNAB | 18-25 | 100.0† | 96.1 (≤ 21G) and 98.9 (≥ 20G) | 7 (2.7) |
| Pisharodi et al, 2000[^212^](#_ENREF_212) | R | 26 | TV | FNA | 23-25 | 92.3 | NS | NS |
| Gupta et al, 2011[^206^](#_ENREF_206) | R | 584 | TA | FNAC | NS | 73.5 | 76.0 | NS |
| Ray et al, 2014[^213^](#_ENREF_213) | NS | 83 | TV, TA | FNAC | 22 | NS | 93.0 | NS |
| Lin et al, 2016[^214^](#_ENREF_214) | R | 39**‡** | NS | FNA | 23-25 | 98.0 | NS | NS |
| Eitan et al., 2017[^207^](#_ENREF_207) | R | 59 | TV | 33 FNAB  26 FNAC | 17 | 88.0 | 84.7 | 0 (0.0) |
| Gupta et al., 2020[^193^](#_ENREF_193) | R | 580 | NS | FNAC | 22 | 88.8 | 88.0 | NS |
| Penna et al., 2022[^15^](#_ENREF_15) | R | 60 | TV | 43 FNAB | 20-22 | 85.0 | NS | 0 (0.0) |
|  |  |  |  | 17 FNAB + CNB |  | 92.0 |  |  |

*93 ultrasound-guided biopsies and 165 CT-guided; †100%for ultrasound-guided biopsies vs 93.2% CT-guided; ‡ 7 biopsies ultrasound-guided and 32 guided by other imaging method. CNB, core-needle biopsy; FNAB, fine-needle aspiration biopsy; FNAC, fine-needle aspiration cytology; NS, not specified; R, retrospective.

In general, the least invasive procedure that provides all necessary diagnostic information should be chosen. Image-guided biopsy is less invasive than open or excisional biopsy and is associated with lower morbidity. The choice whether to perform fine-needle aspiration or core-needle biopsy should be based on the diagnostic intent, whether to establish a primary diagnosis versus recurrent disease, the overall risk of the procedure, the confidence and experience of the clinician with the biopsy technique, and the availability of an experienced pathologist or cytopathologist (Figure 3). Core-needle biopsy is generally the method of choice, because it allows accurate diagnosis in a significantly higher number of cases compared to fine-needle aspiration[^215^](#_ENREF_215)^,^[^216^](#_ENREF_216). This is due in particular to the fact that core-needle biopsy allows assessment of tumor architecture and the relationship between tumor and stroma, which is essential for correct diagnosis in some tumors such as low-grade serous carcinoma and serous borderline tumor[^209^](#_ENREF_209)^,^[^217^](#_ENREF_217). Diagnosis of some mesenchymal or fibrotic lesions may be difficult or impossible from fine-needle aspiration samples, due to the low yield of tumor cells[^218^](#_ENREF_218). Moreover, a quantitatively sufficient material is needed for subsequent investigations, especially immunohistochemistry and molecular tests. The most frequently encountered complications for both techniques are minor, and an overall rate of major complications (< 1.5%) is low (Tables S2 to S4). Given the advantages of core-needle biopsy in gynecological oncology, the main emphasis in the following sections will be this technique, although the indications, contraindications, biopsy technique, reporting and training are very similar to those for fine-needle aspiration[^4^](#_ENREF_4)^,^[^11^](#_ENREF_11)^,^[^15^](#_ENREF_15)^,^[^19-21^](#_ENREF_19).


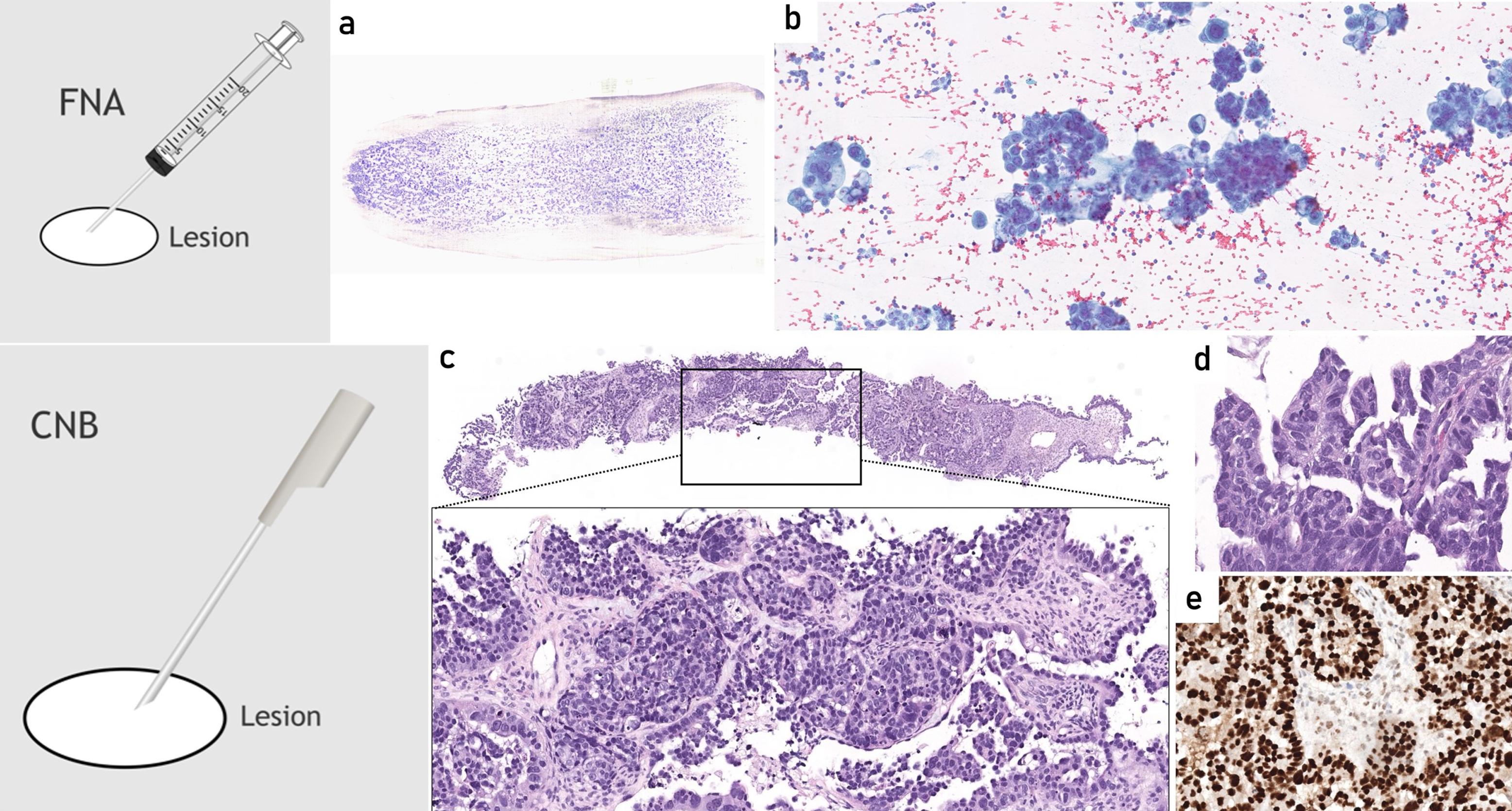


**Figure 3** Comparison of fine-needle aspiration (FNA) (a,b) and core-needle biopsy (CNB) (c–e) specimens. (a) Macroscopic appearance of the cytological smear obtained from ascitic fluid (May-Grünwald-Giemsa stain) from a woman with findings suggestive of ovarian cancer and (b) high-power view of carcinoma cells from the ascitic fluid (Papanicolaou stain). (c) Low-power view of CNB specimen of the omentum (hematoxylin and eosin (H&E) staining) infiltrated by high-grade serous carcinoma. (d) High-power view of carcinoma cells with hyperchromatic and pleomorphic nuclei, and mitoses (H&E staining). (e) Immunohistochemical staining with p53 positivity in all cells, with strong and diffuse nuclear expression compatible with high-grade serous carcinoma.

**Statement 9**: Two sampling techniques can be used for image-guided biopsy: core-needle biopsy and fine-needle aspiration.

- Level of evidence: 3a
- Grade of statement: B
- Consensus: yes, 94% (*n* = 17); no, 0% (*n* = 0); abstain, 6% (*n* = 1)

**Statement 10**: The choice between core-needle biopsy and fine-needle aspiration depends on the specific clinical situation. However, core-needle biopsy is preferable to fine-needle aspiration, as it allows tumor tissue to be obtained for biopsy examination, including ancillary methods, and it provides a larger volume of tumor tissue for multiple analyses, including molecular methods.

- Level of evidence: 4
- Grade of statement: C
- Consensus: yes, 94% (*n* = 17); no, 0% (*n* = 0); abstain, 6% (*n* = 1)

**Statement 11**: In order to ensure adequacy, high accuracy, diagnostic yield and safety of core-needle biopsy, it is recommended to obtain at least two 10-mm-long cylinders using a needle that is 18 G or wider. This provides enough tissue for diagnostic, molecular and genetic purposes for epithelial tumors and their metastases (peritoneal, lymphatic, parenchymal). At least three such cores are recommended for uterine mesenchymal tumors and lymphomas.

- Level of evidence: 3b
- Grade of statement: C
- Consensus: yes, 89% (*n* = 16); no, 0% (*n* = 0); abstain, 11% (*n* = 2)

## Indications and contraindications

The goal of the image-guided biopsy is to collect an adequate and accurate sample for the intended diagnostic, molecular or genomic tests in the timeliest fashion. The procedure aims to employ the safest needle path using an appropriate approach and transducer. It is essential to ensure proper patient selection and valid clinical indication prior to the biopsy. The risks of the diagnostic procedure should be assessed, to ensure that the procedure-related risks do not outweigh the potential benefits. The Society of Interventional Radiology defined the indications for biopsy to 7 major groups: (1) to establish the nature (benign or malignant) of a lesion, (2) to obtain material for microbiological analysis in case of infection, (3) to stage disease with known or suspected malignancy when local spread or distant metastases are suspected, (4) to identify residual or recurrent disease after initial treatment, (5) to determine the nature and extent of certain diffuse parenchymal diseases (hepatic cirrhosis, glomerulonephritis etc.), (6) to determine the primary origin in patients with metastatic disease or unknown primary cancer and (7) to obtain samples for biomarker, protein or genotype analysis to guide targeted therapy[^19^](#_ENREF_19). In gynecologic oncology, any of these indications may also apply, although with different representation per group. The indications for core-needle biopsy are demonstrated in different clinical cases in Videoclip S4. An overall representation of the indications for core-needle biopsy in gynecology and gynecologic oncology is shown in figure S6.


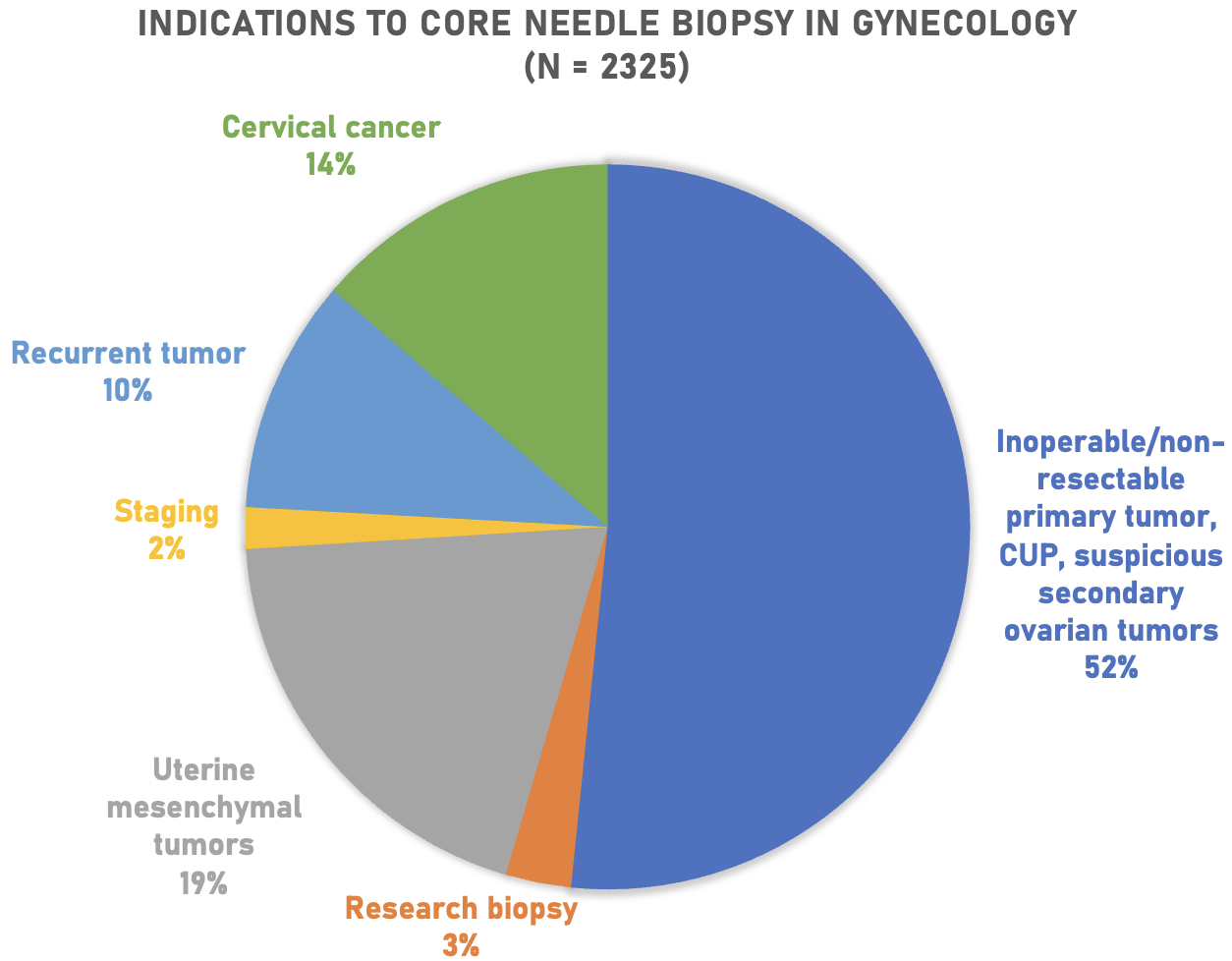


**Figure S6.** Summary of main indication for core-needle biopsy in gynecology/gynecologic oncology based on studies reporting ≥30 patients

CUP - cancer of unknown primary

- - 1. Indications

In gynecologic oncology, the most common indication for core-needle biopsy is the histological diagnosis of tubo-ovarian carcinomas in patients who are assessed to be unsuitable for primary radical procedures due to poor performance status (i.e. inoperability) or when complete cytoreductive surgery is deemed infeasible due to disseminated disease (i.e. non-resectability assessed by imaging methods)[^219^](#_ENREF_219). Being less invasive than both laparoscopic and open surgery, core-needle biopsy is an excellent option in such patients in order to obtain the diagnosis and commence appropriate treatment[^88^](#_ENREF_88). In addition, pre-operative knowledge of the histological subtype of the tumor may contribute to the decision-making process when it is uncertain if complete cytoreduction can be achieved at upfront debulking surgery. In chemosensitive cancers, such as high-grade serous carcinomas, the strategy of neoadjuvant chemotherapy followed by interval debulking surgery can be used if there is uncertainty regarding the possibility of complete cytoreduction at upfront surgery. In less chemosensitive cancers, such as low-grade serous carcinomas or mucinous carcinomas, primary cytoreductive surgery is recommended even if uncertainty about achieving complete resection exists and a small residual tumor (<1 cm) is likely to remain[^88^](#_ENREF_88). Also, core-needle biopsy allows the collection of specimens from ovarian tumors with sonomorphology suggestive of secondary ovarian cancers or atypical metastatic spread, in order to exclude tumors of non-genital origin, or to confirm the diagnosis of pelvic and/or retroperitoneal masses of non-gynecologic/unknown origin (cancers of unknown primary), such as infiltrated lymph nodes and schwannomas[^220^](#_ENREF_220)^,^[^221^](#_ENREF_221). The 2024 ESMO-ESGO-ESP Consensus Conference on Ovarian Cancer recommends an adequate surgical or image-guided tumor biopsy specimen as the preferred sample for histological diagnosis and molecular tumor testing[^222^](#_ENREF_222). Similarly, the 2023 ESMO Clinical Practice Guideline on ovarian cancer recommends adequate tumor biopsy for histology and molecular testing if primary cytoreductive surgery is not feasible[^88^](#_ENREF_88). This is important because there may not be sufficient remaining viable tumor tissue for genetic testing during interval debulking surgery after neoadjuvant chemotherapy[^88^](#_ENREF_88). In line with European guidelines, the 2024 National Comprehensive Cancer Network guidelines recommend histologic confirmation of diagnosis and cancer subtype based on analysis of tumor tissue before neoadjuvant chemotherapy[^92^](#_ENREF_92). If tissue biopsy is not feasible, cytopathology from ascites or pleural effusion with a CA125/CEA ratio >25 can be used in combination with imaging data and discussion in a multidisciplinary team.

Similarly, if suspicious uterine mesenchymal tumors are detected on imaging, pre-operative transcervical core-needle biopsy may be indicated if fertility-sparing treatment is being considered instead of definitive treatment (total extrafascial hysterectomy without fragmentation)[^52^](#_ENREF_52).

Moreover, it can be useful in such situations when assessing tumors of the vagina and the cervix may be challenging or previous punch biopsy has led to inconclusive results[^155^](#_ENREF_155). Lia *et al.* studied core-needle biopsies using 14G needles to provide representative tissue samples for histotype, grading, and lympho-vascular space invasion[^155^](#_ENREF_155). In patients who develop metachronous endometrial cancer after definitive radiotherapy for cervical cancer, endometrial biopsy based on curettage, pipelle or hysteroscopy is often impossible due to the closed endocervical canal, and core-needle biopsy may be necessary.

In case of suspected disease recurrence or progression, biopsy can provide useful information about molecular alterations derived from next generation sequencing or immunohistochemistry and exclude metachronous malignancy[^11^](#_ENREF_11)^,^[^223^](#_ENREF_223).

Core-needle biopsy is recommended in the latest guidelines for the management of various gynecologic cancers to confirm the presence of metastases if equivocal distant lesions are detected on imaging[^87^](#_ENREF_87)^,^[^90^](#_ENREF_90).

Another indication emerging from oncological clinical trials is that of research biopsy, which is useful to understand the molecular biology of malignant disease and to develop targeted therapies against them[^224-226^](#_ENREF_224). Participants may have no direct benefits from the procedure, which raises ethical concerns because of the associated risks[^227^](#_ENREF_227). However, the procedure is usually well-tolerated, most patients appear to be willing to undergo serial biopsies (>80%) and the results may contribute to the discovery and development of novel therapies[^4^](#_ENREF_4).

The indications for core-needle biopsy are demonstrated in different clinical cases in Videoclip S4. The main indications and contraindications of core-needle biopsy are shown in Table 3.

**Table 3** Indications and contraindications for core-needle biopsy in gynecology/gynecological oncology

| *Indications for core-needle biopsy** | | |
| --- | --- | --- |
| Primary inoperable/non-resectable genital tumor (mostly ovarian/tubal cancer) | | |
| Cancer of unknown primary origin including metastases to genital organs (mostly secondary ovarian tumors) | | |
| Uterine mesenchymal tumor with atypical appearance on ultrasound or magnetic resonance imaging | | |
| Suspicious cervical or vaginal lesion (or, rarely, endometrial lesion if other methods of biopsy inapplicable) | | |
| Suspicion of recurrence of genital tumor | | |
| Research biopsy and molecular profiling (including *de novo* biopsy in case of disease progression or recurrence) | | |
| Staging purposes – inconclusive imaging findings | | |
| *Contraindications for core-needle biopsy*† | | |
| Thrombocytopenia | | |
| Biopsy with low procedure-related risk of bleeding when patient has elevated risk (PLT ≤ 30 × 10^9^/L) | |  |
| Biopsy with high procedure-related risk of bleeding when patient has elevated risk (PLT ≤ 50 × 10^9^/L) | |  |
| Antiplatelet therapy (ongoing)‡ | | |
| Coagulative disorder§ | | |
| History of procedural bleeding or known bleeding tendency | |  |
| Hemophilia | |  |
| Abnormal coagulation screening tests (prothrombin time or activated partial thromboplastin time) | | |
| Anticoagulation therapy (ongoing) | | |
| Vitamin K antagonists¶ | |  |
| Biopsy with low procedure-related risk of bleeding when patient has elevated risk (INR ≥ 2.0) |  |  |
| Biopsy with high procedure-related risk of bleeding when patient has elevated risk (INR ≥ 1.5) |  |  |
| Direct oral anticoagulants (dabigatran, rivaroxaban, apixaban, edoxaban)¶ | |  |
| Difficult access to the lesion | | |
| Risk of tumor spillage (upstaging of well-encapsulated mass due to iatrogenic intervention) | | |
| Absence of qualified operator, inadequate technical equipment, patient refusal or uncooperative patient** | | |

*In descending order based on frequency; core-needle biopsies for these indications are usually low-risk procedures, while percutaneous biopsies of abdominal visceral organs (e.g. liver, kidneys) and biopsies of hypervascular lesions (color score, 4) are considered high-risk procedures. †Predominantly relative contraindications that can be controlled by periprocedural management of thrombotic and bleeding risks, or considered on a case-by-case basis; there are no absolute contraindications specific to core-needle biopsy in gynecology/gynecologic oncology. ‡Risks and benefits of interruption of antiplatelet therapy should be considered, and periprocedural advice of treating specialist (hematologist/cardiologist/coagulation specialist) should be sought. In general, the following algorithm is recommended: if antiplatelet therapy can be interrupted safely, stop antiplatelet therapy 5 days prior to biopsy; if continued antiplatelet therapy is indicated, and patient is undergoing single antiplatelet therapy, low-risk procedures can be performed; if continued antiplatelet therapy is indicated, and patient is undergoing dual antiplatelet therapy, the risk of interrupting therapy is often high, and periprocedural management should be discussed with the treating specialist. See Table 5 for further details. §Periprocedural advice from hematologist should be sought. ¶See Table 5 for further details. **General absolute contraindications of any interventional procedure; in the event of patient refusal, always check the patient’s reasons carefully and explore whether there are concerns that can be addressed. INR, international normalized ratio; PLT, platelet count.

- - 1. Contraindications

Common contraindications for tissue-sampling techniques are coagulative disorders, such as hemophilia, thrombocytopenia, or the use of anticoagulation or antiplatelet therapy. Other contraindications include high risk of tumor spillage and the lack of a safe pathway to the target lesion (Table 3). All of the above contraindications are relative, rather than absolute, and have to be assessed on an individual basis for each patient. The only absolute contraindications are represented by patient refusal or inability to cooperate with and to obtain informed consent, that are considered general contraindications of any interventional procedure. The absence of experienced operators or inadequate equipment is also an obstacle to performing safe and successful procedures.

- - - 1. *Procedure-related risk*

The procedure-associated risk of bleeding is categorized by the Society of Interventional Radiology Consensus into low-risk and high-risk groups[^228^](#_ENREF_228)^,^[^229^](#_ENREF_229). High-risk procedures are defined as having a > 1.5% rate of major bleeding or 2-day risk of major bleeding of 2–4%, while low-risk procedures < 1.5% rate of major bleeding or 2-day risk of major bleeding of 0%–2%[^228^](#_ENREF_228)^,^[^230^](#_ENREF_230). High-risk procedures have a risk of bleeding that may be difficult to recognize or treat, such as bleeding within the peritoneal cavity, retroperitoneum, or parenchyma of visceral organs. However, the authors highlight that it is impossible to determine the specific risk associated with each procedure[^228^](#_ENREF_228)^,^[^231^](#_ENREF_231). The risk of the diagnostic procedure should not outweigh the potential benefits[^8^](#_ENREF_8)^,^[^9^](#_ENREF_9). Studies investigating core-needle biopsy in gynecology demonstrated a low rate of major bleeding complications (<1.5%) regardless of approach (Table S3)[^11^](#_ENREF_11)^,^[^19^](#_ENREF_19)^,^[^20^](#_ENREF_20). Obtaining biopsies from richly vascularized intraperitoneal tumors and/or abdominal visceral organs is associated with an increased risk of complications, in keeping with guidelines from the radiologic community[^228^](#_ENREF_228). In such situations, biopsies should be performed by the most experienced operators, and the use of a thinner core-needle (18G) should be considered to minimize tissue trauma (similar for renal or liver biopsies). Thinner needles should also be used in all patients assessed to be at elevated risk of bleeding[^20^](#_ENREF_20). If the lesion is accessible by two different approaches (transvaginal and percutaneous), the preferred option is transvaginal procedure due to better control of the needle, easy continuous visualization of the needle tip, higher resolution of the endovaginal probe and usually higher experience of the operator.

- - - 1. *Thrombocytopenia and coagulative disorders (including drug-induced)*

Patients with an elevated risk of bleeding include those with known bleeding disorders or prior bleeding complications, history of abnormal bleeding including heavy menstrual bleeding or postpartum hemorrhage, or those on anticoagulation or antiplatelet treatment[^71^](#_ENREF_71)^,^[^74^](#_ENREF_74)^,^[^75^](#_ENREF_75)^,^[^232^](#_ENREF_232). The preprocedural preparation should include appropriate counseling by a specialist to address the risks of bleeding and thromboembolism.

For procedures with a high risk of bleeding and/or patients at elevated general risk of bleeding, a routine screening coagulation panel is recommended, including hemoglobin, platelet count, prothrombin time (PT) and/or international normalized ratio (INR), and activated partial thromboplastin time (aPTT) (Table 4)[^228^](#_ENREF_228). In some patients, additional tests may be required, such as anti-Xa testing in patients receiving heparin and fibrinogen level in patients with cirrhosis. In patients with bleeding tendencies or with a history of severe bleeding, normal PT and aPTT cannot rule out all coagulation disorders, and specialized assessment is still recommended for additional laboratory testing[^228^](#_ENREF_228). Conversely, there is no recommendation to routinely assess platelet count, hemoglobin, INR or PT for patients at low bleeding risk undergoing low bleeding risk procedures[^228^](#_ENREF_228)^,^[^233^](#_ENREF_233). More detailed information, for when there is comorbidity, can be found in the consensus guidelines of the Society of Interventional Radiology[^228^](#_ENREF_228). In patient with elevated risk of bleeding, the laboratory thresholds for low-risk procedures are an INR <2.0 and platelet counts >30 × 10^9^/L[^228^](#_ENREF_228)^,^[^234-239^](#_ENREF_234). An INR <1.5 and a platelet count of >50 ×10^9^/L are the recommended cut-off values for procedures with high risk of bleeding [^160^](#_ENREF_160)^,^[^228^](#_ENREF_228)^,^[^240^](#_ENREF_240)^,^[^241^](#_ENREF_241).

**Table 4** Current recommendations for screening coagulation panel and thresholds to perform biopsy procedure, in order to minimize risk of major bleeding complications, according to procedure-related and patient-related risks of bleeding

| risk of bleeding | *Patient with low risk of bleeding* | *Patient with elevated risk of bleeding* |
| --- | --- | --- |
| Low procedure-related risk of bleeding* | PT/INR, aPTT, Hb, PLT not routinely recommended | PT/INR, aPTT, Hb, PLT should be considered†  Thresholds (correct to)‡:    INR < 2.0    PLT > 30 × 10^9^/L |
| High procedure-related risk of bleeding* | PT/INR, aPTT, Hb, PLT recommended routinely  Thresholds (correct to)‡:    INR < 1.5    PLT > 50 × 10^9^/L | PT/INR, aPTT, Hb, PLT recommended routinely†  Thresholds (correct to)‡:    INR < 1.5    PLT > 50 × 10^9^/L |

*Biopsies for indications specified in Table 3 are usually low-risk procedures, while percutaneous biopsies of abdominal visceral organs (e.g. liver, kidneys) or biopsy of hypervascular lesions (color score, 4) are considered high-risk procedures. †In addition, anti-Xa testing in patients receiving heparin and assessment of fibrinogen level in patients with cirrhosis. ‡Patient INR / PLT level should be corrected until threshold is met. aPTT, activated partial thromboplastin time; Hb, hemoglobin; INR, international normalized ratio; PLT, platelet count; PT, prothrombin time.

For patients on anticoagulation and/or antiplatelet therapy, the decision whether to withhold the therapeutic agents prior to biopsy and, if so, for what length of time depend on the patient’s overall clinical status and thromboembolic and bleeding risks and on the procedure-associated bleeding risk[^228^](#_ENREF_228)^,^[^242^](#_ENREF_242). If the procedural bleeding risk is low, most anticoagulant/antiplatelet drugs can be continued. In such a situation, the patient’s thromboembolic risk does not influence the clinical decision[^228^](#_ENREF_228)^,^[^243-246^](#_ENREF_243).

Conversely, for patients at elevated risk of bleeding and for procedures with high risk of bleeding, additional factors need to be considered, including the type of anticoagulant and antiplatelet agents used (Table 5)[^228^](#_ENREF_228)^,^[^229^](#_ENREF_229)^,^[^247-249^](#_ENREF_247). The final decision regarding the periprocedural management of anticoagulation, including the use of bridging therapy with low-molecular-weight heparin, should take into account and balance all the above risks[^250^](#_ENREF_250).

**Table 5** Recommendations for adjustment to specific anticoagulant and antiplatelet treatments in patients undergoing biopsy procedure

| *Anticoagulant or antiplatelet agent* | *Biopsy procedure with low risk of bleeding* | *Biopsy procedure with high risk of bleeding* |
| --- | --- | --- |
| Vitamin K antagonists  (warfarin, phenprocoumon, acenocoumarol) | Consider continuation, check INR to exclude supratherapeutic levels (target INR < 2.0); if withheld, to reinitiate on same day as procedure | Withhold for 5 days until target INR < 1.5; consider bridging only in selected cases with very high risk of thrombosis; resume on day after procedure in the absence of bleeding complications* |
| LMWH  (enoxaparin, nadroparin, tinzaparin, dalteparin) | Do not withhold, avoid peak plasma levels (perform biopsy procedure ≥ 6 h after last dose of LMWH) | Withhold for 12 h for prophylactic doses of LMWH, and 24 h for therapeutic doses of LMWH  Consider checking anti-Xa if renal function impaired |
| Direct oral anticoagulants  (dabigatran, rivaroxaban, apixaban, edoxaban)† | Do not withhold, avoid peak plasma levels (perform biopsy procedure ≥ 6 h after last dose of direct oral anticoagulant)  Skipping a single dose before and after the biopsy can be considered | Withhold 1–3 days before procedure (depending on agent and renal function‡)  Resume 1–2 days after procedure in the absence of bleeding complications |
| Aspirin | Do not withhold | Withhold 3–5 days before biopsy, then resume on day after procedure† |
| Ticagrelor | Do not withhold | Withhold 5 days before biopsy; resume on day after procedure  In patients with dual antiplatelet therapy (aspirin + ticagrelor), discuss with treating cardiologist/physician |
| Prasugrel | Do not withhold | Withhold 5 days before biopsy; resume on day after procedure  In patients with dual antiplatelet therapy (aspirin + prasugrel), discuss with treating cardiologist/physician |

*Warfarin will take 5–10 days to attain a full anticoagulant effect, as measured by an international normalized ratio (INR) > 2.0; therefore, consider use of a heparin ‘bridging therapy’ in patients at high risk of thromboembolism. †For details on specific anticoagulant agents refer to current guidelines[^228^](#_ENREF_228)^,^[^251^](#_ENREF_251). ‡Patients with impaired renal function may require longer; duration can be individualized. LMWH, low-molecular-weight heparin.

Considering percutaneous organ parenchymal biopsies as high risk in terms of major bleeding and thus following the relevant recommendations, Table 4 is in line with published studies. A prospective multicenter study of 8172 intra-abdominal core-needle biopsies under ultrasound guidance was published in 2015, with the majority of cases being liver biopsy. The rate of major bleeding complications was significantly higher in patients with an INR > 1.5 (p < 0.001) and in patients taking drugs that potentially interfere with platelet function or plasma coagulation (p<0.0333)[^160^](#_ENREF_160). A retrospective review of 30966 patients found a higher incidence of bleeding if aspirin was taken the day of the biopsy and within 3 days vs 10 days from the procedure, although the overall rate of bleeding complications was low (0.3%)[^241^](#_ENREF_241). The most commonly used imaging modality for guidance was ultrasound, (62.8%) and the most common needle caliber was 18G (74.5%). Another study involving 15181 percutaneous biopsies of solid organ parenchyma found that the incidence of bleeding complications did not differ between patients who took aspirin within 10 days of surgery (0.6%, 18/3195) and those who did not (0.4%, 52/11986)[^78^](#_ENREF_78). The mean and median biopsy needle size was 18G, and the majority of procedures were guided by ultrasound. The overall risk of bleeding in this study, including all patients, was very low for biopsies of solid parenchymal organs such as liver (0.5%), kidney (0.7%), lung (0.2%), pancreas (1.0%), and others (0.2%), even with anticoagulation or antiplatelet therapy. At present, there are still limited data to suggest that the ongoing use of single antiplatelet agents or anticoagulant therapy may be safe for some procedures with a high risk of bleeding, such as solid organ biopsies. Unless the biopsy is urgently needed, it is recommended not to administer this treatment before the procedure[^228^](#_ENREF_228).

- - - 1. *Difficult access to the lesion*

Although not an absolute contraindication, a lesion in a difficult to access location may result in the lack of a safe pathway for biopsy, for example due to proximity of great vessels in the retroperitoneum or close contact to parenchymatous organs. Such a scenario should be anticipated prior to the procedure, to allow selection of an appropriate thin core-needle (18G), assessment of an adequate route of biopsy and to ensure the availability of an experienced operator (level III, European Federation of Societies for Ultrasound in Medicine and Biology)[^252^](#_ENREF_252). A high level of operator experience (defined as >500 interventions before the study) was associated with a low risk of major bleeding in a large prospective multicenter study of 8172 ultrasound-guided intra-abdominal interventions[^160^](#_ENREF_160). In difficult to access tumor locations, biopsies can be safely performed through the urinary bladder or rectal wall (eventually with adequate antibiotic prophylaxis). Moreover, there are several reported cases of biopsies with 18G-20G core-needles using a trans-organ route (stomach, colon, small bowel, liver and spleen) with no occurrence of major complications[^175^](#_ENREF_175). In certain cases, alternative options such as CT-navigated biopsy, endoscopic ultrasound biopsy or diagnostic laparoscopy should be considered[^253^](#_ENREF_253).

- - - 1. *Tumor spillage (iatrogenic upstaging)*

Core-needle biopsy should not be performed if there is a risk of tumor cell spillage within the peritoneal cavity, especially in suspicious ovarian tumors with an intact capsule and no signs of extra-ovarian spread[^254^](#_ENREF_254)^,^[^255^](#_ENREF_255). Growing evidence suggests that even minor trauma can influence several processes that might promote postoperative metastatic spread and tumor recurrence[^256^](#_ENREF_256). In a retrospective analysis of 1545 patients with invasive ovarian cancers, Vergote *et al.* reported that capsule rupture is an indicator of reduced disease-free survival[^254^](#_ENREF_254).

- - - 1. *Other considerations*

For suspected lymphoma, the proportion of inconclusive samples after core-needle biopsy is higher compared to other indications. Based on the analysis of a large French study, core-needle biopsy obtained definitive diagnosis in 92.3% of all cases which was inferior to surgical excision (98.1%)[^257^](#_ENREF_257). Another study demonstrated a sensitivity of 89.0% to detect lymphoma in enlarged lymph nodes using core-needle biopsy, and histological subclassification necessary to start treatment was only possible in 85.0% of cases[^258^](#_ENREF_258). Although core-needle biopsy accurately diagnoses lymphoma in most cases, there is a risk of erroneous or inconclusive results. Therefore, surgical lymph node excision may be preferable if it is not associated with excessive morbidity.

For frail patients with poor performance status, the decision to do core-needle biopsy must be individualized. Core-needle biopsy can be used to avoid any surgery and initiate systemic treatment based on the histological results without significant delay from the first consultation, because biopsy can immediately follow the gynecologic examination and ultrasound performed for both diagnostic and staging purposes (as per the concept of a one-stop clinic). However, core-needle biopsy may not be indicated if it does not change management and the patient is not fit enough for oncologic treatment regardless[^259^](#_ENREF_259).

**Statement 12**: Core-needle biopsy should be performed if it is clinically meaningful for the patient’s management. Indications include: (1) to determine primary origin in patients with inoperable/non-resectable disease or unknown primary cancer; (2) to stage disease; (3) to identify residual or suspicious recurrent disease; (4) to establish the nature and histological diagnosis of suspicious uterine mesenchymal tumors; (5) to investigate suspicious cervical, vaginal or endometrial lesions and others; and (6) for targeted treatment including research purposes.

- Level of evidence: 2b
- Grade of statement: B
- Consensus: yes, 94% (*n* = 17); no, 0% (*n* = 0); abstain, 6% (*n* = 1)

**Statement 13**: There are no specific absolute contraindications to core-needle biopsy in gynecology/gynecological oncology. However, risks and benefits should be balanced, considering the patient’s comorbidities and medications, difficulty of access to the target lesion and risk of tumor spillage.

- Level of evidence: 4
- Grade of statement: C
- Consensus: yes, 100% (*n* = 18); no, 0% (*n* = 0); abstain, 0% (*n* = 0)

**Statement 14**: Any decision about periprocedural management should be based on a thorough assessment of the patient’s overall clinical status, including thromboembolic and bleeding risks and the procedure-associated risks.

- Level of evidence: 3b
- Grade of statement: C
- Consensus: yes, 100% (*n* = 18); no, 0% (*n* = 0); abstain, 0% (*n* = 0)

**Statement 15**: Clinicians performing a biopsy should be aware of potential complications and routinely implement strategies to avoid or minimize them.

- Level of evidence: 2b
- Grade of statement: B
- Consensus: yes, 100% (*n* = 18); no, 0% (*n* = 0); abstain, 0% (*n* = 0)

**Statement 16**: Regarding procedure-related risk of major bleeding, core-needle biopsies in gynecology/gynecological oncology are considered to be low risk (risk of major bleeding complication < 1.5%). Percutaneous biopsies of abdominal visceral organs (e.g. liver, kidneys) as well as any biopsy of hypervascular lesions (color score, 4) are considered high-risk procedures.

- Level of evidence: 4
- Grade of statement: C
- Consensus: yes, 100% (*n* = 18); no, 0% (*n* = 0); abstain, 0% (*n* = 0)

**Statement 17**: Regarding patient-related bleeding risk, women with coagulative disorders or on anticoagulative therapy and those with thrombocytopenia or on antiplatelet therapy are considered at elevated risk of major bleeding.

- Level of evidence: 4
- Grade of statement: C
- Consensus: yes, 100% (*n* = 18); no, 0% (*n* = 0); abstain, 0% (*n* = 0)

**Statement 18**: Withholding and restarting anticoagulant and/or antiplatelet drugs should be carried out according to recommendations of relevant specialists. The patient’s individual thromboembolic risk should also be taken into consideration.

- Level of evidence: 4
- Grade of statement: C
- Consensus: yes, 100% (*n* = 18); no, 0% (*n* = 0); abstain, 0% (*n* = 0)

**Statement 19**: For procedures with low risk of bleeding planned in patients with no or minimal bleeding risk factors, a screening coagulation panel is not required. These procedures can be performed by adequately trained sonographers (level II minimum).

- Level of evidence: 3a
- Grade of statement: C
- Consensus: yes, 94% (*n* = 17); no, 0% (*n* = 0); abstain, 6% (*n* = 1)

**Statement 20**: For procedures with high risk of bleeding or in patients at elevated risk of bleeding, a screening coagulation panel (platelet count, hemoglobin, PT/INR and aPTT) is routinely recommended. These procedures should be performed by expert sonographers (level III).

- Level of evidence: 4
- Grade of statement: C
- Consensus: yes, 100% (*n* = 18); no, 0% (*n* = 0); abstain, 0% (*n* = 0)

**Statement 21**: For all procedures with high risk of bleeding, recommended laboratory thresholds to minimize the risk of major bleeding complications are INR < 1.5 and PLT > 50 × 10^9^/L.

- Level of evidence: 4
- Grade of statement: C
- Consensus: yes, 94% (*n* = 17); no, 0% (*n* = 0); abstain, 6% (*n* = 1)

**Statement 22**: For patients at elevated risk of bleeding undergoing procedures with low risk of major bleeding complications, laboratory thresholds are INR < 2.0 and PLT > 30 × 10^9^/L. Antiplatelet therapy can continue. Anticoagulant therapy can continue in most cases.

- Level of evidence: 4
- Grade of statement: C
- Consensus: yes, 89% (*n* = 16); no, 0% (*n* = 0); abstain, 11% (*n* = 2)

## Technique

Ultrasound-guided biopsy should be performed only by a physician familiar with the indications, contraindications, limitations, typical findings and possible side effects of the procedure. The physician should be trained in gynecological oncology sonography as well as ultrasound-guided core-needle biopsy and related safety issues, and should undertake quality assurance and control measures routinely. The choice of approach to guide the needle’s path, caliber of the needle and penetration depth are dependent on the specific purpose and the safety of the procedure. The steps for performing ultrasound-guided biopsy are described in Table 6 and Videoclips S1-3.

**Table 6** Roadmap for performing ultrasound-guided core-needle biopsy (using automated device)

| *Step 1: Patient preparation* | |
| --- | --- |
| Ensure calm, comfortable environment and patient dignity.  Review indications for the procedure, multidisciplinary team recommendations and location for planned biopsy.  Obtain medical history, check for allergies, and identify risk factors for potential complications.  Explain procedure, risks and benefits to the patient and obtain informed consent. Ask for and address any fears or worries.  Assess risk of bleeding (see Table 4).  Seek specialist advice on withholding anticoagulant/antiplatelet treatment as necessary (see Table 5).  Offer use of oral analgesics such as 1 g paracetamol or oral non-steroidal anti-inflammatory drug, or analgesia suppository (TV/TR/TC procedure) if desired by the patient.  Administer prophylactic antibiotics if indicated (e.g. in case of passage of the needle through the rectal wall into the peritoneal cavity, immunocompromised patient, risk of infective endocarditis). |  |
| *Step 2: Selection of biopsy site and approach* | |
| Place patient in lithotomy (TV/TR/TC procedure) or supine (percutaneous) position and cover with a drape.  Perform an ultrasound examination to assess the feasibility of biopsy, identify target lesion, plan the access route to the target lesion and identify organs at risk of injury.  Use Doppler to identify viable tumor tissue, assess tumor vascularization and identify adjacent vascular structures.  Set up needle guidance line for TV and TR biopsies. Use of needle guide is optional for percutaneous biopsies.  Measure the penetration depth (distance from closest to farthest edge of the tumor to cover the tumor’s full thickness in the planned direction of biopsy) and set biopsy device accordingly. |  |
| *Step 3: Infection prevention and instrument preparation* | |
| Prior to use, clean and disinfect the ultrasound probes and machine.  Wash hands and perform antisepsis.  Prepare necessary instruments on instrument trolley (Figure 4).  Don sterile gloves.  Apply sterile gel to the transducer and enclose with a disposable transducer cover.  Apply local anesthetic gel to the tip of the covered ultrasound probe (for TV/TR procedure).  Affix needle guide to ultrasound probe (optional for percutaneous approach)  Attach biopsy needle to biopsy device and remove the spacer.  Charge device by pulling the lever and adjust penetration depth accordingly.  Test the firing of the device when in ‘FIRE’ mode then switch to ‘SAFE’ mode. |  |
| *Step 4: Performing the procedure (Videoclips S1–S3)* | |
| Monitor patient comfort throughout the procedure.  Disinfect the vagina or skin (TV and percutaneous procedures, respectively).  A rectal cleansing enema is optional (TR procedure).  Apply local anesthetic as indicated.  Percutaneous procedure: Under ultrasound guidance, administer local anesthetic injection to skin and abdominal wall in direction of the intended biopsy path up to the target tissue. The skin incision may be extended with a larger bore needle or scalpel for easier entry of the core needle if required.   TV/TR procedure: Insert the probe using a finger to guard the mucosal surface from the needle guide. Administration of local anesthetic along the needle trajectory using a long needle placed within the needle guide for anesthetic injection is optional.  TC procedure: A paracervical block can be used.  Hold the probe with one hand and introduce the needle with the needle guide with the other hand. For freehand percutaneous technique, insert needle along the longitudinal axis of the probe below the ultrasound beam.  Visualize needle tip continuously with ultrasound.  Align needle guidance line (if set up) with the lesion and insert needle to the nearest edge of the viable part of the lesion.  Switch the device to ‘FIRE’ mode and activate the trigger.  Place the specimen in formalin.  Repeat the procedure to obtain three biopsy cores. Between biopsy passes, the ultrasound probe should ideally be left in place, especially if the probe is inserted in the vagina or rectum.  After each sampling, an assistant helps to move the specimen to a formalin-filled container using either a needle stylet or a normal saline flush.  Ultrasound should be used to detect any signs of internal bleeding.  Percutaneous procedure: Apply pressure at the biopsy site for a few minutes and then cover with a sterile dressing.  TV procedure: Apply puncture site pressure using a gauze swab in the vagina for 1–5 min after the puncture to stop vaginal bleeding if necessary. |  |
| *Step 5: Postprocedural requirements* | |
| Give the patient time to sit up and dress, and assess her condition.  Inform the patient how and when the results of biopsy will be communicated, and provide written information about signs of potential complications and emergency contacts. Inform the patient of the use of postprocedural oral analgesia, if required.  In uncomplicated low-risk procedures, no postprocedural monitoring is required.  Label specimen container with patient identifiers.  Fill pathology request form with relevant information:   - - Patient identifiers - - Clinical data, radiological data and patient history - - Details of requesting doctor - - Biopsy site, type of specimen and fixative used - - Clinical impression and differential diagnosis - - Previous histopathological findings (if any) - - Specific requests to the pathologist according to clinical need (immunohistochemistry, molecular analysis or other processing) - Attach pathology request form to specimen container. |  |

This table was formulated using both data from the literature and by consensus of the study authors[^104^](#_ENREF_104)^,^[^242^](#_ENREF_242)^,^[^260-263^](#_ENREF_260). TC, transcervical; TR, transrectal approach; TV, transvaginal approach.

The necessary instruments for core-needle biopsy are shown in Figure 4.


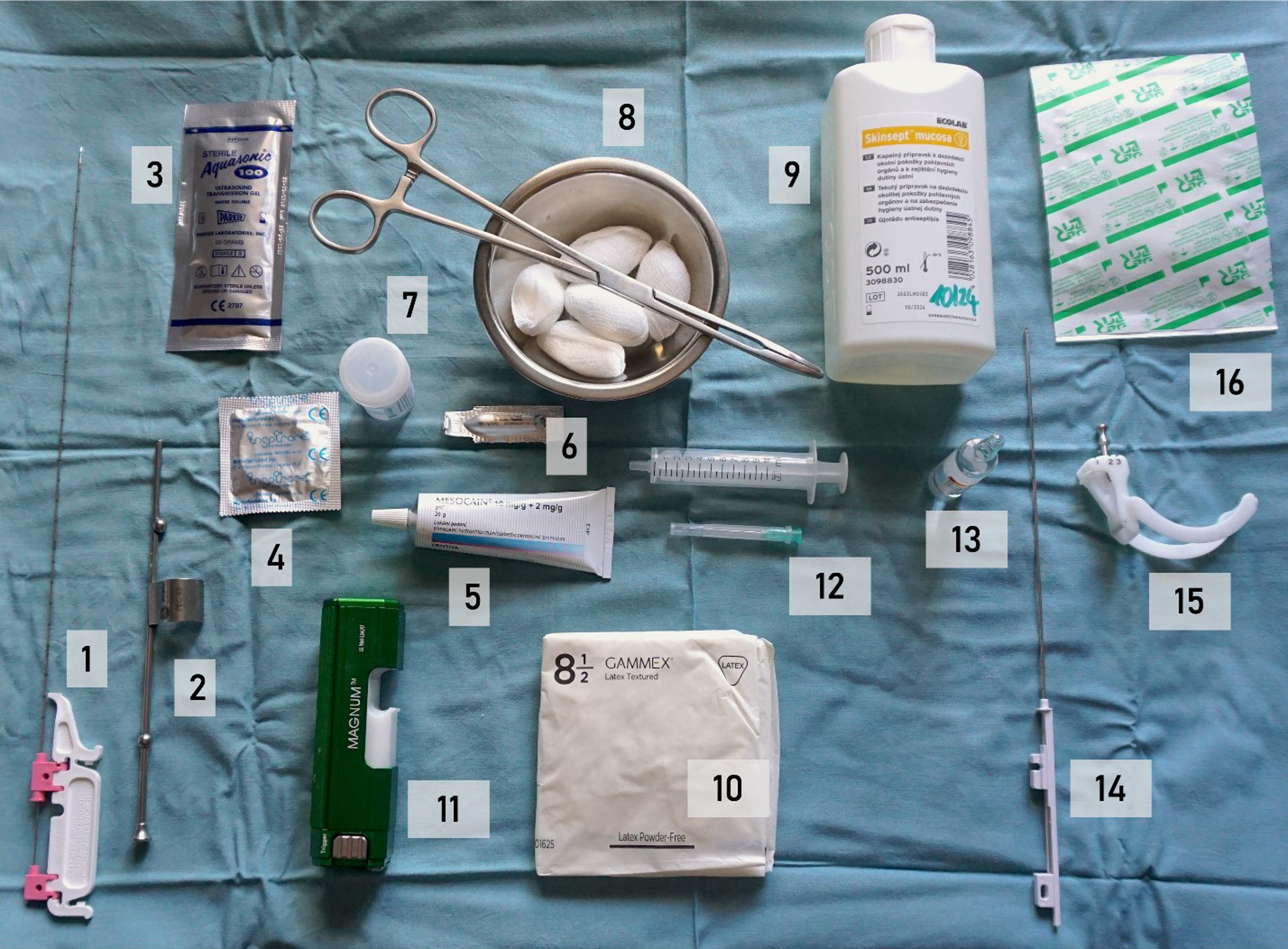


**Figure 4** Instruments needed for core-needle biopsy: (1) biopsy needle (30 cm/18 G); (2) needle guide for endocavitary probe; (3) sterile gel; (4) protective probe cover; (5) anesthetic gel; (6) analgesia suppository; (7) labeled specimen container; (8) basin, tongs and swabs for disinfection; (9) antiseptic cleaning agent; (10) sterile gloves; (11) automated core-needle device; (12) needle and syringe for application of local anesthetic; (13) local anesthetic; (14) biopsy needle (20 cm/16 G); (15) needle guide for convex probe (optional); (16) wound covering. For transvaginal/transrectal biopsy procedure, instruments 1–11 are needed; for percutaneous biopsy procedure, instruments 3 and 7–16 are needed.

- - 1. Preprocedural preparation

The preprocedural preparation includes providing the patient with relevant information, obtaining informed consent, and taking necessary precautions to minimize procedure- and patient-related (medications, comorbidities) complications.

- - - 1. *Patient selection*

Before starting the biopsy, the physician should carefully check the indications, perform an ultrasound examination to identify the target lesion and possible route of access and evaluate the procedure-related risks. At the same time, the patient's medical history should be taken to identify any clinical conditions that may increase the risk of complications, as indicated (Table 4). The patient should also be asked about the history of any allergy.

- - - 1. *Patient counselling*

When all necessary information is obtained, the physician should explain the purpose of obtaining a biopsy and how it would guide her management, how the procedure would be performed, how long it would take, the likely degree of discomfort and the measures which would be taken to minimize it. Possible risks of the procedure should also be explained including the risk of infection, bleeding and injury to the internal organs. Information about possible alternative procedures should be given. The patient should also be told how long it would take for the sample to be analyzed and how the results of histological examination would be communicated. The possibility of an inconclusive result due to insufficient material, necrotic lesion or not sampling the area containing malignancy should be explained, and the patient should be aware that in such situations, a repeat biopsy would be required. Ideally, written consent should be obtained for all invasive procedures although this may not be required in all countries and depends on the national legislation. For clinical research biopsies, formal written consent to participate in the study is required, and the written patient information and consent form should be approved by the institutional committee for ethics in research[^264^](#_ENREF_264).

- - - 1. *Assessment of biopsy risk and additional preparation*

Ultrasound-guided core-needle biopsy is considered to have a low risk of major bleeding complications (<1.5%)[^7^](#_ENREF_7)^,^[^12-14^](#_ENREF_12)^,^[^40^](#_ENREF_40)^,^[^100^](#_ENREF_100). In a patient with low bleeding risk based on her medical history, no special preparation, such as fasting, use of laxatives and anti-flatulent medication, or screening coagulation panel is required.

In procedures with high risk of major bleeding complications (>1.5%) and/or in a patient with a high bleeding risk, a screening coagulation panel is indicated (Table 4)[^228^](#_ENREF_228). Withholding and restarting anticoagulant and/or antiplatelet drugs should be in accordance with the recommendations of relevant specialists. Patient’s individual thrombotic risk must be taken into consideration[^232^](#_ENREF_232). In patients with high thromboembolic risk, a bridging plan with low molecular heparin should be devised in consultation with a hematologist. Such patients include those with atrial fibrillation, venous thromboembolism, previous stroke, mechanical heart valves or stents who are usually on long-term anticoagulation[^228^](#_ENREF_228). The decision whether to bridge therapy with heparin should take into consideration the benefits of bridging versus non-bridging, and it is emphasized that bridging may not eliminate the risk of excessive bleeding[^232^](#_ENREF_232)^,^[^265-268^](#_ENREF_265). There is no strong evidence available to guide clinicians when deciding on potential benefits of antibiotic prophylaxis for tumor biopsy[^261^](#_ENREF_261). The risk of infection with transabdominal and transvaginal procedures is low (≤1%) and the decision whether to prescribe antibiotic prophylaxis should be at the discretion of the operator[^269^](#_ENREF_269)^,^[^270^](#_ENREF_270). There are special situations where empirical antibiotic prophylaxis may be considered, such as transrectal biopsies and biopsies in immunocompromised or diabetic patients[^262^](#_ENREF_262)^,^[^271^](#_ENREF_271)^,^[^272^](#_ENREF_272). However, antibiotic prophylaxis is not recommended for image-guided biopsy of solid rectal and perirectal lesions when the needle does not breach the peritoneal cavity, as the rate of infections seems as low as 2%[^164^](#_ENREF_164)^,^[^273^](#_ENREF_273). If there are signs of infection at the potential biopsy site, the procedure should be postponed until the infection has been successfully treated. Antibiotic administration has been suggested for fine-needle aspirations of cystic lesions[^25^](#_ENREF_25)^,^[^273^](#_ENREF_273). No data support the use of specific agents, and a single-dose antibiotic prophylaxis is acceptable. Amoxicillin with clavulanic acid or cefoxitin are often the first-line options, but in patients who are allergic to these antibiotics, alternative preparations should be used.

The procedure should be performed in a calm and comfortable environment, taking care to provide adequate psychological support to the patient throughout[^4^](#_ENREF_4). Attention should be paid to the patient's dignity and privacy, and measures should be taken to minimize pain and anxiety associated with the procedure, highlighting the importance of the communication between patient and operator to ensure the overall satisfaction[^274^](#_ENREF_274). The patients should be advised that they may take over-the-counter painkillers such as oral paracetamol 1000mg 30-60 minutes prior to the procedure. Although the majority of biopsies are quick and painless, some women may experience discomfort and moderate to severe pain[^4^](#_ENREF_4). Patients who are particularly anxious and those who are unable to tolerate internal ultrasound examination are not good candidates for outpatient biopsy and should be offered conscious sedation or general anesthesia as inpatient.

The biopsy procedure can either immediately follow the diagnostic ultrasound or be scheduled at a later time based on clinical needs and service organization. In a study examining patient satisfaction and anxiety levels in women undergoing core-needle breast biopsy in the same-day vs later-day groups, researchers found higher levels of anxiety in patients undergoing same-day biopsies, although satisfaction was higher[^274^](#_ENREF_274). A study on thyroid fine-needle aspiration demonstrated that the use of classical music can significantly reduce anxiety[^275^](#_ENREF_275)^,^[^276^](#_ENREF_276).

- - 1. Ultrasound-guided biopsy

One of the most important points for a successful ultrasound-guided biopsy is choosing the best path to the target lesion with the lowest risk. Core-needle biopsy usually takes between 5 and 15 minutes to complete. The biopsy can be performed in a dedicated outpatient or inpatient operating theatre or on the examination couch in the ultrasound room. If multiple consecutive procedures are planned, a gynecological examination table should be used to ensure patient and operator comfort. The steps for performing ultrasound-guided biopsy are summarized in Table 6.

- - - 1. *Select the biopsy site and approach*

First, a systematic pelvic and/or abdominal ultrasound examination should be performed. The lesion of interest should be identified and measured in three perpendicular planes, and its relationship with visceral organs and blood vessels and other critical structures should be assessed. The use of Doppler ultrasonography facilitates the assessment of the lesion vascularization and the location of nearby vascular structures. The interventional access pathway with the lowest risk should be identified, making sure that the lesion can be accessed without the risk of injury to any adjacent organs. Although not always possible, the shortest route is typically preferred. If any “risky” structure is present on the anticipated pathway, then a longer but still safe route may be chosen. The appropriate transducer and approach for biopsy guidance should be determined. The digitalization of ultrasound machines has made it possible to plan the needle path with a virtual (electronic) line on the screen, ensuring that the line reaches the target without crossing vital structures such as large vessels. The distance between the lesion and the planned needle entry point is measured and documented so that the correct needle length can be selected.

- - - 1. *Prepare the biopsy instruments*

The equipment required for core-needle biopsy is shown in Figure 4. All instruments should be laid out on a sterile trolley. The length of the biopsy needle will depend on the planned biopsy route: 15-20 cm for percutaneous approach and 30-35 cm for transvaginal/transrectal approach. The core-needle biopsy procedure obtains a cylinder from the target tissue of variable length and thickness. The length of the core depends on selected penetration depth on the respective biopsy instrument in relation to location and depth of the lesion. The thickness of the core (1.3-2.1 mm wide, 14-18 G) depends on the diameter (caliber) of the needle. The needle gauge should be chosen based on the risk of bleeding balanced against the diagnostic yield. The higher the risk of bleeding, the thinner the needle should be. The specimen may be taken with a cutting needle (for core-needle biopsy – e.g., side-cut type or end-cut type) (Figure S2) or aspiration needle (for fine-needle aspiration – e.g Menghini or Chiba type) (Figure S5), which have specific characteristics and needle tip shape. The side-cutting needles (tru-cut type) are available with automated, semi-automated or manual firing modes. Automated and semi-automated devices are both suitable for performing core-needle biopsies. The choice between these devices depends on the operator's preference, the patient's characteristics, and their availability. With automated devices, once triggered, the stylet and cannula both advance rapidly, penetrating the tissue and obtaining a biopsy sample. With semi-automated device, the stylet is positioned into the target, once triggered, the cannula rapidly advances to cut the sample. Generally, semi-automated devices offer greater control during the procedure; however, it may be more difficult to manually insert the stylet into harder lesions. Regardless of device chosen, the selected single-use disposable needle is inserted within a device, and penetration depth is set (Figure S7). The instrument is tested, and the trigger button is put on safety mode.

The ultrasound probe is covered with gel and a sterile, disposable protective cover for both, endocavitary but also for convex and linear probes. The needle guide is attached to the ultrasound probe in the transvaginal and transrectal approaches. It is optional in the percutaneous approach. For less experienced operators, a steering device may improve operator confidence but may be compromised by less flexibility for needle manipulation during insertion. Thus, experienced operators often use the free-hand technique, especially to target difficult-to-access lesions (Figure S7).


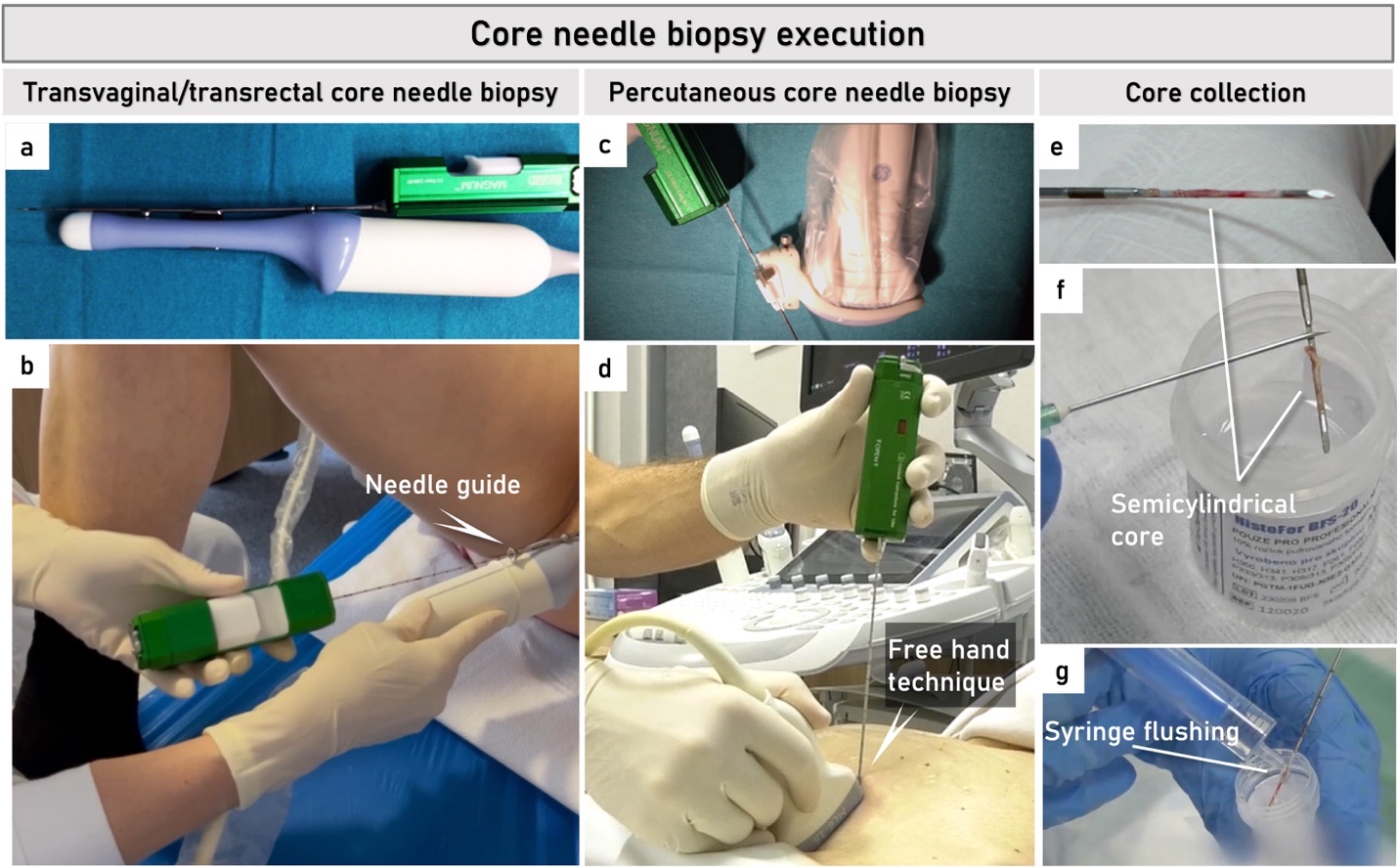


**Figure S7.** Different approaches for performing a core-needle biopsy. In (a) the biopsy device with biopsy needle is inserted into a disposable metal needle guide attached to the endocavitary probe during a transvaginal or transrectal biopsy; in (b) this technique is demonstrated with the patient in lithotomy position. In (c) a plastic needle guide is attached to the convex array probe with a channel for the needle, which may be positioned at different angles (depending on the system). In (d) the free hand technique for the percutaneous transabdominal approach is demonstrated on a patient. In (e) the needle tip is shown after firing the device with penetration depth pre-selected; the tissue core is retrieved within the hollow part of the needle and placed in a container with formalin (f), with the use of a syringe to flush the tissue out (g). A portion of the fixative is taken from the container into the syringe to be used for flushing. An alternative method is to flush the specimen with saline solution, after which the tissue must then be taken from the saline solution and placed in fixative.

- - - 1. *Administration of local anesthesia and sedation*

Taking into account patient comfort and safety, the administration of local anesthesia and sedation may be indicated in certain situations where the procedure is expected to take longer, be more painful, be complicated, or when the patient is especially anxious. Nakai *et al.* reported that a longer operative time and a higher anxiety can increase the pain for patients undergoing the procedure without anesthesia[^47^](#_ENREF_47). Sedation comprises a continuum from minimal sedation (anxiolytics) to general anesthesia. Administration of drugs for sedation should be reserved for personnel with the necessary knowledge and experience according to national legal regulations.

- - - - 1. Transvaginal approach

For the transvaginal approach, in patients with severe vaginal atrophy and in those with history of brachytherapy it is often helpful to apply local anesthetic gel five minutes prior to starting the procedure. If the insertion of biopsy needle is anticipated to be painful or difficult, 1% lidocaine could also be injected. After application of local anesthetic gel into the vagina (for transvaginal approach) or rectum (for transrectal approach), insert a lubricated probe with the needle guide attached using a finger to guard the mucosal surface from the sharp edges of the guide. After the needle route to the target lesion is selected, a 10ml syringe filled with local anesthetic (e.g., Lidocaine 1%) is attached to a long 20G or 18G needle. The needle is then placed into the needle guide and slowly advanced into the vaginal wall which is infiltrated with local anesthetic. The needle is advanced in short 1-2mm steps and local anesthetic is injected into all tissue layers along the needle trajectory until the target lesion is reached. The needle is then removed, and core-needle biopsy is carried out as described below. However, data regarding the value of routine local anesthetic use are limited. Zikan *et al.* reported a successful tissue sampling in 131 patients, with no patients reporting significant pain during the transvaginal biopsy without local anesthesia[^7^](#_ENREF_7). Similar results have been reported by Verschuere *et al.* who did not record any cases of severe pain in a series of 155 women referred to transvaginal core-needle biopsy without local anesthesia[^97^](#_ENREF_97). A recent systematic review of randomized controlled trials on pain relief during oocyte retrieval found a broad heterogeneity in the studied outcome measures which hindered the generalizability and comparability of the evidence[^277^](#_ENREF_277). The authors did find that women who were given placebo experienced more pain that those who had conscious sedation. They also reported that the pain levels were significantly lower in patients who were given a para-cervical block compared to local anesthetic vaginal gel. Of note, despite some similarities between oocyte collection and tumor biopsy, these two procedures differ in many important aspects. Oocyte collection involves multiple passages of the aspiration needle through the vaginal wall, parietal, and visceral peritoneum into the healthy ovary. In contrast, tumor biopsy typically requires only three passages of the needle into a neoplastic lesion which has no innervation. On the other hand, tumor biopsies are often done in older women with atrophic vaginal mucosa which may be more sensitive to pain compared to younger women of reproductive age having oocyte collection. Given these differences, it is important to exercise caution when extrapolating information about pain associated with oocyte collection to biopsies of pelvic tumors[^278^](#_ENREF_278)^,^[^279^](#_ENREF_279). In the absence of conclusive evidence, the routine use of local anesthetic cannot be recommended, although we suggest that local anesthetic gel should be offered to all women who find routine transvaginal ultrasound examination uncomfortable.

- - - - 1. Transrectal approach

For the transrectal approach, the Korean Society of Urogenital Radiology recommends the application of intrarectal lidocaine gel to reduce the pain caused by the distension of the anal canal[^280^](#_ENREF_280). However, the superiority of anesthetic gel compared to conventional ultrasound gel is not clear. The technique of administration of local anesthesia, if required, is similar to the transvaginal approach and is described above.

- - - - 1. Transcervical approach

Kawamura *et al.* reported on transcervical core-needle biopsy with transabdominal ultrasound guidance performed in 435 patients with uterine myoma-like tumors, and the majority underwent the biopsy without anesthesia[^52^](#_ENREF_52). However, a paracervical block with 1% lidocaine (6–18 mL), and/or venous injection of both pentazocine (15–30 mg) and diazepam (10 mg) were given if needed[^52^](#_ENREF_52). Stukan *et al.* in a recent feasibility study described transcervical (trans-uterine cavity) core-needle biopsy performed under short intravenous anesthesia, however, this group of patients also underwent operative hysteroscopy or cervical canal dilatation and curettage in the same setting[^51^](#_ENREF_51). From the literature about diagnostic hysteroscopy, Bettocchi et al. reported their experience with 31000 outpatient hysteroscopies which were all performed without any anesthesia. The procedure was successfully completed in 94% of cases and was well tolerated by patients[^281^](#_ENREF_281). Other studies on hysteroscopic biopsy have shown that the use of paracervical block and lidocaine gel can both aid in pain management and make the procedure more comfortable for women[^25^](#_ENREF_25)^,^[^282^](#_ENREF_282)^,^[^283^](#_ENREF_283). Taking these findings from hysteroscopy into account, we suggest that pain management should be individualized according to the individual patient’s needs and expectations.

- - - - 1. Percutaneous approach

For the percutaneous approach, after skin preparation with an antiseptic solution, the majority of clinicians perform infiltration of the abdominal wall with local anesthetic. Lidocaine is the most frequently used agent. Due to its rapid onset of action (1 to 5 minutes), it can be injected immediately before performing the procedure[^284^](#_ENREF_284). It is available in different concentrations (from 0.25 % to 5%). Typically, 10 mL of 1% lidocaine provides effective local anesthesia lasting approximately 2h without epinephrine, and 3 – 5h with epinephrine. Adding epinephrine to lidocaine solutions causes local vasoconstriction and increases the duration of analgesia and may also reduce post-procedural bleeding from the biopsy site.

The dose of local anesthetic depends on the location and depth of the intended target. For example, in liver biopsy, the needle is first advanced to the organ capsule and then to the target. It is recommended to administer local anesthesia under ultrasound guidance to ensure adequate analgesia along the planned needle path.

- - - 1. *Infection prevention*

Ultrasound-guided biopsy is carried out in a clean, though not necessarily sterile, manner. Basic disinfection procedures are sufficient as ultrasound-guided biopsy in gynecology/gynecologic oncology is not associated with an increased risk of infection. The operator should perform hand antisepsis and then wear sterile gloves. Ultrasound transducers used in image-guided interventional procedures are generally classified as semi-critical items (objects that come into contact with mucous membranes or skin that is not intact). For probe handling the standard operating procedure is followed. After the previous ultrasound examination, any residual ultrasound gel should be carefully removed from the probe with a disposable towel and the transducer cable wiped with a towel moistened with cleanser, followed by disinfection with a virucidal agent. Finally, a sterile disposable cover is used for endocavitary transducers, but can also be beneficial for linear and convex transducers.

The main principles pertaining to clean-contaminated procedures should be observed while performing an invasive procedure to minimize the risk of infection. For the transvaginal approach, cleansing of the vagina is performed using chlorhexidine or octenidin dihydrochloride soaked sponges held on forceps. For transrectal biopsy, the use of a cleansing enema with octenidin dihydrochloride before the procedure can be considered[^285^](#_ENREF_285). For the percutaneous approach, the skin is disinfected. Alcohol-based skin preparations using either chlorhexidine or iodine have been shown to be equally effective[^286^](#_ENREF_286)^,^[^287^](#_ENREF_287). It is crucial to ensure the needle tip remains sterile during the procedure, coming into contact with only the skin, cervix or vaginal mucosa which should have been previously disinfected.

Whenever available, disposable, single-use biopsy instruments are preferred. Otherwise, the biopsy instruments should be submitted to machine decontamination (cleaning and disinfection) followed by sterilization as defined by standard operating instructions. The ultrasound transducer should be adequately cleaned after every examination and biopsy procedure as described above.

- - - 1. *Taking the biopsy*

Ultrasound-guided biopsy is a one-person procedure, although an assistant is required for ultrasound guidance in transcervical biopsies and for transferring biopsy samples to the biopsy container. The operator holds the probe with one hand and introduces the needle inserted in the biopsy device with the other (Figure S7). Because the biopsy device is lightweight, the same hand also energizes and activates the biopsy device. The tip of the biopsy needle must be continuously visualized during the whole procedure to achieve optimal sampling and ensure patient safety (Figure S8). Real-time visualization of the needle tip is possible using ultrasound due to the reflection from the metal in the needle. The intensity of the display of echoes from the ‘needle plane’ will depend on the needle size, the scanning depth, angulation, and the ultrasound system used.


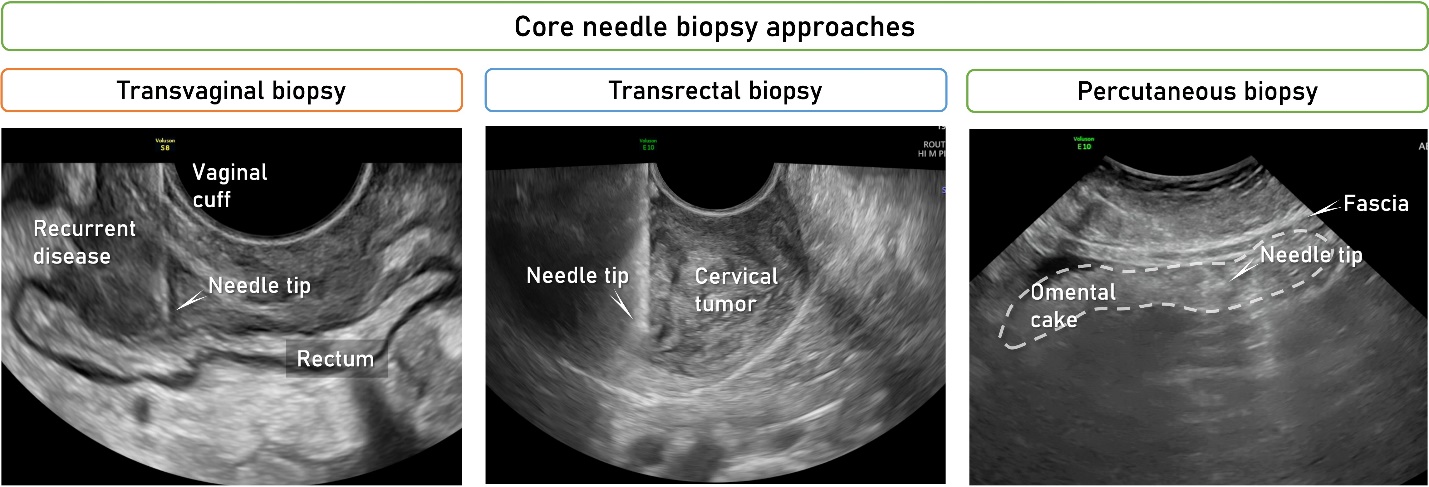


**Figure S8.** Core-needle biopsy approaches in the gynecologic oncology practice. Via transvaginal route (left) an ultrasound-guided core-needle biopsy is performed in a postmenopausal woman treated for ovarian cancer FIGO stage IIIC, referred for suspected recurrent tumor (disseminated carcinomatosis). Pathology revealed the recurrence of the high-grade serous carcinoma. By transrectal biopsy (middle), woman in her 40s, virgo, with abnormal uterine bleeding, and sideropenic anemia was diagnosed with a benign uterine mesenchymal tumor protruding through the cervical os into the vagina. Using the convex probe, the percutaneous core-needle biopsy (right) of the “omental cake” in a postmenopausal woman with abdominal carcinomatosis and previous breast cancer; pathology revealed the recurrence of the breast ductal cancer.

Using color/power Doppler the operator can identify and avoid large vessels. In addition, the use of Doppler examination helps to define a viable portion of the lesion without necrotic/cystic tissue, thus minimizing the risk of obtaining an inconclusive sample. When the needle is in a correct position and the penetration depth is checked, the device is activated (from safety to fire mode), allowing it to fire after pressing the trigger button. An image or videoclip should be taken to document the position of the needle within the lesion. For retrieval of the biopsy sample from the needle stylet, the assistant may flush the needle with 0.9% normal saline solution to avoid manipulation or use a small needle to scratch the core into a labeled container with 4-10% buffered formalin (Figure S7).

To ensure that the sample is suitable for histological examination, it is recommended to take at least 2-3 cores, each 18G or thicker. The collected tissue should be stored and processed, as described in Table 2[^288^](#_ENREF_288). At the end of the procedure, ultrasound imaging should be used to detect any signs of internal bleeding (e.g. ’fountain sign’, i.e. turbulent flow from the tumor itself or ‘patent tract sign’, i.e. visualization of flow along the needle tract in immediate post-biopsy Doppler)[^7^](#_ENREF_7)^,^[^289^](#_ENREF_289). Mild internal bleeding from the biopsy site usually resolves spontaneously within 5 minutes. Control of external bleeding from the biopsy site in the vagina (due to puncture through the vaginal wall) is possible using tampon forceps with a disinfectant swab, which is inserted into the vagina after the procedure. Applying a local hemostatic tamponade and prolonging pressure is very effective in cases of prolonged bleeding. The characteristics of different approaches are introduced in the following text and summarized in Table S5.

**Table S5.** Characteristics of the different approaches for core-needle biopsy in gynecological oncology[^4^](#_ENREF_4)^,^[^30^](#_ENREF_30)^,^[^198^](#_ENREF_198)

| *Ultrasound-guided core-needle biopsy* | | | | | |
| --- | --- | --- | --- | --- | --- |
| Approach | Transvaginal | Transrectal | Percutaneous | Transcervical |  |
| Probe | 5-9 MHz endocavitary (covered with sterile probe cover and needle-guide attached) | 5-9 MHz endocavitary (covered with sterile probe cover and needle-guide attached) | 3-5 MHz convex  (+/- needle guide) for transabdominal  4-10 MHz linear array probe for peripheral lymph node | 5-9 MHz endocavitary (covered with sterile probe cover) to be inserted transrectally***  or  3-5 MHz convex*** |  |
| Needle | ≤18G | ≤18G | ≤18G | ≤18G |  |
| Needle length | 30-35 cm | 30-35 cm | 15-20 cm | 20-30cm  With a plastic cover or stainless-steel pipe (to guide needle through cervical canal) |  |
| Position | Lithotomy | Lithotomy | Supine | Lithotomy |  |
| Local anesthesic | Optional | Optional | 10-20 ml of 1% lidocaine | Optional  Paracervical block with 1% lidocaine (6–18 mL)  or sedation / short intravenous anesthesia if needed |  |
| Disinfection before the procedure | Yes | Yes^†^ | Yes | Yes |  |
| Core length | ≥10 mm | ≥10 mm | ≥10 mm | ≥10 mm |  |
| Number of core samples | 2-3 | 2-3 | 2-3 | 3 |  |
| Antibiotic prophylaxis | May be considered* | May be considered* | No | No |  |
| Monitoring of the patients | Optional, 30 minutes** | Optional, 30 minutes** | Optional, 30 minutes** | Optional, 30 minutes** |  |

*e.g. Amoxicillin/clavulanate or clindamycin single dose when creating a continuity with urinary tract during transvaginal approach or peritoneum during transrectal approach.

^†^Optionally performed

**at least 1 hour if high risk of major bleeding complications

*** Note that the transrectally placed endocavitary probe or alternatively transabdominally placed convex probe are used to guide the procedure and the needle guide should not be used

- - - - 1. Transvaginal approach

The transvaginal approach uses a 5-9 MHz endocavitary probe with a needle guide attached. The needle path corresponds to the path of the ultrasound beam and can be planned using a virtual (electronic) line on the screen. The procedure is very similar to oocyte retrieval, with an ultrasound imaging guiding line displayed on the screen. It is performed with the patient in gynecologic position and after vaginal disinfection. The use of local anesthesia has already been discussed above. Ideally, the target mass should be within a few centimeters from the vaginal cuff and without the interposition of other structures, such as bowel, bladder, ureters, and large vessels. The transvaginal guided core-needle biopsy is presented in Videoclip S1.

- - - - 1. Transrectal approach

In some cases, a transrectal approach is preferred, for example due to the inaccessibility of the lesion via the transvaginal approach (e.g. after brachytherapy, colpectomy, virgo patient etc) or for a better visualization of the lesion[^50^](#_ENREF_50)^,^[^77^](#_ENREF_77)^,^[^290^](#_ENREF_290). The transrectal approach can be performed with the same patient position and using the same endocavitary probe as for the transvaginal approach (Videoclip S2). The use of cleansing enema and local anesthesia has already been discussed above. When the transrectal approach is used, the administration of prophylactic antibiotics may be considered[^291^](#_ENREF_291)^,^[^292^](#_ENREF_292).

- - - - 1. Trancervical approach

The transcervical approach can be used in patients with suspected uterine (e.g. mesenchymal) tumors in whom hysterectomy is not feasible or where fertility preservation is desired. A negative urine pregnancy test should be documented prior to any transcervical procedure. It is considered an “in-organ” biopsy, which is important if sarcoma is suspected to minimize the risk of intraperitoneal spread of malignant cells or biopsy tract tumor seeding due to the biopsy[^51^](#_ENREF_51)^,^[^54^](#_ENREF_54). The transcervical biopsy can be performed by one operator with the patient in gynecologic position, with ultrasound guidance performed by a second operator. A videoclip presenting the procedures has been recently published[^51^](#_ENREF_51)^,^[^293^](#_ENREF_293). After vaginal cleansing, it is optional to first perform a diagnostic hysteroscopy to visualize the uterine cavity and the position of the uterine corpus. A speculum is used to visualize the cervical os. During insertion, the biopsy needle should remain covered with its plastic sheath, which should be trimmed to the needle's length while in safe position to facilitate its passing through the cervical canal. The needle with its sheath is then inserted into the uterine cavity via the cervical canal[^51^](#_ENREF_51)^,^[^52^](#_ENREF_52). Alternatively, a stainless-steel hollow cannula can be first inserted for subsequent safe insertion of the biopsy needle[^52^](#_ENREF_52). The procedure can be guided with either transrectal or transabdominal ultrasound performed by an assistant. The device can then be fired while under continuous ultrasound guidance. Difficulties may be encountered in obese patients using the transabdominal approach for guidance, or if there is acoustic shadowing caused by tumor, a fundal lesion in an enlarged uterus or a laterally localized uterine mesenchymal tumor[^51^](#_ENREF_51). There may also be a lack of coordination between the performing physician and assistant. Additionally, there may be lesions that are difficult to target with this approach, such as myometrial tumors localized in a parallel plane to the line of the needle inserted via the cervical canal. It is important to recognize that the needle tract cannot be modified much once inserted because the angling of the needle is limited by the cervical canal[^51^](#_ENREF_51). A greater range of motion can be achieved by applying pressure on the uterine corpus with the transabdominal ultrasound probe with simultaneous visualization of the needle. However, this maneuver may be difficult in patients who are obese or do not have an enlarged uterus.

- - - - 1. Percutaneous approach

For the percutaneous (transabdominal) approach, a 3-5 MHz convex probe is used to identify the lesion and biopsy is obtained using a standard caliber needle 18G or wider/15-20 cm length. The biopsy can be performed with a needle guide attached to the ultrasound probe or as a free hand procedure. The procedure is demonstrated in Videoclip S3.

In the percutaneous needle-guided approach, the needle is inserted parallel to the transducer in the scanning plane (Figure S7), and the needle path corresponds to the path of the ultrasound beam. Using a free-hand technique, the needle may be inserted parallel or perpendicular to the transducer (‘off the scanning plane’). For less experienced operators, it is recommended to use the needle-guided approach, as this has been shown to be faster than the free-hand approach[^56^](#_ENREF_56)^,^[^294^](#_ENREF_294). A significant reduction in time required to obtain proper sampling is of particular importance when patient cooperation (suspension of respiration) is necessary. The free-hand technique has a learning curve but allows more versatility. Thus, experienced operators may use the free-hand technique for more difficult biopsies (less accessible targets) given the absence of the fixed angle limitation of the guided technique (Figure S7). Readjustment and tract correction are more often needed with the free-hand technique, resulting in longer procedure duration, greater needle manipulation that could result in increased tissue trauma, and patient discomfort. New methods of image fusion and electromagnetic needle tracking may enable biopsy of targets that are difficult to visualize with ultrasound[^295^](#_ENREF_295)^,^[^296^](#_ENREF_296). The patient typically lies supine, although other positions may be required for specific sampling sites. After skin infiltration with local anesthetic, a pointed scalpel or thicker needle is used to create a small opening on the skin to facilitate insertion of the biopsy needle. Ideally, the target lesion should be no more than a few centimeters from the anterior abdominal wall without the interposition of other structures, such as small bowel loops. The procedure is not different from percutaneous biopsy of peripheral lymph nodes when a linear array probe >12 MHz is used. Once the biopsy is completed, a small plaster will be placed over the biopsy site.

- - 1. Processing of the sample

The preanalytical phase (specimen fixation) is critical for proper assessment of the tissue. The adequacy of the sample is influenced by the pre-analytical processing, and suboptimal handling may alter morphological, immunohistochemical and cytological characteristics of the tissue. Cold ischemia, fixation method, and time of paraffin block storage are factors that potentially affect the quality of the sample[^288^](#_ENREF_288). The specific requirements for sample preparation may vary and good communication between the pathologist and the operator is important to ensure sample adequacy[^35^](#_ENREF_35). Neutral buffered formalin is the commonly accepted fixative method and penetrates the tissue at 1 mm/h. It should only be used after 24 hours of dilution to 4% w/v (weight/volume), to guarantee a stable concentration[^288^](#_ENREF_288)^,^[^297^](#_ENREF_297)^,^[^298^](#_ENREF_298). The duration of the fixation is equally important as it influences the analysis of nucleic acids, proteins, and morphology. It may also influence the result of immunohistochemistry[^299^](#_ENREF_299). Optimal fixation is essential for immunohistochemical and molecular analysis. Minimal fixation of six hours and a maximum of 72 hours is recommended. The volume ratio of fixative to specimen size is very important for proper preservation of the tissue, i.e., a minimum of at least twice the volume of fixative as tissue is required. Small biopsies should be placed in at least 20mL of formalin. However, different methods for sample preservation may be used for some specific indications. For example, ideal samples for molecular testing are fresh tissues followed by snap freezing[^119^](#_ENREF_119). Piskorz *et al.* showed that for next-generation sequencing of DNA from high-grade serous ovarian cancer, methanol-based fixation has significant benefits over neutral buffered formalin in terms of DNA yield, length of fragment size and accuracy of copy-number calling[^263^](#_ENREF_263). Alcohol-based fixative is also commonly used as cytological fixative, as it better preserves nucleic acids[^298^](#_ENREF_298). In patients with suspected lymphoma, together with samples fixed in formalin, native material can also be submitted for flow cytometry, but it can vary, and it is not mandatory[^300^](#_ENREF_300). For suspected lymphoma, a rapid on-site evaluation (ROSE) for selection of adequate material to other analyses (flow cytometry) can be of help.

In general, all samples should be placed immediately after collection in a fixative solution (usually neutral buffered formalin) and sent to the pathology laboratory. Other fixative solutions other than neutral buffered formalin can be used if there is such a request following discussion in the multidisciplinary team. For most routine cases, an analytic turnaround time of 48 hours or less from receipt of the biopsy in the pathology laboratory is required. The use of immunohistochemistry is associated with an increase in processing time to 72 hours (an additional 24 hours is required for each immunohistochemical stain)[^301-303^](#_ENREF_301).

- - 1. Care of the procedure

Following uncomplicated procedures, there is no need for prolonged monitoring, and most patients are discharged after the procedure[^7^](#_ENREF_7)^,^[^12^](#_ENREF_12). In patients who are at a high risk of bleeding or other complications, a longer period (e.g. 4 to 6 hours for intraabdominal interventions) of close observation with regular blood pressure checks is recommended with the option of repeating ultrasound examination to look for the signs of intra-abdominal bleeding if indicated[^21^](#_ENREF_21)^,^[^289^](#_ENREF_289)^,^[^304^](#_ENREF_304)^,^[^305^](#_ENREF_305). Procedure-related complications usually manifest within 24 hours, and the patient should be informed about the symptoms of potential complications and provided with a written information sheet. Emergency contact details and information on how the results of the biopsy will be communicated with the patient should be included. Limiting intense physical activity for 24 to 48 hours may be advised, although the evidence for this recommendation is weak. No particular pharmacological treatment is universally recommended, although paracetamol or ibuprofen may be taken for abdominal/pelvic discomfort[^306^](#_ENREF_306).

If there are clinical signs suggestive of a complication (pain, discomfort, hemodynamic instability) or a significant hemoglobin decline (>2g/dL), the first investigation is ultrasound examination, which may be supplemented by other imaging studies (e. g. CT, angiography). Pain without a clinically or sonographically apparent cause is managed with standard analgesics and a short period of admission for observation.

Complications that arise as a result of ultrasound-guided interventions should be documented.

**Statement 23**: Before the procedure, the indication should be verified and the biopsy deemed to be clinically relevant.

- Level of evidence: 3b
- Grade of statement: C
- Consensus: yes, 100% (*n* = 18); no, 0% (*n* = 0); abstain, 0% (*n* = 0)

**Statement 24**: An ultrasound examination should be performed to select the safest path to the target lesion and subsequent approach.

- Level of evidence: 4
- Grade of statement: C
- Consensus: yes, 100% (*n* = 18); no, 0% (*n* = 0); abstain, 0% (*n* = 0)

**Statement 25**: Core-needle biopsy in gynecology does not require specific preparation such as fasting, or use of laxatives or antiflatulent medication.

- Level of evidence: 4
- Grade of statement: C
- Consensus: yes, 89% (*n* = 16); no, 0% (*n* = 0); abstain, 11% (*n* = 2)

**Statement 26**: The preparation should include providing the patient with procedure-related information, obtaining informed consent and identifying relevant medical history.

- Level of evidence: 3b
- Grade of statement: C
- Consensus: yes, 100% (*n* = 18); no, 0% (*n* = 0); abstain, 0% (*n* = 0)

**Statement 27**: Bleeding risk assessment should be performed according to the procedure-related and patient-related risks of bleeding.

- Level of evidence: 4
- Grade of statement: C
- Consensus: yes, 100% (*n* = 18); no, 0% (*n* = 0); abstain, 0% (*n* = 0)

**Statement 28**: Antibiotic prophylaxis is not recommended routinely, as the risk of infectious complications is low (< 1%). However, it should be considered on an individual basis, such as for the transrectal approach, if the needle passes through the rectal wall into the peritoneal cavity.

- Level of evidence: 4
- Grade of statement: C
- Consensus: yes, 94% (*n* = 17); no, 0% (*n* = 0); abstain, 6% (*n* = 1)

**Statement 29**: Maximum attention should be paid to minimize patient discomfort, pain and anxiety throughout the procedure.

- Level of evidence: 2b
- Grade of statement: B
- Consensus: yes, 100% (*n* = 18); no, 0% (*n* = 0); abstain, 0% (*n* = 0)

**Statement 30**: Basic disinfection procedures are sufficient. It is important to perform hand antisepsis. Clean handling of sterile instruments (needles) is recommended. All instruments should be laid out on a sterile trolley. The disinfected ultrasound probe should be covered with a sterile, disposable protective cover.

- Level of evidence: 4
- Grade of statement: C
- Consensus: yes, 89% (*n* = 16); no, 0% (*n* = 0); abstain, 11% (*n* = 2)

**Statement 31**: The biopsy device, type of single-use disposable needles, needle gauge, needle length and penetration depth should be based on the planned biopsy route.

- Level of evidence: 3b
- Grade of statement: B
- Consensus: yes, 94% (*n* = 17); no, 0% (*n* = 0); abstain, 6% (*n* = 1)

**Statement 32**: The puncture site should be cleansed with an antiseptic solution for transvaginal and percutaneous approaches.

- Level of evidence: 4
- Grade of statement: C
- Consensus: yes, 94% (*n* = 17); no, 0% (*n* = 0); abstain, 6% (*n* = 1)

**Statement 33**: Local anesthetic reduces discomfort caused by larger needle size and should be administered for all percutaneous biopsies. It can be administered optionally for transvaginal, transcervical and transrectal approaches.

- Level of evidence: 2b
- Grade of statement: B
- Consensus: yes, 94% (*n* = 17); no, 0% (*n* = 0); abstain, 6% (*n* = 1)

**Statement 34**: While performing the biopsy, the tip of the needle must be visible under continuous ultrasound control. If not, the procedure should be abandoned.

- Level of evidence: 5
- Grade of statement: D
- Consensus: yes, 89% (*n* = 16); no, 0% (*n* = 0); abstain, 11% (*n* = 2)

**Statement 35**: Specimen fixation is critical for proper assessment of the tissue. Core-needle biopsy specimen should be placed immediately in a formalin fixative solution and sent to the pathology laboratory. Optimal fixation is essential for immunohistochemical and molecular analysis. A minimum fixation time of 6 h and a maximum of 72 h is recommended.

- Level of evidence: 3b
- Grade of statement: B
- Consensus: yes, 67% (*n* = 12); no, 0% (*n* = 0); abstain, 33% (*n* = 6)

**Statement 36**: Ultrasound should be used at the end of the procedure to detect any signs of bleeding. Mild internal bleeding usually resolves spontaneously.

- Level of evidence: 5
- Grade of statement: D
- Consensus: yes, 94% (*n* = 17); no, 0% (*n* = 0); abstain, 6% (*n* = 1)

**Statement 37**: Following uncomplicated procedures, there is no need for prolonged monitoring. The patient should be informed about the symptoms of potential complications and provided with a written information sheet.

- Level of evidence: 4
- Grade of statement: C
- Consensus: yes, 94% (*n* = 17); no, 0% (*n* = 0); abstain, 6% (*n* = 1)

## Reporting

- - 1. Biopsy documentation

A detailed report regarding the procedure must be given to the patient and to her healthcare provider. The following data should be included: indication(s) for biopsy, preprocedural ultrasound findings, procedure description including biopsy device used, collection guidance (ultrasound) and approach (transvaginal/transrectal/transcervical/percutaneous), biopsy site, number of samples, adequacy of the sample, difficulty of the procedure, patient tolerance, any complications and when the results are expected to be communicated. Biopsy results may be available between 48 h and 10 days after delivery of the sample to the laboratory, depending on the complexity of tests required. Copies of the report and images or videoclips recording the position of the needle within the lesion should be stored for future reference.

- - 1. Specimen and biopsy data sheet

The request form for pathological examination should contain the patient’s history and clinical and radiological information in detail, including clinical diagnosis and differential diagnosis. In some situations, the biopsy is taken from multiple sites, and all specimens must be sent separately, clearly labeled, and documented in the request form[^307^](#_ENREF_307). The patient details on the specimen container and request form must be correct and match. Data on the request form and specimen containers are checked in the pathology laboratory before handling the specimen and when reporting. A detailed pathological report will then be discussed by the multidisciplinary team, taking into consideration all relevant patient data (Table S6)[^308-310^](#_ENREF_308).

**Table S6.** Pathology report

| **Items to be included on pathology report of fine-needle aspiration samples** |
| --- |
| **CLINICAL DATA**  Previous pathological diagnosis and treatments, relevant for diagnostic process  **SAMPLE**  Type of specimen (smear; fixed material in container)  Type of fixation (96% alcohol, 10% buffered formalin, other)  Processing of specimen (smear, cytospin slides, cytoblock)  Location of lesion(s) sampled, laterality    **CYTOLOGY REPORTING**  Evaluation of the sample (adequacy, preservation, amount of cellularity)  Limitations (specify)  Diagnosis:   - Benign, borderline, malignant, equivocal finding - Histological type of tumor, if present (according to latest WHO classification(s) of tumors) - Tumor grading (if applicable)   Optional:   - Microscopic description - Comment: (differential diagnosis, further recommendations)   If performed:   - Immunocytochemical results - Molecular testing results |
| **Items to be included on pathology report of core-needle biopsy samples** |
| **CLINICAL DATA**  Previous pathological diagnosis and treatments, relevant for diagnostic process  **SAMPLE**  Specimen (number of cores, core length/width)  Location of the lesion(s) sampled, laterality    **BIOPSY REPORTING**  Evaluation of the sample adequacy  Limitations (specify)  Diagnosis:   - Benign, borderline, malignant, equivocal finding - Histological type of tumor, if present (according to the latest WHO classification(s) of tumors) - Tumor grading (if applicable)   Optional:   - Microscopic description - Comment: differential diagnosis, further recommendations   If performed:   - Immunohistochemical results - Molecular testing results |

**Statement 38**: The request form for pathological examinations should contain:

- Patient identifiers, including age, gender and unique ID of the patient, which can differ among countries.
- All relevant clinical, radiological and patient history data.
- Details of the requesting doctor and contact details in case of emergency.
- The biopsy site(s), clinical diagnosis and differential diagnosis.
- Previous histopathological findings (if any).
- The type of specimen and type of fixative used.
- The patient details on the request form and specimen container must be correct and match.
- Level of evidence: 4
- Grade of statement: C
- Consensus: yes, 89% (*n* = 16); no, 0% (*n* = 0); abstain, 11% (*n* = 2)

**Statement 39**: The pathology report form should contain:

- Identification of the patient.
- Type of specimen and sample description.
- Type of processing (for fine-needle aspiration).
- Evaluation of the sample adequacy.
- Limitations.
- Diagnosis.
- Optional: microscopic description, immunohistochemical / immunocytochemical findings, molecular testing findings, differential diagnosis, recommendation.
- Level of evidence: 3a
- Grade of statement: B
- Consensus: yes, 94% (*n* = 17); no, 0% (*n* = 0); abstain, 6% (*n* = 1)

**Statement 40**: Analytic turnaround time of 2 days (business days counted only) after receipt of the sample at the pathology laboratory is required. If ancillary techniques such as immunohistochemistry are needed, the turnaround time is longer.

- Level of evidence: 3b
- Grade of statement: B
- Consensus: yes, 72% (*n*= 13); no, 0% (*n* = 0); abstain, 28% (*n* = 5)

## Training and quality assurance

To maintain high-quality and safety of image-guided biopsies, competent operators who are skilled in invasive diagnostic techniques and anatomy are required. This is crucial when accessing deep lesions in the female pelvis, which can be challenging due to the proximity of the major vessels, urinary bladder, ureters and bowel. The operator should possess a high-level understanding of both the theoretical and practical aspects of the imaging modality used for guidance and the interventional procedures. Developing the necessary skills and techniques involves a steep learning curve, and sufficient volume is required to maintain operator confidence[^97^](#_ENREF_97). Similarly, competent pathologists and cytopathologists are essential for accurate reading of the biopsy samples.

- - 1. For gynaecologists/radiologists

Learning interventional ultrasound should always be built upon the knowledge of diagnostic (non-interventional) ultrasound imaging of the area of interest[^311^](#_ENREF_311). It is advisable that core-needle biopsy is performed by examiners who already have an intermediate (level II) or advanced (level III) level of competence in gynecological ultrasound imaging or interventional/abdominal radiology (for the CT-/MRI- guided biopsy procedures)[^252^](#_ENREF_252). The European Federation of Societies for Ultrasound in Medicine and Biology has published minimum training requirements for gynecological ultrasound practice in Europe, including standards for theoretical knowledge and practical skills[^252^](#_ENREF_252). These identify three levels of ultrasound training and expertise: Level I, basic level discriminating normal findings from common pathological findings; Level II accept referrals from level I practitioners, recognize the vast majority of abnormalities, perform non-complex invasive procedures; Level 3 (expert) practitioners are likely to spend the majority of their time undertaking gynecological ultrasound and/or teaching, participating in ultrasound/related research and development, accepting tertiary referrals from Level I and II practitioners, and performing advanced ultrasound-guided invasive procedure. A Level-II practitioner should have performed at least 2000 gynecological ultrasound examinations. The training required to attain this level of practice would usually be gained during a period of expert ultrasound training, which may be within, or after completion of, a specialist training program. To maintain competence at Level II, practitioners should perform at least 500 gynecological ultrasound examinations per year[^252^](#_ENREF_252).

Training for invasive procedures should begin on a model/simulator (such as phantom based on a material simulating the organ of interest (e.g., gelatin, agar, paraffin wax gel and others) containing a target (e.g., olives), to practice maintenance of the needle path within the ultrasonic window, so that the entire needle always remains visible[^312-315^](#_ENREF_312). Studies indicate that the learning curve can be shortened by adding simulator-based or phantom-based training to clinical practice[^315^](#_ENREF_315). Using the same equipment as in clinical practice helps the operator to quickly become familiar with the equipment and to allow the training of the sensory-motor skills required for the integration of imaging and intervention. Computer simulations may also allow training without the need for phantoms of biopsy instruments. Simulations may help to maintain the required skills if the procedure is not regularly performed. Web-based teaching resources are also available[^316^](#_ENREF_316). Clinical training should begin under supervision by an experienced operator with ‘simple’ core-needle biopsy or fine-needle aspiration procedures (for example easily accessible diffuse thick carcinomatosis in pouch of Douglas using transvaginal approach through the posterior fornix with needle guide). Using the percutaneous approach to biopsy deep intra-abdominal lesions is among the most challenging to perform due to the distance from the skin to the target and difficulties in real-time needle tip visualization with the scanning plane. When performing percutaneous biopsy, commercially available sonographic guides attached to the transabdominal probe may provide more confidence regarding the needle position inside the body and are recommended for less experienced operators[^56^](#_ENREF_56)^,^[^102^](#_ENREF_102)^,^[^103^](#_ENREF_103)^,^[^294^](#_ENREF_294).

There is a lack of large studies assessing the impact of examiner experience and training on complication rate when performing pelvic core-needle biopsy. Studies on transvaginal and transabdominal chorionic villus sampling and amniocentesis show a reduced risk of fetal loss in high volume centers and when performed by more experienced examiners[^311^](#_ENREF_311)^,^[^317^](#_ENREF_317). A study by Ljung *et al.* found that in 1043 consecutive fine-needle aspiration specimens of the breast, samples obtained by a formally trained physician (at least 150 procedures performed) were significantly more cellular and were significantly more likely diagnostic[^318^](#_ENREF_318).

The minimum number of procedures required for operators to gain the requisite competence in gynecologic ultrasound-guided biopsies has not been well-defined. Studies within other areas have proposed a minimum training (from 50 to 150 biopsies) or a basic curriculum complete with textbooks, atlases, and online resources[^21^](#_ENREF_21)^,^[^319-321^](#_ENREF_319). Most data suggest no further improvement after 100 procedures performed independently. Available data emphasize the importance of carrying out a high volume of core-needle biopsy procedures, supervised by an experienced operator, before embarking on the procedure unsupervised. We recommend carrying out at least 20 core-needle biopsy procedures, supervised by an experienced operator, before embarking on the procedure unsupervised.

After achieving competence, operators are recommended to continue to perform these procedures on a regular basis. The Royal College of Obstetrics and Gynaecologists Green Top Guideline for amniocentesis and chorionic villus sampling suggests that each examiner performs at least 20 procedures annually to maintain competence[^320^](#_ENREF_320). It is therefore reasonable to adopt the same recommended minimal number, i.e., 20 core-needle biopsies annually/per operator.

Currently, there are formal postgraduate courses on core-needle biopsy in gynecologic oncology provided (e.g., IWUGO - the International Workshop on Ultrasound in Gynecologic Oncology). To reinforce theoretical knowledge in practice, individualized training may be available on request and by prior arrangement in centers specialized in gynecologic oncology scanning. Such a network of centers offering fellowship in gynecologic oncology scanning including core-needle biopsy training is available on the ESGO website.

There should be regular audits of sampling accuracy for malignant tissue and the rate of inadequate specimens submitted within each practice providing biopsy services[^319^](#_ENREF_319)^,^[^320^](#_ENREF_320). Similarly, reviews of complications and patient experience should be conducted, to identify and address the need for improvement and additional training of staff. The most important processes of care are (i) patient selection, (ii) performing the procedure, and (iii) monitoring the patient. The outcome measures (i.e., indicators) for these processes are indications, success rates (diagnostic yield), and complication rates. These outcome measures should be assigned threshold levels. When measures such as indications or success rates fall below a minimum threshold or when complication rates exceed a maximum threshold, a review should be performed to determine causes and to implement changes, if necessary. Regarding patient experience, there are studies aimed to prospectively assess the impact of biopsies on the quality of life in patients with gynecologic cancer, evaluate patient-reported outcomes, and determine factors associated with patients’ willingness to undergo sequential biopsies. The patient experience should be evaluated by assessing the procedure-related pain (scale 0-10), discomfort, loss of dignity, embarrassment, and willingness for future biopsies. Based on these results, preprocedural provision of patient information, peri-procedural psychosocial support, provision of a comfortable environment, and postprocedural monitoring of patient-reported complications may improve patients’ experience. Setting universal thresholds is very difficult, and each department is urged to adjust the thresholds as needed to higher or lower values to meet its own quality improvement program needs.

**Statement 41**: Training for operators:

- Training to at least intermediate (level II) or advanced (level III) level in gynecological ultrasound imaging is essential before commencing training in interventional ultrasound.
- Training in ultrasound-guided biopsy using phantoms and/or computer simulation improves skills and is useful prior to clinical training.
- Targeted training using directly supervised procedures is essential to reduce the risk of complications and increase sample adequacy.
- The needle-guiding system should be used by trainees for all approaches.
- At least 20 directly supervised core-needle biopsies using a needle guide should be performed before starting unsupervised work.
- Level of evidence: 4
- Grade of statement: B
- Consensus: yes, 89% (*n* = 16); no, 0% (*n* = 0); abstain, 11% (*n* = 2)

**Statement 42**: Maintaining competence for operators:

- Maintain competency by completing or supervising a minimum of 20 core-needle biopsy procedures annually.
- Seek support from a more experienced operator when difficulties are anticipated or encountered.
- Level of evidence: 3b
- Grade of statement: C
- Consensus: yes, 83% (*n* = 15); no, 0% (*n* = 0); abstain, 17% (*n* = 3)

**Statement 43**: Audit for operators:

- Regular audits should be undertaken within each practice providing biopsy services to ensure sampling adequacy and diagnostic yield, and to record complications and patient experience.
- Level of evidence: 3b
- Grade of statement: C
- Consensus: yes, 89% (*n* = 16); no, 0% (*n* = 0); abstain, 11% (*n* = 2)
  - 1. For pathologists

There is no universal agreement on which professional should be responsible for the analysis of the sample as it may be assigned to a general surgical pathologist or to a cytopathologist. This also depends on the adopted technique, and whether the procedure is a fine-needle aspiration or core-needle biopsy[^322^](#_ENREF_322). Minimum training requirements are currently included in curricula for fellows and educational programs, to achieve sufficient knowledge to work without supervision. For example, the ACGME (Accreditation Council for Graduate Medical Education) program requirements for graduate medical education in cytopathology include at least 500 specimens of fine-needle aspiration as a requisite for specialized practice[^323^](#_ENREF_323). The European Union of Medical Specialists (UEMS) Section & Board of Pathology recommends a total of 7500 surgical pathology cases, 2500 cytopathology cases and 50 complete molecular pathology reports as the minimal number in postgraduate training[^324^](#_ENREF_324). These criteria are defined by national authorities and can differ among countries.

Training competencies that pathologists should demonstrate include: (1) the ability to produce clear, concise, comprehensive and timely written reports for surgical pathology and cytopathology; (2) the ability to incorporate the diagnostic, prognostic or predictive implications of molecular pathology tests into an integrated pathology report; (3) promotion of health informatics to improve the quality of patient care and optimize patient safety; (4) participation in quality control, quality assurance and quality improvement initiatives; (5) utilization of genetic testing resources effectively to balance costs with potential utility of result; (6) alerting the treating physician when inheritable conditions are identified (e.g., genetic diseases that may affect the patient’s family members).

To maintain competence in pathological reporting, pathologists or cytopathologists should be a part of a gynecological oncology multidisciplinary team in a high-volume center.

As part of quality assurance, participation in accredited programs for all aspects of tissue diagnostics, for both clinical and non-clinical laboratories and organizations, is recommended[^322^](#_ENREF_322)^,^[^325^](#_ENREF_325)^,^[^326^](#_ENREF_326).

**Statement 44**: Training for pathologists/cytopathologists:

- Biopsies should be read by a pathologist or cytopathologist who has completed his/her postgraduate training in pathology/cytopathology. The rules are defined by national authorities and can differ among countries.
- Level of evidence: 5
- Grade of statement: C
- Consensus: yes, 89% (*n* = 16); no, 0% (*n* = 0); abstain, 11% (*n* = 2)

**Statement 45**: Maintaining competence for pathologists/cytopathologists:

- Ultrasound-guided biopsies should be performed in a specialized center with access to a pathologist or cytopathologist with experience in gynecological oncology as part of a multidisciplinary team.
- Level of evidence: 5
- Grade of statement: C
- Consensus: yes, 94% (*n* = 17); no, 0% (*n* = 0); abstain, 6% (*n* = 1)

**Statement 46**: Audit for pathologists/cytopathologists:

- Accreditation of laboratories should be in accordance with national or international standards (such as ISO15189). The rules of accreditation are defined by national authorities in each country and can differ.
- Level of evidence: 5
- Grade of statement: C
- Consensus: yes, 89% (*n* = 16); no, 0% (*n* = 0); abstain, 11% (*n* = 2)

1. **CONCLUSION**

Core-needle biopsy under ultrasound guidance is an emerging, minimally invasive outpatient procedure. It allows collection of high-quality specimens for histopathological diagnosis, immunohistochemical analysis, and molecular testing, enabling the timely commencement of appropriate treatment.

Performing core-needle biopsy and interpreting its results requires appropriate expertise and should be conducted within a multidisciplinary team. Under these circumstances, it is simple, quick, effective and safe. To ensure patient-centered care, standard operational procedures, including measures to minimize patient anxiety, pain and risk, are essential. This Consensus Statement aims to facilitate the implementation of this technique in gynecological oncology practice and improve patient outcomes.

**CITATION**

This Consensus Statement should be cited as: ‘Fischerova D, Planchamp F, Alcázar JL, Dundr P, Epstein E, Felix A, Frühauf F, Garganese G, Salvesen Haldorsen I, Jurkovic D, Kocian R, Lengyel D, Mascilini F, Stepanyan A, Stukan M, Timmerman S, Vanassche T, Yuan Ng Z, Scovazzi U. ISUOG/ESGO Consensus Statement on ultrasound-guided biopsy in gynecological oncology. *Ultrasound Obstet Gynecol* 2025. DOI: 10.1002.uog. 29183.’

**ACKNOWLEDGMENTS**

This article has been simultaneously co-published in Ultrasound in Obstetrics & Gynecology and International Journal of Gynecological Cancer. The articles are identical except for minor stylistic and spelling differences in keeping with each journal’s style. Any citation can be used when citing this article.

We thank Lucia Zanchi (MD, University of Pavia, Pavia, Italy), Natacha Sousa (MD, Hospital de Braga, Braga, Portugal) and Kim Hulscher (ENGAGE Co-Chair, European Network of Gynaecological Cancer Advocacy Groups) for intellectual contribution and suggestions during proofreading, Adam Preisler (Polygoniq Studio, Prague, Czech Republic) for providing the illustrations and Tomas Herrmann (Institute of Scientific Information, First Faculty of Medicine, Charles University Prague, Prague, Czech Republic) for videoclip editing. We thank ISUOG and ESGO for their support, especially Kamila Macku and Lenka Trestrova, who provided invaluable logistical and administrative support throughout the process. We wish also to express sincere gratitude to Maciej Malecki (University Hospital Leuven, Leuven, Belgium) for providing technical support during the voting process. All costs relating to the development process were covered by ISUOG and ESGO. There was no external funding for the development process or manuscript production.

**DISCLOSURE**

P.D. has reported being a member of advisory boards for AstraZeneca, Merck, MSD, GlaxoSmithKline, Amgen, Janssen-Cilag and Roche, and grants for travelling from AstraZeneca; A.F. has participated as a member of the speakers’ bureau for GlaxoSmithKline; T.V. has reported being a member of advisory boards for Bayer, Boehringer Ingelheim, BMS/Pfizer, Daiichi Sankyo, Sanofi Aventis, and Leo Pharma; D.F., F.P., J.L.A., E.E., F.F., G.G., I.S.H., D.J., R.K., D.L., F.M., A.S., M.S., S.T., Z.Y.N. and U.S. report no conflicts of interest.

**REFERENCES**

1. Cancer Genome Atlas Research, N. Integrated genomic analyses of ovarian carcinoma. *Nature* **474**, 609-615 (2011).

2. Cancer Genome Atlas Research Network, Albert Einstein College of Medicine Analytical Biological Services, et al. Integrated genomic and molecular characterization of cervical cancer. *Nature* **543**, 378-384 (2017).

3. Cancer Genome Atlas Research, N.*, et al.* Integrated genomic characterization of endometrial carcinoma. *Nature* **497**, 67-73 (2013).

4. Madariaga, A.*, et al.* Research biopsies in patients with gynecologic cancers: patient-reported outcomes, perceptions, and preferences. *Am J Obstet Gynecol* **225**, 658 e651-658 e659 (2021).

5. Gomez-Roca, C.A.*, et al.* Sequential research-related biopsies in phase I trials: acceptance, feasibility and safety. *Ann Oncol* **23**, 1301-1306 (2012).

6. Chi, D.S.*, et al.* Ten-year experience with laparoscopy on a gynecologic oncology service: analysis of risk factors for complications and conversion to laparotomy. *Am J Obstet Gynecol* **191**, 1138-1145 (2004).

7. Zikan, M., Fischerova, D., Pinkavova, I., Dundr, P. & Cibula, D. Ultrasound-guided tru-cut biopsy of abdominal and pelvic tumors in gynecology. *Ultrasound Obstet Gynecol* **36**, 767-772 (2010).

8. Harrison, R.F.*, et al.* Cost-effectiveness of laparoscopic disease assessment in patients with newly diagnosed advanced ovarian cancer. *Gynecol Oncol* **161**, 56-62 (2021).

9. van de Vrie, R.*, et al.* Cost-effectiveness of laparoscopy as diagnostic tool before primary cytoreductive surgery in ovarian cancer. *Gynecol Oncol* **146**, 449-456 (2017).

10. Lee, M.*, et al.* Comparisons of surgical outcomes, complications, and costs between laparotomy and laparoscopy in early-stage ovarian cancer. *Int J Gynecol Cancer* **21**, 251-256 (2011).

11. Goranova, T.*, et al.* Safety and utility of image-guided research biopsies in relapsed high-grade serous ovarian carcinoma-experience of the BriTROC consortium. *Br J Cancer* **116**, 1294-1301 (2017).

12. Fischerova, D.*, et al.* Ultrasound-guided tru-cut biopsy in the management of advanced abdomino-pelvic tumors. *Int J Gynecol Cancer* **18**, 833-837 (2008).

13. Epstein, E., Van Calster, B., Timmerman, D. & Nikman, S. Subjective ultrasound assessment, the ADNEX model and ultrasound-guided tru-cut biopsy to differentiate disseminated primary ovarian cancer from metastatic non-ovarian cancer. *Ultrasound Obstet Gynecol* **47**, 110-116 (2016).

14. Overman, M.J.*, et al.* Use of research biopsies in clinical trials: are risks and benefits adequately discussed? *J Clin Oncol* **31**, 17-22 (2013).

15. Penna, R., Poder, L., Jha, P., Seigel, E.L. & Morgan, T.A. Transvaginal Ultrasound-Guided Fine-Needle Aspiration Biopsy of Pelvic Lesions. *J Ultrasound Med* **41**, 653-661 (2022).

16. Birgin, E.*, et al.* Core needle biopsy versus incisional biopsy for differentiation of soft-tissue sarcomas: A systematic review and meta-analysis. *Cancer* **126**, 1917-1928 (2020).

17. Morris, S., Gurusamy, K.S., Sheringham, J. & Davidson, B.R. Cost-effectiveness of diagnostic laparoscopy for assessing resectability in pancreatic and periampullary cancer. *BMC Gastroenterol* **15**, 44 (2015).

18. Marks, J.M., Youngelman, D.F. & Berk, T. Cost analysis of diagnostic laparoscopy vs laparotomy in the evaluation of penetrating abdominal trauma. *Surg Endosc* **11**, 272-276 (1997).

19. Sheth, R.A.*, et al.* Society of Interventional Radiology Quality Improvement Standards on Percutaneous Needle Biopsy in Adult and Pediatric Patients. *J Vasc Interv Radiol* **31**, 1840-1848 (2020).

20. Gupta, S.*, et al.* Quality improvement guidelines for percutaneous needle biopsy. *J Vasc Interv Radiol* **21**, 969-975 (2010).

21. Neuberger, J.*, et al.* Guidelines on the use of liver biopsy in clinical practice from the British Society of Gastroenterology, the Royal College of Radiologists and the Royal College of Pathology. *Gut* **69**, 1382-1403 (2020).

22. Veltri, A., Bargellini, I., Giorgi, L., Almeida, P. & Akhan, O. CIRSE Guidelines on Percutaneous Needle Biopsy (PNB). *Cardiovasc Intervent Radiol* **40**, 1501-1513 (2017).

23. Sidhu, P.S.*, et al.* EFSUMB Guidelines on Interventional Ultrasound (INVUS), Part II. Diagnostic Ultrasound-Guided Interventional Procedures (Short Version). *Ultraschall Med* **36**, 566-580 (2015).

24. Wood, E.J., Pickhardt, P.J., Elissa, M., Mankowski Gettle, L. & Lubner, M.G. Ultrasound-guided transvaginal biopsies of pelvic lesions: diagnostic yield, safety profile, and technical considerations over a 20-year experience. *Abdom Radiol (NY)* **48**, 1154-1163 (2023).

25. Lengyel, D., Vereczkey, I., Kohalmy, K., Bahrehmand, K. & Novak, Z. Transvaginal Ultrasound-Guided Core Biopsy-Experiences in a Comprehensive Cancer Centre. *Cancers (Basel)* **13**(2021).

26. Tacher, V.*, et al.* Factors associated with success of image-guided tumour biopsies: Results from a prospective molecular triage study (MOSCATO-01). *Eur J Cancer* **59**, 79-89 (2016).

27. Von Hoff, D.D.*, et al.* Pilot study using molecular profiling of patients' tumors to find potential targets and select treatments for their refractory cancers. *J Clin Oncol* **28**, 4877-4883 (2010).

28. Lee, J.M.*, et al.* Feasibility and safety of sequential research-related tumor core biopsies in clinical trials. *Cancer* **119**, 1357-1364 (2013).

29. Basik, M.*, et al.* Biopsies: next-generation biospecimens for tailoring therapy. *Nat Rev Clin Oncol* **10**, 437-450 (2013).

30. O'Shea, A., Tam, A.L., Kilcoyne, A., Flaherty, K.T. & Lee, S.I. Image-guided biopsy in the age of personalised medicine: strategies for success and safety. *Clin Radiol* **76**, 154 e151-154 e159 (2021).

31. Bertucci, F.*, et al.* Prospective high-throughput genome profiling of advanced cancers: results of the PERMED-01 clinical trial. *Genome Med* **13**, 87 (2021).

32. Gambardella, V.*, et al.* Molecular profiling of advanced solid tumours. The impact of experimental molecular-matched therapies on cancer patient outcomes in early-phase trials: the MAST study. *Br J Cancer* **125**, 1261-1269 (2021).

33. Koyama, T.*, et al.* Practical consideration for successful sequential tumor biopsies in first-in-human trials. *Invest New Drugs* **40**, 841-849 (2022).

34. Le Tourneau, C.*, et al.* Molecularly targeted therapy based on tumour molecular profiling versus conventional therapy for advanced cancer (SHIVA): a multicentre, open-label, proof-of-concept, randomised, controlled phase 2 trial. *Lancet Oncol* **16**, 1324-1334 (2015).

35. Ferry-Galow, K.V.*, et al.* What Can Be Done to Improve Research Biopsy Quality in Oncology Clinical Trials? *J Oncol Pract* **14**, JOP1800092 (2018).

36. Morbi, A.H.*, et al.* Reducing error and improving efficiency during vascular interventional radiology: implementation of a preprocedural team rehearsal. *Radiology* **264**, 473-483 (2012).

37. Warshavsky, A.*, et al.* Core needle biopsy for diagnosing lymphoma in cervical lymphadenopathy: Meta-analysis. *Head Neck* **42**, 3051-3060 (2020).

38. Ettinger, D.S.*, et al.* NCCN Guidelines(R) Insights: Non-Small Cell Lung Cancer, Version 2.2023. *J Natl Compr Canc Netw* **21**, 340-350 (2023).

39. Sheafor, D.H., Paulson, E.K., Simmons, C.M., DeLong, D.M. & Nelson, R.C. Abdominal percutaneous interventional procedures: comparison of CT and US guidance. *Radiology* **207**, 705-710 (1998).

40. Hewitt, M.J.*, et al.* Women with peritoneal carcinomatosis of unknown origin: Efficacy of image-guided biopsy to determine site-specific diagnosis. *BJOG* **114**, 46-50 (2007).

41. Arnedos, M.*, et al.* Discordance between core needle biopsy (CNB) and excisional biopsy (EB) for estrogen receptor (ER), progesterone receptor (PgR) and HER2 status in early breast cancer (EBC). *Ann Oncol* **20**, 1948-1952 (2009).

42. Youk, J.H., Kim, E.K., Kim, M.J. & Oh, K.K. Sonographically guided 14-gauge core needle biopsy of breast masses: a review of 2,420 cases with long-term follow-up. *AJR Am J Roentgenol* **190**, 202-207 (2008).

43. Takenaka, A.*, et al.* A prospective randomized comparison of diagnostic efficacy between transperineal and transrectal 12-core prostate biopsy. *Prostate Cancer Prostatic Dis* **11**, 134-138 (2008).

44. Vu, T., Shin, B., Mittal, A., Sarwani, N. & McGillen, K.L. Ultrasound Versus Computed Tomography-Guided Native Parenchymal Kidney Biopsies for Hospitalized Patients: Comparison of Clinical Outcomes and Complications. *Ultrasound Q* **38**, 328-333 (2022).

45. Fischerova, D.*, et al.* Staging by imaging in gynecologic cancer and the role of ultrasound: an update of European joint consensus statements. *Int J Gynecol Cancer* **34**, 363-378 (2024).

46. Plett, S.K., Poder, L., Brooks, R.A. & Morgan, T.A. Transvaginal Ultrasound-Guided Biopsy of Deep Pelvic Masses: How We Do It. *J Ultrasound Med* **35**, 1113-1122 (2016).

47. Nakai, Y.*, et al.* Effect of Prolonged Duration of Transrectal Ultrasound-Guided Biopsy of the Prostate and Pre-Procedure Anxiety on Pain in Patients without Anesthesia. *Res Rep Urol* **13**, 111-120 (2021).

48. Won, S.Y., Kim, H.S. & Park, S.Y. Transrectal or transvaginal ultrasoundguided biopsy for pelvic masses: external validation and usefulness in oncologic patients. *Ultrasonography* **38**, 149-155 (2019).

49. Gao, C., Wang, L., Zhang, C. & Li, X. Transvaginal/transrectal ultrasound-guided aspiration biopsy for diagnosis of pelvic/pelvic floor tumors in females: A retrospective analysis. *Exp Ther Med* **18**, 352-357 (2019).

50. Rinnab, L., Kufer, R., Hautmann, R.E. & Gottfried, H.W. Use of transrectal ultrasound-guided biopsy in the diagnosis of pelvic malignancies. *J Clin Ultrasound* **34**, 440-445 (2006).

51. Stukan, M., Rutkowski, P., Smadja, J. & Bonvalot, S. Ultrasound-Guided Trans-Uterine Cavity Core Needle Biopsy of Uterine Myometrial Tumors to Differentiate Sarcoma from a Benign Lesion-Description of the Method and Review of the Literature. *Diagnostics (Basel)* **12**(2022).

52. Kawamura, N.*, et al.* Transcervical needle biopsy for the differential diagnosis between uterine sarcoma and leiomyoma. *Cancer* **94**, 1713-1720 (2002).

53. Kawamura, N.*, et al.* Transcervical needle biopsy of uterine myoma-like tumors using an automatic biopsy gun. *Fertil Steril* **77**, 1060-1064 (2002).

54. Gronchi, A.*, et al.* Soft tissue and visceral sarcomas: ESMO-EURACAN-GENTURIS Clinical Practice Guidelines for diagnosis, treatment and follow-up(☆). *Ann Oncol* **32**, 1348-1365 (2021).

55. Zheng, H.*, et al.* The accuracy of ultrasound-guided fine-needle aspiration and core needle biopsy in diagnosing axillary lymph nodes in women with breast cancer: a systematic review and meta-analysis. *Front Oncol* **13**, 1166035 (2023).

56. Shabana, W.*, et al.* Accuracy of sonographically guided biopsy using a freehand versus needle-guided technique: computed tomographic correlation study. *J Ultrasound Med* **32**, 535-540 (2013).

57. Elissa, M., Lubner, M.G. & Pickhardt, P.J. Biopsy of Deep Pelvic and Abdominal Targets With Ultrasound Guidance: Efficacy of Compression. *AJR Am J Roentgenol* **214**, 194-199 (2020).

58. Xie, J.*, et al.* MRI/Transrectal Ultrasound Fusion-Guided Targeted Biopsy and Transrectal Ultrasound-Guided Systematic Biopsy for Diagnosis of Prostate Cancer: A Systematic Review and Meta-analysis. *Front Oncol* **12**, 880336 (2022).

59. Garganese, G.*, et al.* Real-time ultrasound virtual navigation in 3D PET/CT volumes for superficial lymph-node evaluation: innovative fusion examination. *Ultrasound Obstet Gynecol* **58**, 766-772 (2021).

60. de Koekkoek-Doll, P.K.*, et al.* Real-Time Ultrasound Image Fusion with FDG-PET/CT to Perform Fused Image-Guided Fine-Needle Aspiration in Neck Nodes: Feasibility and Diagnostic Value. *AJNR Am J Neuroradiol* **42**, 566-572 (2021).

61. Wilson, S.R., Greenbaum, L.D. & Goldberg, B.B. Contrast-enhanced ultrasound: what is the evidence and what are the obstacles? *AJR Am J Roentgenol* **193**, 55-60 (2009).

62. Wu, W.*, et al.* The role of contrast-enhanced sonography of focal liver lesions before percutaneous biopsy. *AJR Am J Roentgenol* **187**, 752-761 (2006).

63. Yoon, S.H.*, et al.* Real-time contrast-enhanced ultrasound-guided biopsy of focal hepatic lesions not localised on B-mode ultrasound. *Eur Radiol* **20**, 2047-2056 (2010).

64. Aj, L.*, et al.* Fusion Image-Guided and Ultrasound-Guided Fine Needle Aspiration in Patients With Suspected Hepatic Metastases. *J Clin Exp Hepatol* **9**, 547-553 (2019).

65. Griffin, N.*, et al.* Image-guided biopsy in patients with suspected ovarian carcinoma: a safe and effective technique? *Eur Radiol* **19**, 230-235 (2009).

66. Carberry, G.A., Lubner, M.G., Wells, S.A. & Hinshaw, J.L. Percutaneous biopsy in the abdomen and pelvis: a step-by-step approach. *Abdom Radiol (NY)* **41**, 720-742 (2016).

67. Gupta, S.*, et al.* Various approaches for CT-guided percutaneous biopsy of deep pelvic lesions: anatomic and technical considerations. *Radiographics* **24**, 175-189 (2004).

68. Hoffmann, P.*, et al.* Long-term experience with percutaneous biopsies of pelvic lesions using CT guidance. *Sci Prog* **104**, 368504211058555 (2021).

69. Schiavon, L.H.O.*, et al.* Computed tomography-guided percutaneous biopsy of abdominal lesions: indications, techniques, results, and complications. *Radiol Bras* **51**, 141-146 (2018).

70. Chojniak, R.*, et al.* Computed tomography guided needle biopsy: experience from 1,300 procedures. *Sao Paulo Med J* **124**, 10-14 (2006).

71. Sugawara, S.*, et al.* Analysis of factors affecting the diagnostic yield of image-guided percutaneous core needle biopsy for peritoneal/omental lesions. *Abdom Radiol (NY)* **46**, 4499-4508 (2021).

72. Overduin, C.G., Futterer, J.J. & Barentsz, J.O. MRI-guided biopsy for prostate cancer detection: a systematic review of current clinical results. *Curr Urol Rep* **14**, 209-213 (2013).

73. El-Haddad, G. PET-Based Percutaneous Needle Biopsy. *PET Clin* **11**, 333-349 (2016).

74. Galgano, S.J.*, et al.* Applications of PET/MRI in Abdominopelvic Oncology. *Radiographics* **41**, 1750-1765 (2021).

75. Yarram, S.G.*, et al.* Evaluation of imaging-guided core biopsy of pelvic masses. *AJR Am J Roentgenol* **188**, 1208-1211 (2007).

76. Dadayal, G.*, et al.* Transvaginal ultrasound (TVUS)-guided biopsy is safe and effective in diagnosing peritoneal carcinomatosis and recurrent pelvic malignancy. *Clin Radiol* **71**, 1184-1192 (2016).

77. Giede, C., Toi, A., Chapman, W. & Rosen, B. The use of transrectal ultrasound to biopsy pelvic masses in women. *Gynecol Oncol* **95**, 552-556 (2004).

78. Atwell, T.D.*, et al.* Incidence of bleeding after 15,181 percutaneous biopsies and the role of aspirin. *AJR Am J Roentgenol* **194**, 784-789 (2010).

79. Pinto, P.*, et al.* OP12.05: Patient satisfaction with ultrasound, CT and WB-DWI/MRI for preoperative ovarian cancer staging: a multicentre prospective survey. . *Ultrasound Obstet Gynecol* **62**, 78-79 (2023).

80. Malmström, H. Fine-needle aspiration cytology versus core biopsies in the evaluation of recurrent gynecologic malignancies. *Gynecol Oncol* **65**, 69-73 (1997).

81. Pritzker, K.P.H. & Nieminen, H.J. Needle Biopsy Adequacy in the Era of Precision Medicine and Value-Based Health Care. *Arch Pathol Lab Med* **143**, 1399-1415 (2019).

82. Simundic, A.M. Measures of Diagnostic Accuracy: Basic Definitions. *EJIFCC* **19**, 203-211 (2009).

83. Park, H.Y., Suh, C.H. & Kim, S.O. Use of "Diagnostic Yield" in Imaging Research Reports: Results from Articles Published in Two General Radiology Journals. *Korean J Radiol* **23**, 1290-1300 (2022).

84. Strnad, B.S.*, et al.* Percutaneous Core Biopsy Devices: A Detailed Review and Comparison of Different Needle Designs. *Ultrasound Q* **40**, 1-19 (2024).

85. Poll, J.S. The story of the gauge. *Anaesthesia* **54**, 575-581 (1999).

86. ISO 6009:2016 Hypodermic needles for single use - Colour coding for identification. [*https://www.iso.org/obp/ui/en/#iso:std:iso:6009:ed-4:v1:en*](https://www.iso.org/obp/ui/en/#iso:std:iso:6009:ed-4:v1:en) (2016).

87. Cibula, D.*, et al.* ESGO/ESTRO/ESP Guidelines for the management of patients with cervical cancer - Update 2023. *Int J Gynecol Cancer* **33**, 649-666 (2023).

88. Gonzalez-Martin, A.*, et al.* Newly diagnosed and relapsed epithelial ovarian cancer: ESMO Clinical Practice Guideline for diagnosis, treatment and follow-up. *Ann Oncol* **34**, 833-848 (2023).

89. Nout, R.A.*, et al.* ESTRO/ESGO/SIOPe Guidelines for the management of patients with vaginal cancer. *Int J Gynecol Cancer* **33**, 1185-1202 (2023).

90. Oonk, M.H.M.*, et al.* European Society of Gynaecological Oncology Guidelines for the Management of Patients with Vulvar Cancer - Update 2023. *Int J Gynecol Cancer* **33**, 1023-1043 (2023).

91. Fischerova, D.*, et al.* The Role of Imaging in Cervical Cancer Staging: ESGO/ESTRO/ESP Guidelines (Update 2023). *Cancers (Basel)* **16**(2024).

92. NCCN Clinical Practice Guidelines in Oncology (NCCN Guidelines®). Ovarian Cancer Including Fallopian Tube Cancer and Primary Peritoneal Cancer. <https://www.nccn.org/professionals/physician_gls/pdf/ovarian.pdf> [Accessed 05/04/2024.].

93. Cheson, B.D.*, et al.* Recommendations for initial evaluation, staging, and response assessment of Hodgkin and non-Hodgkin lymphoma: the Lugano classification. *J Clin Oncol* **32**, 3059-3068 (2014).

94. German Guideline Program in Oncology (German Cancer Society, German Cancer Aid, AWMF): Soft Tissue Sarcoma Long version 1.1, 2022, AWMF Registration Number: 032/044OL Accessed [01/04/2024]. <https://www.leitlinienprogramm-onkologie.de/leitlinien/adulte-weichgewebesarkome/>.].

95. Lin, S.Y.*, et al.* Transvaginal Ultrasound-Guided Core Needle Biopsy of Pelvic Masses. *J Ultrasound Med* **37**, 453-461 (2018).

96. Mascilini, F.*, et al.* Role of transvaginal ultrasound-guided biopsy in gynecology. *Int J Gynecol Cancer* **30**, 128-132 (2020).

97. Verschuere, H.*, et al.* Safety and efficiency of performing transvaginal ultrasound-guided tru-cut biopsy for pelvic masses. *Gynecol Oncol* **161**, 845-851 (2021).

98. Park, J.J., Kim, C.K. & Park, B.K. Ultrasound-Guided Transvaginal Core Biopsy of Pelvic Masses: Feasibility, Safety, and Short-Term Follow-Up. *AJR Am J Roentgenol* **206**, 877-882 (2016).

99. Buonomo, F.*, et al.* Ultrasound-Guided Tru-Cut Biopsy in Gynecological and Non-Gynecological Pelvic Masses: A Single-Center Experience. *J Clin Med* **11**(2022).

100. Vlasak, P.*, et al.* Diagnostic Reliability, Accuracy and Safety of Ultrasound-guided Biopsy and Ascites Puncture in Primarily Inoperable Ovarian Tumours. *Anticancer Res* **40**, 3527-3534 (2020).

101. Mascilini, F.*, et al.* Transvaginal ultrasound-guided biopsy in patients with suspicious primary advanced tubo-ovarian carcinoma. *Int J Gynecol Cancer* **33**, 236-242 (2023).

102. Asp, M.*, et al.* Tru-Cut Biopsy in Gynecological Cancer: Adequacy, Accuracy, Safety and Clinical Applicability. *J Multidiscip Healthc* **16**, 1367-1377 (2023).

103. Leung, V.A., Kirpalani, A., Mnatzakanian, G., Colak, E. & Vlachou, P.A. Effect of a Biopsy Center on Adequacy Rates of Thyroid Nodule Fine-Needle Aspiration. *AJR Am J Roentgenol* **209**, 358-362 (2017).

104. Bhamidipati, D.*, et al.* An analysis of research biopsy core variability from over 5000 prospectively collected core samples. *NPJ Precis Oncol* **5**, 94 (2021).

105. Parsons, L.N., Vo, N., Moe, D.C. & Jarzembowski, J.A. Adequacy and Accuracy of Core Biopsy in Children: A Radiologic/Pathologic Correlation Study. *Pediatr Dev Pathol* **22**, 137-141 (2019).

106. Hoang, N.S.*, et al.* Determining the Optimal Number of Core Needle Biopsy Passes for Molecular Diagnostics. *Cardiovasc Intervent Radiol* **41**, 489-495 (2018).

107. Antunes, P.R.B.*, et al.* Clinical complications in renal biopsy using two different needle gauges: The impact of large hematomas, a random clinical trial study. *Int J Urol* **25**, 544-548 (2018).

108. Chunduri, S., Whittier, W.L. & Korbet, S.M. Adequacy and complication rates with 14- vs. 16-gauge automated needles in percutaneous renal biopsy of native kidneys. *Semin Dial* **28**, E11-14 (2015).

109. Bruinsma, R.S.*, et al.* Diagnostic accuracy of image-guided core needle biopsy of non-central nervous system tumors in children. *Pediatr Blood Cancer* **68**, e29179 (2021).

110. Obek, C., Doganca, T., Erdal, S., Erdogan, S. & Durak, H. Core length in prostate biopsy: size matters. *J Urol* **187**, 2051-2055 (2012).

111. Schmidt, R.L., Witt, B.L., Lopez-Calderon, L.E. & Layfield, L.J. The influence of rapid onsite evaluation on the adequacy rate of fine-needle aspiration cytology: a systematic review and meta-analysis. *Am J Clin Pathol* **139**, 300-308 (2013).

112. Kubik, M.J.*, et al.* Diagnostic value and accuracy of imprint cytology evaluation during image-guided core needle biopsies: Review of our experience at a large academic center. *Diagn Cytopathol* **43**, 773-779 (2015).

113. Li, Z., Tonkovich, D. & Shen, R. Impact of touch imprint cytology on imaging-guided core needle biopsies: An experience from a large academic medical center laboratory. *Diagn Cytopathol* **44**, 87-90 (2016).

114. Franchi, D.*, et al.* OC06.06: Ultrasound-guided trucut biopsy in gynecological disease: 15 years' experience in a tertiary referral cancer centre. *Ultrasound Obstet Gynecol* **56**, 17-17 (2020).

115. Batlle, C.*, et al.* Image-guided core biopsy for pelvic lesions of unknown origin. *Br J Surg* **111**(2024).

116. Perez, A.A., Lubner, M.G. & Pickhardt, P.J. Ultrasound-Guided Omental Biopsy: Diagnostic Yield and Association With CT Features Based on a Single-Institution 18-Year Series. *AJR Am J Roentgenol* **217**, 898-906 (2021).

117. Hill, D.K.*, et al.* Percutaneous omental biopsy: efficacy and complications. *Abdom Radiol (NY)* **42**, 1566-1570 (2017).

118. Wang, J.*, et al.* A retrospective analysis on the diagnostic value of ultrasound-guided percutaneous biopsy for peritoneal lesions. *World J Surg Oncol* **11**, 251 (2013).

119. Tam, A.L.*, et al.* Image-Guided Biopsy in the Era of Personalized Cancer Care: Proceedings from the Society of Interventional Radiology Research Consensus Panel. *J Vasc Interv Radiol* **27**, 8-19 (2016).

120. Austin, M.C., Smith, C., Pritchard, C.C. & Tait, J.F. DNA Yield From Tissue Samples in Surgical Pathology and Minimum Tissue Requirements for Molecular Testing. *Arch Pathol Lab Med* **140**, 130-133 (2016).

121. Denkert, C.*, et al.* Homologous Recombination Deficiency as an Ovarian Cancer Biomarker in a Real-World Cohort: Validation of Decentralized Genomic Profiling. *J Mol Diagn* **24**, 1254-1263 (2022).

122. Mirza, M.R.*, et al.* The forefront of ovarian cancer therapy: update on PARP inhibitors. *Ann Oncol* **31**, 1148-1159 (2020).

123. Callens, C.*, et al.* Concordance Between Tumor and Germline BRCA Status in High-Grade Ovarian Carcinoma Patients in the Phase III PAOLA-1/ENGOT-ov25 Trial. *J Natl Cancer Inst* **113**, 917-923 (2021).

124. Cho, M.*, et al.* Tissue recommendations for precision cancer therapy using next generation sequencing: a comprehensive single cancer center's experiences. *Oncotarget* **8**, 42478-42486 (2017).

125. Magliacane, G.*, et al.* Locally Performed HRD Testing for Ovarian Cancer? Yes, We Can! *Cancers (Basel)* **15**(2022).

126. Goswami, R.S.*, et al.* Identification of Factors Affecting the Success of Next-Generation Sequencing Testing in Solid Tumors. *Am J Clin Pathol* **145**, 222-237 (2016).

127. Kubo, T.*, et al.* A meta-analysis supports core needle biopsy by radiologists for better histological diagnosis in soft tissue and bone sarcomas. *Medicine (Baltimore)* **97**, e11567 (2018).

128. Almond, L.M.*, et al.* Diagnostic accuracy of percutaneous biopsy in retroperitoneal sarcoma. *Br J Surg* **106**, 395-403 (2019).

129. Luca Alatzides, G.*, et al.* Is preoperative CT-guided biopsy a valuable tool in the diagnostic workup of patients with visceral and retroperitoneal sarcoma? *Eur J Radiol* **155**, 110470 (2022).

130. Ciccarone, F.*, et al.* A clinical ultrasound algorithm to identify uterine sarcoma and smooth muscle tumors of uncertain malignant potential in patients with myometrial lesions: the MYometrial Lesion UltrasouNd And mRi study. *Am J Obstet Gynecol* **232**, 108.e101-108.e122 (2025).

131. Smadja, J.*, et al.* Percutaneous Uterine Needle Biopsy with Microscopic and Array-CGH Analyses for Preoperative Sarcoma Diagnosis in Patients with Suspicious Myometrial Tumors on MRI: A Prospective Pilot Study (SARCGYN). *Ann Surg Oncol* **30**, 943-953 (2023).

132. Weigl, H.*, et al.* Accuracy and Safety of Ultrasound-Guided Core Needle Biopsy of Soft Tissue Tumors in an Outpatient Setting: A Sarcoma Center Analysis of 392 Consecutive Patients. *Cancers (Basel)* **13**(2021).

133. Wu, J.S., Goldsmith, J.D., Horwich, P.J., Shetty, S.K. & Hochman, M.G. Bone and soft-tissue lesions: what factors affect diagnostic yield of image-guided core-needle biopsy? *Radiology* **248**, 962-970 (2008).

134. Ray-Coquard, I.*, et al.* ESGO/EURACAN/GCIG guidelines for the management of patients with uterine sarcomas. *Int J Gynecol Cancer* **34**, 1499-1521 (2024).

135. Seviar, D.*, et al.* Image-guided core needle biopsy as the first-line diagnostic approach in lymphoproliferative disorders-A review of the current literature. *Eur J Haematol* **106**, 139-147 (2021).

136. Hu, Q.*, et al.* Needle-core biopsy in the pathologic diagnosis of malignant lymphoma showing high reproducibility among pathologists. *Am J Clin Pathol* **140**, 238-247 (2013).

137. Hehn, S.T., Grogan, T.M. & Miller, T.P. Utility of fine-needle aspiration as a diagnostic technique in lymphoma. *J Clin Oncol* **22**, 3046-3052 (2004).

138. Groneck, L., Quaas, A., Hallek, M., Zander, T. & Weihrauch, M.R. Ultrasound-guided core needle biopsies for workup of lymphadenopathy and lymphoma. *Eur J Haematol* **97**, 379-386 (2016).

139. Lachar, W.A., Shahab, I. & Saad, A.J. Accuracy and cost-effectiveness of core needle biopsy in the evaluation of suspected lymphoma: a study of 101 cases. *Arch Pathol Lab Med* **131**, 1033-1039 (2007).

140. Fitzpatrick, M.J., Sohani, A.R. & Ly, A. Uses and limitations of small-volume biopsies for the diagnosis of lymphoma. *Cytopathology* **35**, 454-463 (2024).

141. Dindo, D., Demartines, N. & Clavien, P.A. Classification of surgical complications: a new proposal with evaluation in a cohort of 6336 patients and results of a survey. *Ann Surg* **240**, 205-213 (2004).

142. Khalilzadeh, O.*, et al.* Proposal of a New Adverse Event Classification by the Society of Interventional Radiology Standards of Practice Committee. *J Vasc Interv Radiol* **28**, 1432-1437 e1433 (2017).

143. Liang, P.*, et al.* US-guided percutaneous needle biopsy of the spleen using 18-gauge versus 21-gauge needles. *J Clin Ultrasound* **35**, 477-482 (2007).

144. Swobodnik, W.*, et al.* [Comparison of ultrasound-controlled fine needle and coarse needle puncture of defined lesions in the abdomen]. *Ultraschall Med* **11**, 287-289 (1990).

145. Piccinino, F., Sagnelli, E., Pasquale, G. & Giusti, G. Complications following percutaneous liver biopsy. A multicentre retrospective study on 68,276 biopsies. *J Hepatol* **2**, 165-173 (1986).

146. Mueller, M.*, et al.* Percutaneous ultrasonographically guided liver punctures: an analysis of 1961 patients over a period of ten years. *BMC Gastroenterol* **12**, 173 (2012).

147. Chevallier, P.*, et al.* Influence of operator experience on performance of ultrasound-guided percutaneous liver biopsy. *Eur Radiol* **14**, 2086-2091 (2004).

148. Que, Y.*, et al.* Nodules in the thickened greater omentum: a good indicator of lesions? *J Ultrasound Med* **28**, 745-748 (2009).

149. Que, Y.*, et al.* Ultrasound-guided biopsy of greater omentum: an effective method to trace the origin of unclear ascites. *Eur J Radiol* **70**, 331-335 (2009).

150. Souza, F.F., Mortele, K.J., Cibas, E.S., Erturk, S.M. & Silverman, S.G. Predictive value of percutaneous imaging-guided biopsy of peritoneal and omental masses: results in 111 patients. *AJR Am J Roentgenol* **192**, 131-136 (2009).

151. Frieser, M.*, et al.* [Spectrum and bleeding complications of sonographically guided interventions of the liver and pancreas]. *Ultraschall Med* **30**, 168-174 (2009).

152. Padia, S.A.*, et al.* Safety and efficacy of sonographic-guided random real-time core needle biopsy of the liver. *J Clin Ultrasound* **37**, 138-143 (2009).

153. Riemann, B., Menzel, J., Schiemann, U., Domschke, W. & Konturek, J.W. Ultrasound-guided biopsies of abdominal organs with an automatic biopsy system. A retrospective analysis of the quality of biopsies and of hemorrhagic complications. *Scand J Gastroenterol* **35**, 102-107 (2000).

154. Kong, T.W.*, et al.* Transvaginal Sonography-Guided Core Biopsy of Adnexal Masses as a Useful Diagnostic Alternative Replacing Cytologic Examination or Laparoscopy in Advanced Ovarian Cancer Patients. *Int J Gynecol Cancer* **26**, 1041-1047 (2016).

155. Lia, M.*, et al.* The diagnostic value of core needle biopsy in cervical cancer: A retrospective analysis. *PLoS One* **17**, e0262257 (2022).

156. Oge, T.*, et al.* Sonographically guided core biopsy: a minimally invasive procedure for managing adnexal masses. *J Ultrasound Med* **32**, 2023-2027 (2013).

157. Pelayo-Delgado, I.*, et al.* Contribution of Outpatient Ultrasound Transvaginal Biopsy and Puncture in the Diagnosis and Treatment of Pelvic Lesions: A Bicenter Study. *Diagnostics (Basel)* **13**(2023).

158. Govindarajan, P. & Keshava, S.N. Ultrasound-guided omental biopsy: Review of 173 patients. *Indian J Radiol Imaging* **20**, 307-309 (2010).

159. Cadranel, J.F., Rufat, P. & Degos, F. Practices of liver biopsy in France: results of a prospective nationwide survey. For the Group of Epidemiology of the French Association for the Study of the Liver (AFEF). *Hepatology* **32**, 477-481 (2000).

160. Strobel, D.*, et al.* Incidence of bleeding in 8172 percutaneous ultrasound-guided intraabdominal diagnostic and therapeutic interventions - results of the prospective multicenter DEGUM interventional ultrasound study (PIUS study). *Ultraschall Med* **36**, 122-131 (2015).

161. Khajehdehi, P., Junaid, S.M., Salinas-Madrigal, L., Schmitz, P.G. & Bastani, B. Percutaneous renal biopsy in the 1990s: safety, value, and implications for early hospital discharge. *Am J Kidney Dis* **34**, 92-97 (1999).

162. Corapi, K.M., Chen, J.L., Balk, E.M. & Gordon, C.E. Bleeding complications of native kidney biopsy: a systematic review and meta-analysis. *Am J Kidney Dis* **60**, 62-73 (2012).

163. Farmer, K.D., Harries, S.R., Fox, B.M., Maskell, G.F. & Farrow, R. Core biopsy of the bowel wall: efficacy and safety in the clinical setting. *AJR Am J Roentgenol* **175**, 1627-1630 (2000).

164. Levy, M.J.*, et al.* Prospective study of bacteremia and complications With EUS FNA of rectal and perirectal lesions. *Clin Gastroenterol Hepatol* **5**, 684-689 (2007).

165. Loeb, S.*, et al.* Systematic review of complications of prostate biopsy. *Eur Urol* **64**, 876-892 (2013).

166. Pilatz, A.*, et al.* Antibiotic Prophylaxis for the Prevention of Infectious Complications following Prostate Biopsy: A Systematic Review and Meta-Analysis. *J Urol* **204**, 224-230 (2020).

167. Ross, J.T., Matthay, M.A. & Harris, H.W. Secondary peritonitis: principles of diagnosis and intervention. *BMJ* **361**, k1407 (2018).

168. de'Angelis, N.*, et al.* 2017 WSES guidelines for the management of iatrogenic colonoscopy perforation. *World J Emerg Surg* **13**, 5 (2018).

169. Tudor, G.R., Rodgers, P.M. & West, K.P. Bowel lesions: percutaneous US-guided 18-gauge needle biopsy--preliminary experience. *Radiology* **212**, 594-597 (1999).

170. Fisher, A.J., Paulson, E.K., Sheafor, D.H., Simmons, C.M. & Nelson, R.C. Small lymph nodes of the abdomen, pelvis, and retroperitoneum: usefulness of sonographically guided biopsy. *Radiology* **205**, 185-190 (1997).

171. Brandt, K.R., Charboneau, J.W., Stephens, D.H., Welch, T.J. & Goellner, J.R. CT- and US-guided biopsy of the pancreas. *Radiology* **187**, 99-104 (1993).

172. Vadvala, H.V.*, et al.* Image-Guided Percutaneous Omental and Mesenteric Biopsy: Assessment of Technical Success Rate and Diagnostic Yield. *J Vasc Interv Radiol* **28**, 1569-1576 (2017).

173. Petit, P., Bret, P.M., Lough, J.O. & Reinhold, C. Risks associated with intestinal perforation during experimental percutaneous drainage. *Invest Radiol* **27**, 1012-1019 (1992).

174. Cubberley, D.A. Is it appropriate to perform a diagnostic aspiration of an intraabdominal fluid collection through the stomach, duodenum, or small bowel? *AJR Am J Roentgenol* **165**, 1005 (1995).

175. Hsu, M.Y.*, et al.* CT-guided percutaneous core-needle biopsy of pancreatic masses: comparison of the standard mesenteric/retroperitoneal versus the trans-organ approaches. *Clin Radiol* **71**, 507-512 (2016).

176. Rodriguez, S., Haimovich, S., Vitale, S.G., Alonso, L. & Carugno, J. Vasovagal Syncope during Office Hysteroscopy-A Frequently Overlooked Unpleasant Complication. *Medicina (Kaunas)* **58**(2022).

177. Zanetta, G.*, et al.* Early and short-term complications after US-guided puncture of gynecologic lesions: evaluation after 1,000 consecutive cases. *Radiology* **189**, 161-164 (1993).

178. Thabet, A.*, et al.* Image-guided ovarian mass biopsy: efficacy and safety. *J Vasc Interv Radiol* **25**, 1922-1927 e1921 (2014).

179. Silva, M.A.*, et al.* Needle track seeding following biopsy of liver lesions in the diagnosis of hepatocellular cancer: a systematic review and meta-analysis. *Gut* **57**, 1592-1596 (2008).

180. Cresswell, A.B., Welsh, F.K. & Rees, M. A diagnostic paradigm for resectable liver lesions: to biopsy or not to biopsy? *HPB (Oxford)* **11**, 533-540 (2009).

181. Jones, O.M., Rees, M., John, T.G., Bygrave, S. & Plant, G. Biopsy of resectable colorectal liver metastases causes tumour dissemination and adversely affects survival after liver resection. *Br J Surg* **92**, 1165-1168 (2005).

182. Staehler, M.*, et al.* Long-Term Follow-Up in Patients Undergoing Renal Mass Biopsy: Seeding is not Anecdotal. *Clin Genitourin Cancer* **22**, 189-192 (2024).

183. Ljungberg, B.*, et al.* European Association of Urology Guidelines on Renal Cell Carcinoma: The 2022 Update. *Eur Urol* **82**, 399-410 (2022).

184. Van Houdt, W.J.*, et al.* Needle tract seeding following core biopsies in retroperitoneal sarcoma. *Eur J Surg Oncol* **43**, 1740-1745 (2017).

185. Berger-Richardson, D. & Swallow, C.J. Needle tract seeding after percutaneous biopsy of sarcoma: Risk/benefit considerations. *Cancer* **123**, 560-567 (2017).

186. Berger-Richardson, D.*, et al.* Early and Late Complications of Percutaneous Core Needle Biopsy of Retroperitoneal Tumors at Two Tertiary Sarcoma Centers. *Ann Surg Oncol* **26**, 4692-4698 (2019).

187. Wilkinson, M.J.*, et al.* Percutaneous core needle biopsy in retroperitoneal sarcomas does not influence local recurrence or overall survival. *Ann Surg Oncol* **22**, 853-858 (2015).

188. Eriksson, M.*, et al.* Needle biopsy through the abdominal wall for the diagnosis of gastrointestinal stromal tumour - Does it increase the risk for tumour cell seeding and recurrence? *Eur J Cancer* **59**, 128-133 (2016).

189. Martini, M.*, et al.* To Obtain More With Less: Cytologic Samples With Ancillary Molecular Techniques-The Useful Role of Liquid-Based Cytology. *Arch Pathol Lab Med* **142**, 299-307 (2018).

190. Mokhatri, M., Shekarkhar, G. & Sarraf, Z. Fine-Needle Aspiration Biopsies of Ovarian Masses: A Reliable Technique. *Acta Cytol* **60**, 465-474 (2016).

191. Ganjei, P., Dickinson, B., Harrison, T., Nassiri, M. & Lu, Y. Aspiration cytology of neoplastic and non-neoplastic ovarian cysts: is it accurate? *Int J Gynecol Pathol* **15**, 94-101 (1996).

192. Khan, N., Afroz, N., Aqil, B., Khan, T. & Ahmad, I. Neoplastic and nonneoplastic ovarian masses: Diagnosis on cytology. *J Cytol* **26**, 129-133 (2009).

193. Gupta, P.*, et al.* Ultrasound-guided fine needle aspiration of ovarian masses: Assessment of diagnostic accuracy and risk stratification using a categorical reporting system. *Cytopathology* **32**, 441-458 (2021).

194. Acanfora, G.*, et al.* A roadmap for a comprehensive diagnostic approach to fine needle cytology of lymph node metastases. *Cytopathology* **33**, 668-677 (2022).

195. Petrovic, N., Arko, D., Lovrec, V.G. & Takac, I. Ultrasound guided aspiration in pathological adnexal processes. *Eur J Obstet Gynecol Reprod Biol* **104**, 52-57 (2002).

196. Shao, H.*, et al.* CT-Guided Percutaneous Needle Biopsy of Retroperitoneal and Pelvic Lymphadenopathy: Assessment of Technique, Diagnostic Yield, and Clinical Value. *J Vasc Interv Radiol* **29**, 1429-1436 (2018).

197. Dupain, C.*, et al.* Fine-needle aspiration as an alternative to core needle biopsy for tumour molecular profiling in precision oncology: prospective comparative study of next-generation sequencing in cancer patients included in the SHIVA02 trial. *Mol Oncol* **15**, 104-115 (2021).

198. Kanagal-Shamanna, R.*, et al.* Next-generation sequencing-based multi-gene mutation profiling of solid tumors using fine needle aspiration samples: promises and challenges for routine clinical diagnostics. *Mod Pathol* **27**, 314-327 (2014).

199. Roy-Chowdhuri, S.*, et al.* Concurrent fine needle aspirations and core needle biopsies: a comparative study of substrates for next-generation sequencing in solid organ malignancies. *Mod Pathol* **30**, 499-508 (2017).

200. Fassan, M. Molecular Diagnostics in Pathology: Time for a Next-Generation Pathologist? *Arch Pathol Lab Med* **142**, 313-320 (2018).

201. Diaz de la Noval, B.*, et al.* Transvaginal Ultrasound-Guided Fine-Needle Aspiration of Adnexal Cysts With a Low Risk of Malignancy: Our Experience and Recommendations. *J Ultrasound Med* **39**, 1787-1797 (2020).

202. Zanetta, G.*, et al.* Role of puncture and aspiration in expectant management of simple ovarian cysts: a randomised study. *BMJ* **313**, 1110-1113 (1996).

203. Ameye, L.*, et al.* Clinically oriented three-step strategy for assessment of adnexal pathology. *Ultrasound Obstet Gynecol* **40**, 582-591 (2012).

204. Layfield, L.J., Heaps, J.M. & Berek, J.S. Fine-needle aspiration cytology accuracy with palpable gynecologic neoplasms. *Gynecol Oncol* **40**, 70-73 (1991).

205. Zanetta, G.*, et al.* Transvaginal ultrasound-guided fine needle sampling of deep cancer recurrences in the pelvis: usefulness and limitations. *Gynecol Oncol* **54**, 59-63 (1994).

206. Gupta, N.*, et al.* Fine needle aspiration cytology in ovarian lesions: an institutional experience of 584 cases. *Cytopathology* **23**, 300-307 (2012).

207. Eitan, R.*, et al.* Diagnosis of deep pelvic masses on a gynaecology service: Trans-vaginal ultrasound-guided needle aspiration of pelvic solid and cystic lesions. *Aust N Z J Obstet Gynaecol* **57**, 197-200 (2017).

208. Ha, H.J.*, et al.* Utility and Limitations of Fine-Needle Aspiration Cytology in the Diagnosis of Lymphadenopathy. *Diagnostics (Basel)* **13**(2023).

209. VanderLaan, P.A. Fine-needle aspiration and core needle biopsy: An update on 2 common minimally invasive tissue sampling modalities. *Cancer Cytopathol* **124**, 862-870 (2016).

210. Imachi, M.*, et al.* Fine-needle aspiration cytology in patients with gynecologic malignancies. *Gynecol Oncol* **46**, 309-312 (1992).

211. Stockberger, S.M., Jr.*, et al.* Abdominal and pelvic needle aspiration biopsies: can we perform them well when using small needles? *Abdom Imaging* **24**, 321-328 (1999).

212. Pisharodi, L.R. & Attal, H. Fine needle aspiration cytology of vaginal cuff lesions. *Acta Cytol* **44**, 147-150 (2000).

213. Ray, S., Gangopadhyay, M., Bandyopadhyay, A., Majumdar, K. & Chaudhury, N. USG guided FNAC of ovarian mass lesions: A cyto-histopathological correlation, with emphasis on its role in pre-operative management guidelines. *J Turk Ger Gynecol Assoc* **15**, 6-12 (2014).

214. Lin, D.M., Barkan, G.A., Chatt, G., Park, J.W. & Gattuso, P. Vaginal fine-needle aspiration: A useful alternative to biopsy. *Diagn Cytopathol* **44**, 665-669 (2016).

215. Kar, A., Satapathy, B., Pattnaik, K. & Dash, P.K. Trucut Biopsy vs FNAC of Pelvic Tumors-Who Wins the Match? *J Cytol* **35**, 179-182 (2018).

216. Nyman, R.S., Cappelen-Smith, J., Brismar, J., von Sinner, W. & Kagevi, I. Yield and complications in ultrasound-guided biopsy of abdominal lesions. Comparison of fine-needle aspiration biopsy and 1.2-mm needle core biopsy using an automated biopsy gun. *Acta Radiol* **36**, 485-490 (1995).

217. Female Genital Tumours - WHO Classification of Tumours, 5th Edition, Volume 4. [*https://publications.iarc.fr/Book-And-Report-Series/Who-Classification-Of-Tumours/Female-Genital-Tumours-2020*](https://publications.iarc.fr/Book-And-Report-Series/Who-Classification-Of-Tumours/Female-Genital-Tumours-2020) (2020).

218. Goyal, A. Role of Fine Needle Aspiration Cytology in the Diagnosis of Gynecologic Tumors. *Acta Cytol* **67**, 195-212 (2023).

219. Pinto, P.*, et al.* Prediction of Surgical Outcome in Advanced Ovarian Cancer by Imaging and Laparoscopy: A Narrative Review. *Cancers (Basel)* **15**(2023).

220. Fischerova, D.*, et al.* Yolk Sac Tumor of the Omentum: A Case Report and Literature Review. *Diagnostics (Basel)* **12**(2022).

221. Fischerova, D.*, et al.* Primary retroperitoneal nodal endometrioid carcinoma associated with Lynch syndrome: A case report. *Front Oncol* **13**, 1092044 (2023).

222. Ledermann, J.A.*, et al.* ESGO-ESMO-ESP consensus conference recommendations on ovarian cancer: pathology and molecular biology and early, advanced and recurrent disease. *Ann Oncol* **35**, 248-266 (2024).

223. Roy, D.*, et al.* Transrectal ultrasound-guided biopsy of recurrent cervical carcinoma. *Br J Radiol* **81**, 902-906 (2008).

224. Russo, A.*, et al.* The challenge of the Molecular Tumor Board empowerment in clinical oncology practice: A Position Paper on behalf of the AIOM- SIAPEC/IAP-SIBioC-SIC-SIF-SIGU-SIRM Italian Scientific Societies. *Crit Rev Oncol Hematol* **169**, 103567 (2022).

225. Stockley, T.L.*, et al.* Molecular profiling of advanced solid tumors and patient outcomes with genotype-matched clinical trials: the Princess Margaret IMPACT/COMPACT trial. *Genome Med* **8**, 109 (2016).

226. Khleif, S.N., Doroshow, J.H., Hait, W.N. & Collaborative, A.-F.-N.C.B. AACR-FDA-NCI Cancer Biomarkers Collaborative consensus report: advancing the use of biomarkers in cancer drug development. *Clin Cancer Res* **16**, 3299-3318 (2010).

227. Levit, L.A.*, et al.* Ethical Framework for Including Research Biopsies in Oncology Clinical Trials: American Society of Clinical Oncology Research Statement. *J Clin Oncol* **37**, 2368-2377 (2019).

228. Patel, I.J.*, et al.* Society of Interventional Radiology Consensus Guidelines for the Periprocedural Management of Thrombotic and Bleeding Risk in Patients Undergoing Percutaneous Image-Guided Interventions-Part II: Recommendations: Endorsed by the Canadian Association for Interventional Radiology and the Cardiovascular and Interventional Radiological Society of Europe. *J Vasc Interv Radiol* **30**, 1168-1184 e1161 (2019).

229. Atwell, T.D.*, et al.* Peri-procedural use of anticoagulants in radiology: an evidence-based review. *Abdom Radiol (NY)* **42**, 1556-1565 (2017).

230. Baron, T.H., Kamath, P.S. & McBane, R.D. Management of antithrombotic therapy in patients undergoing invasive procedures. *N Engl J Med* **368**, 2113-2124 (2013).

231. Davidson, J.C.*, et al.* Society of Interventional Radiology Consensus Guidelines for the Periprocedural Management of Thrombotic and Bleeding Risk in Patients Undergoing Percutaneous Image-Guided Interventions-Part I: Review of Anticoagulation Agents and Clinical Considerations: Endorsed by the Canadian Association for Interventional Radiology and the Cardiovascular and Interventional Radiological Society of Europe. *J Vasc Interv Radiol* **30**, 1155-1167 (2019).

232. Tafur, A.J.*, et al.* Predictors of major bleeding in peri-procedural anticoagulation management. *J Thromb Haemost* **10**, 261-267 (2012).

233. Patel, I.J.*, et al.* Consensus guidelines for periprocedural management of coagulation status and hemostasis risk in percutaneous image-guided interventions. *J Vasc Interv Radiol* **23**, 727-736 (2012).

234. Hall, D.P.*, et al.* Plasma transfusions prior to insertion of central lines for people with abnormal coagulation. *Cochrane Database Syst Rev* **9**, CD011756 (2016).

235. Estcourt, L.J., Desborough, M., Hopewell, S., Doree, C. & Stanworth, S.J. Comparison of different platelet transfusion thresholds prior to insertion of central lines in patients with thrombocytopenia. *Cochrane Database Syst Rev* **2015**, CD011771 (2015).

236. Schiffer, C.A.*, et al.* Platelet Transfusion for Patients With Cancer: American Society of Clinical Oncology Clinical Practice Guideline Update. *J Clin Oncol* **36**, 283-299 (2018).

237. Kaufman, R.M.*, et al.* Platelet transfusion: a clinical practice guideline from the AABB. *Ann Intern Med* **162**, 205-213 (2015).

238. Ault, M.J., Rosen, B.T., Scher, J., Feinglass, J. & Barsuk, J.H. Thoracentesis outcomes: a 12-year experience. *Thorax* **70**, 127-132 (2015).

239. Grabau, C.M.*, et al.* Performance standards for therapeutic abdominal paracentesis. *Hepatology* **40**, 484-488 (2004).

240. McVay, P.A. & Toy, P.T. Lack of increased bleeding after liver biopsy in patients with mild hemostatic abnormalities. *Am J Clin Pathol* **94**, 747-753 (1990).

241. Potretzke, T.A.*, et al.* Frequency of Bleeding Complications After Percutaneous Core Needle Biopsy and the Association With Aspirin Usage and Length of Aspirin Discontinuation. *AJR Am J Roentgenol* **213**, 211-215 (2019).

242. Hadi, M.*, et al.* CIRSE Standards of Practice on Peri-operative Anticoagulation Management During Interventional Radiology Procedures. *Cardiovasc Intervent Radiol* **44**, 523-536 (2021).

243. Douketis, J.D. Perioperative management of patients who are receiving warfarin therapy: an evidence-based and practical approach. *Blood* **117**, 5044-5049 (2011).

244. Somerville, P., Seifert, P.J., Destounis, S.V., Murphy, P.F. & Young, W. Anticoagulation and bleeding risk after core needle biopsy. *AJR Am J Roentgenol* **191**, 1194-1197 (2008).

245. Veitch, A.M.*, et al.* Endoscopy in patients on antiplatelet or anticoagulant therapy: British Society of Gastroenterology (BSG) and European Society of Gastrointestinal Endoscopy (ESGE) guideline update. *Gut* **70**, 1611-1628 (2021).

246. Carmignani, L.*, et al.* Transrectal ultrasound-guided prostate biopsies in patients taking aspirin for cardiovascular disease: A meta-analysis. *Transfus Apher Sci* **45**, 275-280 (2011).

247. Beyer-Westendorf, J.*, et al.* Peri-interventional management of novel oral anticoagulants in daily care: results from the prospective Dresden NOAC registry. *Eur Heart J* **35**, 1888-1896 (2014).

248. Hornor, M.A.*, et al.* American College of Surgeons' Guidelines for the Perioperative Management of Antithrombotic Medication. *J Am Coll Surg* **227**, 521-536 e521 (2018).

249. Doherty, J.U.*, et al.* 2017 ACC Expert Consensus Decision Pathway for Periprocedural Management of Anticoagulation in Patients With Nonvalvular Atrial Fibrillation: A Report of the American College of Cardiology Clinical Expert Consensus Document Task Force. *J Am Coll Cardiol* **69**, 871-898 (2017).

250. Bell, B.R., Spyropoulos, A.C. & Douketis, J.D. Perioperative Management of the Direct Oral Anticoagulants: A Case-Based Review. *Hematol Oncol Clin North Am* **30**, 1073-1084 (2016).

251. Douketis, J.D. & Gregory, Y.H.L. Perioperative management of patients receiving anticoagulants. in *UpToDate* (ed. Lawrence, L.L.) (UpToDate, Waltham, MA. (accessed on March 17, 2023), 2022).

252. Minimum training recommendations for the practice of medical ultrasound. *Ultraschall Med* **27**, 79-105 (2006).

253. Subtil, J.C.*, et al.* Gastrointestinal Endoscopic Ultrasound-Guided Fine-Needle Aspiration for Assessing Suspected Deep Pelvic or Abdominal Recurrence in Gynecologic Cancer: A Feasibility Study. *J Ultrasound Med* **38**, 761-765 (2019).

254. Vergote, I.*, et al.* Prognostic importance of degree of differentiation and cyst rupture in stage I invasive epithelial ovarian carcinoma. *Lancet* **357**, 176-182 (2001).

255. Matsuo, K.*, et al.* Minimally Invasive Surgery and Risk of Capsule Rupture for Women With Early-Stage Ovarian Cancer. *JAMA Oncol* **6**, 1110-1113 (2020).

256. Alieva, M., van Rheenen, J. & Broekman, M.L.D. Potential impact of invasive surgical procedures on primary tumor growth and metastasis. *Clin Exp Metastasis* **35**, 319-331 (2018).

257. Syrykh, C.*, et al.* Lymph node excisions provide more precise lymphoma diagnoses than core biopsies: a French Lymphopath network survey. *Blood* **140**, 2573-2583 (2022).

258. Demharter, J.*, et al.* Percutaneous core-needle biopsy of enlarged lymph nodes in the diagnosis and subclassification of malignant lymphomas. *Eur Radiol* **11**, 276-283 (2001).

259. Vandervelde, C.*, et al.* A study to evaluate the efficacy of image-guided core biopsy in the diagnosis and management of lymphoma--results in 103 biopsies. *Eur J Radiol* **66**, 107-111 (2008).

260. Lutjeboer, J., Burgmans, M.C., Chung, K. & van Erkel, A.R. Impact on Patient Safety and Satisfaction of Implementation of an Outpatient Clinic in Interventional Radiology (IPSIPOLI-Study): A Quasi-Experimental Prospective Study. *Cardiovasc Intervent Radiol* **38**, 543-551 (2015).

261. Venkatesan, A.M.*, et al.* Practice guidelines for adult antibiotic prophylaxis during vascular and interventional radiology procedures. Written by the Standards of Practice Committee for the Society of Interventional Radiology and Endorsed by the Cardiovascular Interventional Radiological Society of Europe and Canadian Interventional Radiology Association [corrected]. *J Vasc Interv Radiol* **21**, 1611-1630; quiz 1631 (2010).

262. Pereira, N., Hutchinson, A.P., Lekovich, J.P., Hobeika, E. & Elias, R.T. Antibiotic Prophylaxis for Gynecologic Procedures prior to and during the Utilization of Assisted Reproductive Technologies: A Systematic Review. *J Pathog* **2016**, 4698314 (2016).

263. Piskorz, A.M.*, et al.* Methanol-based fixation is superior to buffered formalin for next-generation sequencing of DNA from clinical cancer samples. *Ann Oncol* **27**, 532-539 (2016).

264. Olson, E.M., Lin, N.U., Krop, I.E. & Winer, E.P. The ethical use of mandatory research biopsies. *Nat Rev Clin Oncol* **8**, 620-625 (2011).

265. Siegal, D.*, et al.* Periprocedural heparin bridging in patients receiving vitamin K antagonists: systematic review and meta-analysis of bleeding and thromboembolic rates. *Circulation* **126**, 1630-1639 (2012).

266. Kuo, H.C.*, et al.* Thromboembolic and bleeding risk of periprocedural bridging anticoagulation: A systematic review and meta-analysis. *Clin Cardiol* **43**, 441-449 (2020).

267. Douketis, J.D.*, et al.* The Perioperative Anticoagulant Use for Surgery Evaluation (PAUSE) Study for Patients on a Direct Oral Anticoagulant Who Need an Elective Surgery or Procedure: Design and Rationale. *Thromb Haemost* **117**, 2415-2424 (2017).

268. Douketis, J.D.*, et al.* Perioperative Bridging Anticoagulation in Patients with Atrial Fibrillation. *N Engl J Med* **373**, 823-833 (2015).

269. Weinreb, E.B., Cholst, I.N., Ledger, W.J., Danis, R.B. & Rosenwaks, Z. Should all oocyte donors receive prophylactic antibiotics for retrieval? *Fertil Steril* **94**, 2935-2937 (2010).

270. Cervini, P., Hesley, G.K., Thompson, R.L., Sampathkumar, P. & Knudsen, J.M. Incidence of infectious complications after an ultrasound-guided intervention. *AJR Am J Roentgenol* **195**, 846-850 (2010).

271. Lightner, D.J., Wymer, K., Sanchez, J. & Kavoussi, L. Best Practice Statement on Urologic Procedures and Antimicrobial Prophylaxis. *J Urol* **203**, 351-356 (2020).

272. Chehab, M.A.*, et al.* Adult and Pediatric Antibiotic Prophylaxis during Vascular and IR Procedures: A Society of Interventional Radiology Practice Parameter Update Endorsed by the Cardiovascular and Interventional Radiological Society of Europe and the Canadian Association for Interventional Radiology. *J Vasc Interv Radiol* **29**, 1483-1501 e1482 (2018).

273. Committee, A.S.o.P.*, et al.* Antibiotic prophylaxis for GI endoscopy. *Gastrointest Endosc* **81**, 81-89 (2015).

274. Grimm, L.J.*, et al.* Now or Later? Patient Satisfaction and Anxiety Among Women Undergoing Breast Biopsies Performed the Same Day as Recommended Versus a Later Day. *J Am Coll Radiol* **21**, 415-424 (2024).

275. Barlas, T., Sodan, H.N., Avci, S., Cerit, E.T. & Yalcin, M.M. The impact of classical music on anxiety and pain perception during a thyroid fine needle aspiration biopsy. *Hormones (Athens)* **22**, 581-585 (2023).

276. Gurkan, O. & Kaya, M.F. Effect of Music on Anxiety and Pain Levels of Patients Undergoing Thyroid Fine Needle Aspiration Biopsy: A Randomized Controlled Study. *Acad Radiol* **31**, 538-543 (2024).

277. Buisman, E.*, et al.* Trends in research on pain relief during oocyte retrieval for IVF/ICSI: a systematic, methodological review. *Hum Reprod Open* **2022**, hoac006 (2022).

278. Greenberger, C., Matot, I., Artsi, H., Samara, N. & Azem, F. High level of satisfaction among women who underwent oocyte retrieval without anesthesia. *Fertil Steril* **114**, 354-360 (2020).

279. Kwan, I., Wang, R., Pearce, E. & Bhattacharya, S. Pain relief for women undergoing oocyte retrieval for assisted reproduction. *Cochrane Database Syst Rev* **5**, CD004829 (2018).

280. Lee, M.S.*, et al.* Guidelines for Transrectal Ultrasonography-Guided Prostate Biopsy: Korean Society of Urogenital Radiology Consensus Statement for Patient Preparation, Standard Technique, and Biopsy-Related Pain Management. *Korean J Radiol* **21**, 422-430 (2020).

281. Bettocchi, S.*, et al.* Challenging the cervix: strategies to overcome the anatomic impediments to hysteroscopy: analysis of 31,052 office hysteroscopies. *Fertil Steril* **105**, e16-e17 (2016).

282. Ahmad, G., Attarbashi, S., O'Flynn, H. & Watson, A.J. Pain relief in office gynaecology: a systematic review and meta-analysis. *Eur J Obstet Gynecol Reprod Biol* **155**, 3-13 (2011).

283. Salazar, C.A. & Isaacson, K.B. Office Operative Hysteroscopy: An Update. *J Minim Invasive Gynecol* **25**, 199-208 (2018).

284. Shah, A., Cohen, K., Patel, B., Dahiya, N. & Fananapazir, G. Optimizing anxiolysis and analgesia for percutaneous intervention by the abdominal radiologist. *Abdom Radiol (NY)* **47**, 2721-2729 (2022).

285. Hubner, N.O., Siebert, J. & Kramer, A. Octenidine dihydrochloride, a modern antiseptic for skin, mucous membranes and wounds. *Skin Pharmacol Physiol* **23**, 244-258 (2010).

286. Peel, T.N., Dowsey, M.M., Buising, K.L., Cheng, A.C. & Choong, P.F.M. Chlorhexidine-alcohol versus iodine-alcohol for surgical site skin preparation in an elective arthroplasty (ACAISA) study: a cluster randomized controlled trial. *Clin Microbiol Infect* **25**, 1239-1245 (2019).

287. Peel, T.N., Watson, E. & Lee, S.J. Randomised Controlled Trials of Alcohol-Based Surgical Site Skin Preparation for the Prevention of Surgical Site Infections: Systematic Review and Meta-Analysis. *J Clin Med* **10**(2021).

288. Souza da Silva, R., Pinto, R., Cirnes, L. & Schmitt, F. Tissue management in precision medicine: What the pathologist needs to know in the molecular era. *Front Mol Biosci* **9**, 983102 (2022).

289. Kim, K.W.*, et al.* Value of "patent track" sign on Doppler sonography after percutaneous liver biopsy in detection of postbiopsy bleeding: a prospective study in 352 patients. *AJR Am J Roentgenol* **189**, 109-116 (2007).

290. Scanlan, K.A., Propeck, P.A. & Lee, F.T., Jr. Invasive procedures in the female pelvis: value of transabdominal, endovaginal, and endorectal US guidance. *Radiographics* **21**, 491-506 (2001).

291. Carey, J.M. & Korman, H.J. Transrectal ultrasound guided biopsy of the prostate. Do enemas decrease clinically significant complications? *J Urol* **166**, 82-85 (2001).

292. Zani, E.L., Clark, O.A. & Rodrigues Netto, N., Jr. Antibiotic prophylaxis for transrectal prostate biopsy. *Cochrane Database Syst Rev*, CD006576 (2011).

293. Duraes, M., Mandoul, C., Perrochia, H. & Rathat, G. Fertility-sparing management of a suspicion of leiomyosarcoma: avoiding hysterectomy by performing transcervical myoma biopsy. *Fertil Steril* **117**, 230-231 (2022).

294. Bluvol, N.*, et al.* Freehand versus guided breast biopsy: comparison of accuracy, needle motion, and biopsy time in a tissue model. *AJR Am J Roentgenol* **192**, 1720-1725 (2009).

295. Buckner, C.A., Venkatesan, A., Locklin, J.K. & Wood, B.J. Real-time sonography with electromagnetic tracking navigation for biopsy of a hepatic neoplasm seen only on arterial phase computed tomography. *J Ultrasound Med* **30**, 253-256 (2011).

296. Dencker, D., Topsoe, J.F., Ewertsen, C. & Karstrup, S. Image Fusion and Electromagnetic Needle Tracking for the Biopsy of Pelvic Lesions - Report of 2 Cases. *Ultrasound Int Open* **1**, E30-32 (2015).

297. Thavarajah, R., Mudimbaimannar, V.K., Elizabeth, J., Rao, U.K. & Ranganathan, K. Chemical and physical basics of routine formaldehyde fixation. *J Oral Maxillofac Pathol* **16**, 400-405 (2012).

298. Cree, I.A.*, et al.* Guidance for laboratories performing molecular pathology for cancer patients. *J Clin Pathol* **67**, 923-931 (2014).

299. Bass, B.P., Engel, K.B., Greytak, S.R. & Moore, H.M. A review of preanalytical factors affecting molecular, protein, and morphological analysis of formalin-fixed, paraffin-embedded (FFPE) tissue: how well do you know your FFPE specimen? *Arch Pathol Lab Med* **138**, 1520-1530 (2014).

300. Ramsay, A., Pomplun, S. & Wilkins, B. Tissue pathways for lymph node, spleen and bone marrow trephine biopsy specimens. [*https://www.rcpath.org/static/a2780c20-edc8-4023-a7e5bd45175dce76/G062-Tissue-pathways-for-lymph-node-spleen-and-bone-marrow-trephine-biopsy-specimens-For-publication.pdf*](https://www.rcpath.org/static/a2780c20-edc8-4023-a7e5bd45175dce76/G062-Tissue-pathways-for-lymph-node-spleen-and-bone-marrow-trephine-biopsy-specimens-For-publication.pdf) (2017).

301. Alshieban, S. & Al-Surimi, K. Reducing turnaround time of surgical pathology reports in pathology and laboratory medicine departments. *BMJ Qual Improv Rep* **4**(2015).

302. Novis, D.A., Zarbo, R.J. & Saladino, A.J. Interinstitutional comparison of surgical biopsy diagnosis turnaround time: a College of American Pathologists Q-Probes study of 5384 surgical biopsies in 157 small hospitals. *Arch Pathol Lab Med* **122**, 951-956 (1998).

303. Zarbo, R.J., Gephardt, G.N. & Howanitz, P.J. Intralaboratory timeliness of surgical pathology reports. Results of two College of American Pathologists Q-Probes studies of biopsies and complex specimens. *Arch Pathol Lab Med* **120**, 234-244 (1996).

304. Lorentzen, T.*, et al.* EFSUMB Guidelines on Interventional Ultrasound (INVUS), Part I. General Aspects (long Version). *Ultraschall Med* **36**, E1-14 (2015).

305. Khati, N.J., Gorodenker, J. & Hill, M.C. Ultrasound-guided biopsies of the abdomen. *Ultrasound Q* **27**, 255-268 (2011).

306. Stone, R.*, et al.* Enhanced Recovery and Surgical Optimization Protocol for Minimally Invasive Gynecologic Surgery: An AAGL White Paper. *J Minim Invasive Gynecol* **28**, 179-203 (2021).

307. Lee, M.J., Fanelli, F., Haage, P., Hausegger, K. & Van Lienden, K.P. Patient safety in interventional radiology: a CIRSE IR checklist. *Cardiovasc Intervent Radiol* **35**, 244-246 (2012).

308. Renshaw, S.A., Mena-Allauca, M., Touriz, M., Renshaw, A. & Gould, E.W. The impact of template format on the completeness of surgical pathology reports. *Arch Pathol Lab Med* **138**, 121-124 (2014).

309. Sluijter, C.E., van Lonkhuijzen, L.R., van Slooten, H.J., Nagtegaal, I.D. & Overbeek, L.I. The effects of implementing synoptic pathology reporting in cancer diagnosis: a systematic review. *Virchows Arch* **468**, 639-649 (2016).

310. Schaad, N., Berezowska, S., Perren, A. & Hewer, E. Impact of template-based synoptic reporting on completeness of surgical pathology reports. *Virchows Arch* (2023).

311. Bakker, M.*, et al.* Total pregnancy loss after chorionic villus sampling and amniocentesis: a cohort study. *Ultrasound Obstet Gynecol* **49**, 599-606 (2017).

312. Armstrong, S.A., Jafary, R., Forsythe, J.S. & Gregory, S.D. Tissue-Mimicking Materials for Ultrasound-Guided Needle Intervention Phantoms: A Comprehensive Review. *Ultrasound Med Biol* **49**, 18-30 (2023).

313. Wang, Y.*, et al.* Fabrication of SEBS Block Copolymer-Based Ultrasound Phantom Containing Mimic Tumors for Ultrasound-Guided Needle Biopsy Training. *Ultrasound Med Biol* **48**, 1143-1150 (2022).

314. Zhao, X., Ersoy, E. & Ng, D.L. Comparison of low-cost phantoms for ultrasound-guided fine-needle aspiration biopsy training. *J Am Soc Cytopathol* **12**, 275-283 (2023).

315. Sekhar, A., Sun, M.R. & Siewert, B. A tissue phantom model for training residents in ultrasound-guided liver biopsy. *Acad Radiol* **21**, 902-908 (2014).

316. Ferreira, A. How to perform Sonosalpingography? Visual Encyclopedia of Ultrasound in Obstetric and Gynecology. [*https://www.isuog.org*](https://www.isuog.org) (2023).

317. Tabor, A., Vestergaard, C.H. & Lidegaard, O. Fetal loss rate after chorionic villus sampling and amniocentesis: an 11-year national registry study. *Ultrasound Obstet Gynecol* **34**, 19-24 (2009).

318. Ljung, B.M.*, et al.* Diagnostic accuracy of fine-needle aspiration biopsy is determined by physician training in sampling technique. *Cancer* **93**, 263-268 (2001).

319. Ghi, T.*, et al.* ISUOG Practice Guidelines: invasive procedures for prenatal diagnosis. *Ultrasound Obstet Gynecol* **48**, 256-268 (2016).

320. Navaratnam, K., Alfirevic, Z., Royal College of, O. & Gynaecologists. Amniocentesis and chorionic villus sampling: Green-top Guideline No. 8 July 2021: Green-top Guideline No. 8. *BJOG* **129**, e1-e15 (2022).

321. Jenssen, C.*, et al.* EFSUMB Guidelines on Interventional Ultrasound (INVUS), Part IV - EUS-guided Interventions: General aspects and EUS-guided sampling (Long Version). *Ultraschall Med* **37**, E33-76 (2016).

322. Akhtar, I., Khurana, K., Staats, P., Monaco, S.E. & Florence, R. To whom the specimen goes: a look at how touch preparations and core needle biopsies are handled in different practices and the effect on fellowship education. *J Am Soc Cytopathol* **10**, 510-516 (2021).

323. ACGME Program Requirements for Graduate Medical Education in Cytopathology. (Accreditation Council for Graduate Medical Education, 2020).

324. UEMS. UEMS 2019.44 European Training Requirements in Pathology. (2019).

325. <https://www.cap.org/laboratory-improvement/proficiency-testing/new-surveys-and-anatomic-pathology-education-programs>.

326. <https://www.ukneqascpt.org>.

**Supplementary materials to Appendix S1**

**Videoclip S1** Core-needle biopsy (transvaginal ultrasound approach): live demonstration of the preprocedural preparation (including selection of the biopsy site and approach), hygiene and instrumentarium setup, procedure execution and postoperative management of women undergoing transvaginal core-needle biopsy.

**Videoclip S2** Core-needle biopsy (transrectal ultrasound approach): live demonstration of the preprocedural preparation, hygiene and instrumentarium setup, procedure execution and postoperative management of women undergoing transrectal core-needle biopsy (cases in which the transvaginal approach is not feasible).

**Videoclip S3** Core-needle biopsy (percutaneous ultrasound approach): live demonstration of the preprocedural preparation, hygiene and instrumentarium setup, procedure execution (including management of analgesia) and postoperative management of women undergoing percutaneous core-needle biopsy

**Videoclip S4** Overview of the indications for core-needle biopsy encountered in the gynecological oncology unit with presentation of clinical cases.

**Appendix S2.** Sample patient leaflet

*ISUOG/ESGO Consensus Statement on ultrasound-guided biopsy in gynecologic oncology - Patient leaflet*

**Introduction**

Ultrasound-guided biopsy is a type of minimally invasive (small puncture) procedure that allows your doctor to obtain a small amount of tissue or fluid which can then be analyzed in the laboratory. In order to obtain a sufficient sample for analysis, the biopsy is navigated by ultrasound imaging.

Two procedures are used in gynecological practice:

- Core-needle biopsy
- Fine-needle aspiration

Depending on the reason for the biopsy and the type of tests required, your doctor will advise which is the most suitable procedure for you.

**Purpose of the procedure**

There are many reasons for performing an ultrasound-guided biopsy, which include:

- Initial diagnosis of cancer
- Diagnosis of cancer recurrence
- Predicting the response of cancer to certain medications
- Clarifying whether a growth or lump is cancerous and its organ of origin
- Research (development of modern drugs for precision medicine)

**What to expect**

Ultrasound-guided biopsy is a safe, relatively painless and outpatient procedure. Your doctor will advise you if any specific blood tests are needed prior to the procedure, and will check whether you are currently on any medications and if you have any allergies. Prior to the biopsy, typically no fasting or specific preparation is required. Your doctor will inform you the expected turnaround time for the results to become available, which will depend on the types of tests that are required.

On the day of the biopsy, no fasting is required. Minor discomfort, mild bleeding and pain at the biopsy site are possible and usually resolve spontaneously. Painkillers may be used to alleviate any pain or discomfort. In certain situations, sedation may be used to ensure no major pain or discomfort is experienced. More severe complications are rare and include severe bleeding from the biopsy site, infections, and fever. In such an event, it is important to seek medical advice and return to the performing center or the A&E (accident and emergency place at the hospital). In rare situations, admission to the hospital with/without surgery may be required to diagnose and treat any complications from the procedure.

Ultrasound-guided biopsy may be performed via the percutaneous (through the skin to various structures in the abdomen, groin, axila, etc.), transvaginal or transrectal (back passage) routes. Your doctor will select the safest and most accurate method of performing the biopsy. The entire procedure typically takes approximately 5-10 minutes.

At the end of the procedure, you will be able to go home. Sometimes, you may be asked to stay for a slightly longer period (up to 2-3 hours) for observation. Your doctor will inform you regarding any subsequent appointments for discussion of the results of the biopsy. There are no specific limitations for daily activity after the biopsy, however avoiding strenuous physical activity and heavy exercises is advisable for the initial 2-3 days.

**Appendix S3.** Identification of scientific evidence

| Literature search in MEDLINE | |
| --- | --- |
|  |  |
|  |  |
| Research period | 01/1951 – 12/2023 |
|  |  |
|  |  |
| Indexing terms | "biopsy, large core-needle"[MeSH Terms] OR "biopsy, fine-needle"[MeSH Terms] OR "Tru-cut biopsy"[Title/Abstract]) AND ("genital neoplasms, female"[MeSH Terms] OR "Gynecology"[MeSH Terms] OR "Pelvic neoplasms"[MeSH Terms] OR "gynecologic* oncology"[Title/Abstract] OR "gynecologic* cancer*"[Title/Abstract] OR "pelvic tumor*"[Title/Abstract] OR "Carcinomatosis"[Title/Abstract] OR "ovarian cancer*"[Title/Abstract] OR "mass*"[Title/Abstract] |
|  |  |
|  |  |
| Language | English |
|  |  |
|  |  |
| Study design | Priority was given to high-quality systematic reviews, meta-analyses, and randomized controlled trials but lower levels of evidence were also evaluated. The search strategy excluded editorials, letters, case reports and in vitro studies. |
|  |  |
|  |  |

**Appendix S4** Levels of evidence and grades of statement used in this Consensus Statement

| *Levels of evidence* | | |
| --- | --- | --- |
|  |  | |
| 1a | Systematic review (with homogeneity) of Level-1 diagnostic studies; or clinical decision rule with Level-1b studies from different clinical centers | |
|  |  | |
|  |  | |
| 1b | Validating cohort study with good reference standards; or clinical decision rule tested within one clinical center | |
|  |  | |
|  |  | |
| 1c | Absolute SpPins and SnNouts  (An ‘Absolute SpPin’ is a diagnostic finding whose Specificity is so high that a Positive result rules in the diagnosis. An ‘Absolute SnNout’ is a diagnostic finding whose Sensitivity is so high that a Negative result rules out the diagnosis) | |
|  |  | |
|  |  | |
| 2a | Systematic review (with homogeneity) of Level > 2 diagnostic studies | |
|  |  | |
|  |  | |
| 2b | Exploratory cohort study with good reference standards; or clinical decision rule after derivation, or validated only on split-sample or databases | |
|  |  | |
|  |  | |
| 3a | Systematic review (with homogeneity) of studies Level ≥ 3b | |
|  |  | |
|  |  | |
| 3b | Non-consecutive study; or without consistently applied reference standards | |
|  |  | |
|  |  | |
| 4 | Case–control study, poor or non-independent reference standard | |
|  |  | |
|  |  | |
| 5 | Expert opinion without explicit critical appraisal, or based on physiology, bench research or ‘first principles’ | |
| *Grades of statement* | | |
| *Code* | *Quality of evidence* | *Definition* |
|  |  |  |
| A | High | Further research is very unlikely to change our confidence in the estimate of effect.   - Several high-quality studies with consistent results - In special cases: one large, high-quality multicenter trial |
|  |  |  |
|  |  |  |
| B | Moderate | Further research is likely to have an important impact on our confidence in the estimate of effect and may change the estimate.   - One high-quality study - Several studies with some limitations |
|  |  |  |
|  |  |  |
| C | Low | Further research is very likely to have an important impact on our confidence in the estimate of effect and is likely to change the estimate.   - One or more studies with severe limitations |
|  |  |  |
|  |  |  |
| D | Very low | Any estimate of effect is very uncertain.   - Expert opinion - No direct research evidence - One or more studies with very severe limitations |
|  |  |  |
| Note: A minus sign ‘−‘ may be added to denote evidence that fails to provide a conclusive answer because it is either (a) a single result with a wide confidence interval; or (b) a systematic review with considerable heterogeneity. Such evidence is inconclusive, and therefore can only generate Grade D recommendations. | | |
